# Tuning phenoxyl-substituted diketopyrrolopyrroles from quinoidal to biradical ground states through (hetero-)aromatic linkers†

**DOI:** 10.1039/d0sc05475e

**Published:** 2020-11-11

**Authors:** Rodger Rausch, Merle I. S. Röhr, David Schmidt, Ivo Krummenacher, Holger Braunschweig, Frank Würthner

**Affiliations:** Universität Würzburg, Institut für Organische Chemie Am Hubland 97074 Würzburg Germany wuerthner@uni-wuerzburg.de; Universität Würzburg, Center for Nanosystems Chemistry (CNC) Theodor-Boveri-Weg 97074 Würzburg Germany; Universität Würzburg, Institut für Anorganische Chemie, Institute for Sustainable Chemistry and Catalysis with Boron Am Hubland 97074 Würzburg Germany

## Abstract

Strongly fluorescent halochromic 2,6-di-*tert*-butyl-phenol-functionalised phenyl-, thienyl- and furyl-substituted diketopyrrolopyrrole (DPP) dyes were deprotonated and oxidised to give either phenylene-linked DPP1˙˙ biradical (*y*_0_ = 0.75) with a singlet open shell ground state and a thermally populated triplet state (Δ*E*_ST_ = 19 meV; 1.8 kJ mol^−1^; 0.43 kcal mol^−1^) or thienylene/furylene-linked DPP2q and DPP3q compounds with closed shell quinoidal ground states. Accordingly, we identified the aromaticity of the conjugated (hetero-)aromatic bridge to be key for modulating the electronic character of these biradicaloid compounds and achieved a spin crossover from closed shell quinones DPP2q and DPP3q to open shell biradical DPP1˙˙ as confirmed by optical and magnetic spectroscopic studies (UV/vis/NIR, NMR, EPR) as well as computational investigations (spin-flip TD-DFT calculations in combination with CASSCF(4,4) and harmonic oscillator model of aromaticity (HOMA) analysis). Spectroelectrochemical studies and comproportionation experiments further prove the reversible formation of mixed-valent radical anions for the DPP2q and DPP3q quinoidal compounds with absorption bands edging into the NIR spectral region.

## Abstract 2

By variation of spacer aromaticity, a spin crossover from thienylene/furylene-linked quinones DPP2q/DPP3q to phenylene-bridged biradical DPP1˙˙ (*y*_0_ = 0.75) with a singlet open shell ground state (Δ*E*_ST_ = 19 meV) was achieved.

## Graphical abstract

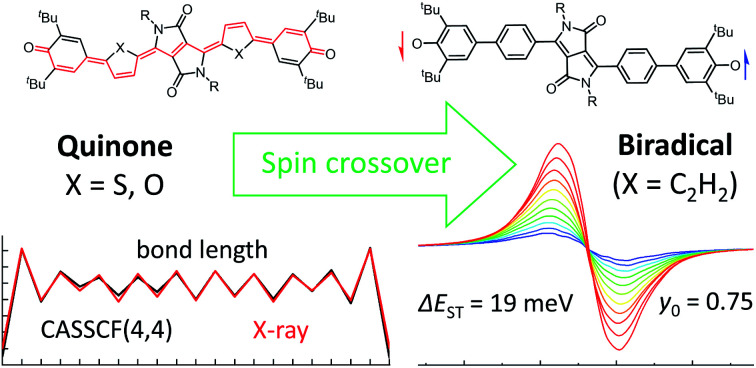

## 1. Introduction

**Scheme 1:**
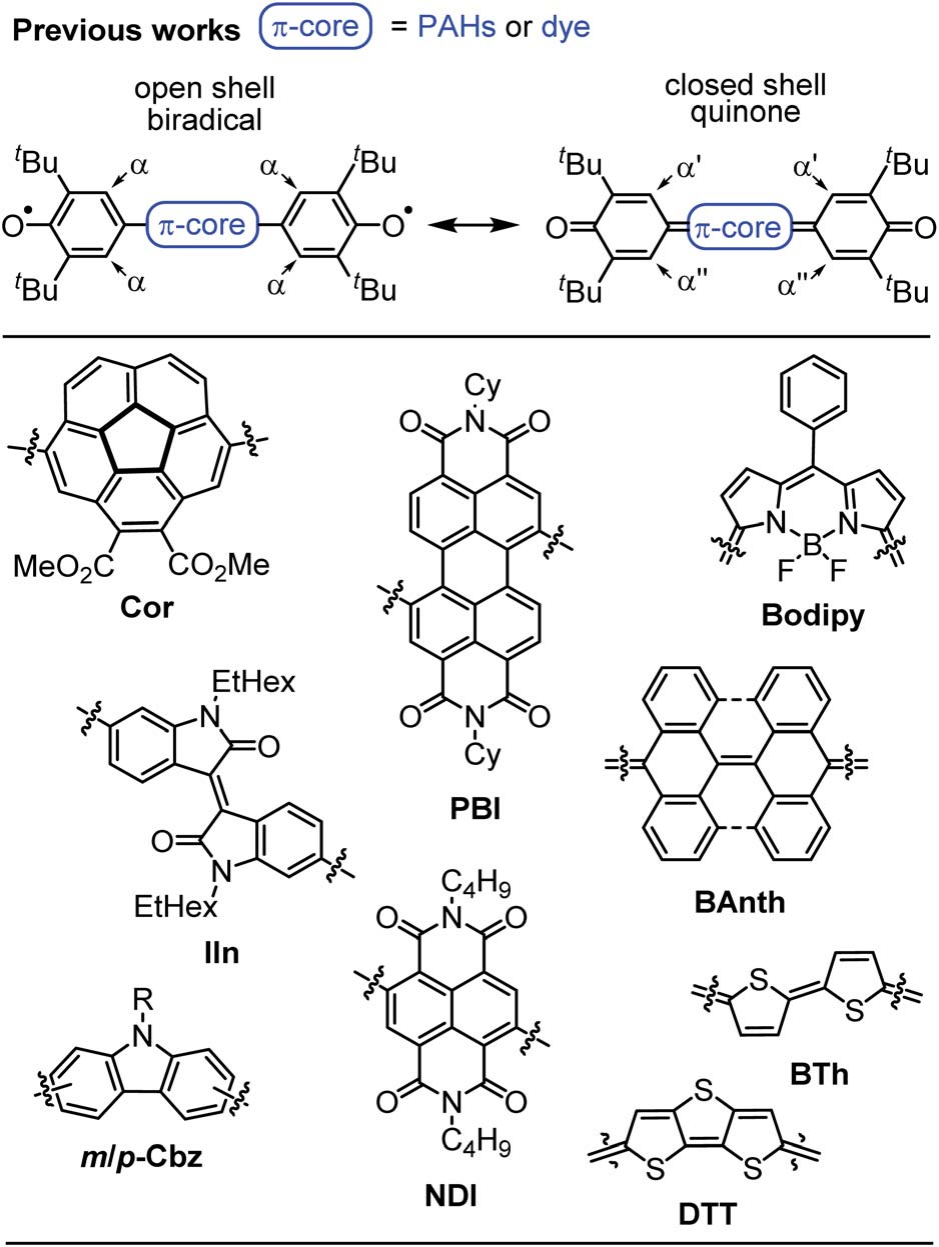
(Top) Open and closed shell resonance structures of Kekulé type biradicals and biradicaloids and (bottom) examples of π-extended quinones and biradicals based on planar and bowl shaped polycyclic aromatic hydrocarbons, pigment chromophores and oligomeric heteroaromatics.^18–25^

Despite the long history,^1^ open shell organic molecules have gained tremendous interest in recent years due to their unique electronic, magnetic and optical properties, which mainly arise from weakly coupled electron spins.^2^ Biradicals are the smallest units to allow investigations of intramolecular spin interactions.^3^ They commonly feature small HOMO–LUMO energy gaps,^4^ enhanced second hyperpolarisability,^5^ redox amphoterism^6^ and large two-photon absorption cross-sections.^7^ While on the one hand these properties make biradicals promising candidates for organic spintronics,^8^ molecular magnetism,^9^ energy storage^10^ and electronic devices,^11^ it is the unpaired electron spins, on the other hand, that drastically increase the reactivity and accelerate decomposition. Therefore, incorporation of biradicals into Kekulé-type quinoidal resonance structures is a common design strategy to stabilise these inherently highly reactive species. However, this brings up the question which factors favour biradicals and which ones quinodimethanes (≈biradicaloids), as well as how to distinguish experimentally between both of these singlet states.^2^ In general, quinodimethane-like compounds can be described by closed shell Kekulé structures, as open shell biradicals or as a superposition of both.^12,13^ Singlet biradicals are molecules with a singlet multiplicity of the lowest energy state but an open shell configuration and are described by the respective biradical character *y*_0_, which ranges from zero for closed shell to one for completely open shell compounds.^2^ They are further characterised by the singlet triplet energy gap Δ*E*_ST_, which typically lies between 10 and 500 meV (=0.96–48 kJ mol^−1^; 0.23–11.5 kcal mol^−1^).^14–17^ Within this material class, biradical(oid)s composed of π-systems substituted with two phenoxyl units are among the most outstanding representatives, due to the broad variety of accessible structures, which makes them an ideal model system for experimental and theoretical investigations (Scheme 1).^18–25^ In order to gain insight into the biradical character of these organic compounds, it is crucial to experimentally distinguish the biradical and quinone singlet state. As energy differences between these states tend to be very narrow, an even more careful choice of analytical methods is essential to uncover hidden discrepancies and draw solid conclusions. In general, biradicals show significantly broadened ^1^H NMR spectra^18,19^ and/or pronounced EPR signals^19,20^ due to thermal triplet state population. Quinones, in contrast, are commonly EPR silent and characterised by sharp NMR resonance signals.^21,22^ Additionally, the (in)equivalency of phenyl protons α (Scheme 1, top) can be used as an indicator for the molecules' rigidity.^20,22^ Furthermore, in order to experimentally access the singlet–triplet energy gap (Δ*E*_ST_), SQUID (superconducting quantum interference device) or variable temperature (VT) EPR can be utilised, of which the latter one commonly is performed in solution.^19,22^ In the solid state, further effects like enhanced intermolecular interactions have to be considered.^23^ X-ray analysis is a powerful method to shine light on the electronic ground state, since it allows an experimental bond length determination. Accordingly, the appearance of a distinct bond length alternation (BLA) is commonly a clear hint towards closed shell quinones,^20,22^ whereas its absence indicates a dominating open shell biradical character.^24^

Like for the experimental counterpart, also the theoretical description of biradicals requires sophisticated methods, since conventional (single reference) DFT is mainly suitable for closed shell compounds, monoradicals, decoupled pairs of local doublets or high spin states, but rather inappropriate to properly describe low spin (*i.e.* singlet) states in biradicals.^22,26^ Nevertheless, in case of very weakly interacting spin centres in biradicals, conventional DFT can lead to reasonable results in accordance with experimental findings (like for isoindigo derivative IIn),^19^ but has to be treated with caution. Due to an increasing number of compounds claimed to be open shell and a broad variety of methods applied – experimentally as well as theoretically – it is more than ever important to study molecules comprehensively because otherwise a comparison of investigated molecules like those collected in Scheme 1 becomes impossible. In this regard, only investigations applying the same theoretical (and ideally identical experimental) methods to all molecules allow relevant conclusions.^27^ Unfortunately, an increasing number of recent publications still utilises isolated characterisation methods and (completely) neglects double checking of the gained results by comprehensive methods.

In the past, several factors influencing the electronic ground state and hence enabling a spin crossover from closed shell quinoidal to open shell biradical of π-conjugated compounds derived from quinodimethanes in general and twofold phenoxyl substituted chromophores in particular have been investigated. In this regard, the impact of the number of bridging phenyl units,^28,29^ the connecting π-core (Scheme 1, bottom),^18–21,24,25^ hetero atom effects, the number of attached (donor) substituents and the steric demand of *ortho*-positioned alkyl chains protecting the radical centres in such systems^30–32^ has been investigated. Furthermore, based on numerous longitudinally and laterally extended zethrenes,^33–36^ Wu and coworkers as well as Juríček and coworkers have demonstrated, that the size of the π-scaffold can significantly influence the biradical character of these polycyclic aromatic hydrocarbons (PAH). Likewise, Baumgarten, Feng and Müllen and in particular Haley and coworkers utilised structural isomerism and the number of Clar sextets^37,38^ in various (di)indenoacenes^27,39–43^ to rationally finetune the Δ*E*_ST_ of these compounds. However, a spin crossover has so far been mainly observed upon incremental elongation of an acene-like series.^44^

**Scheme 2:**
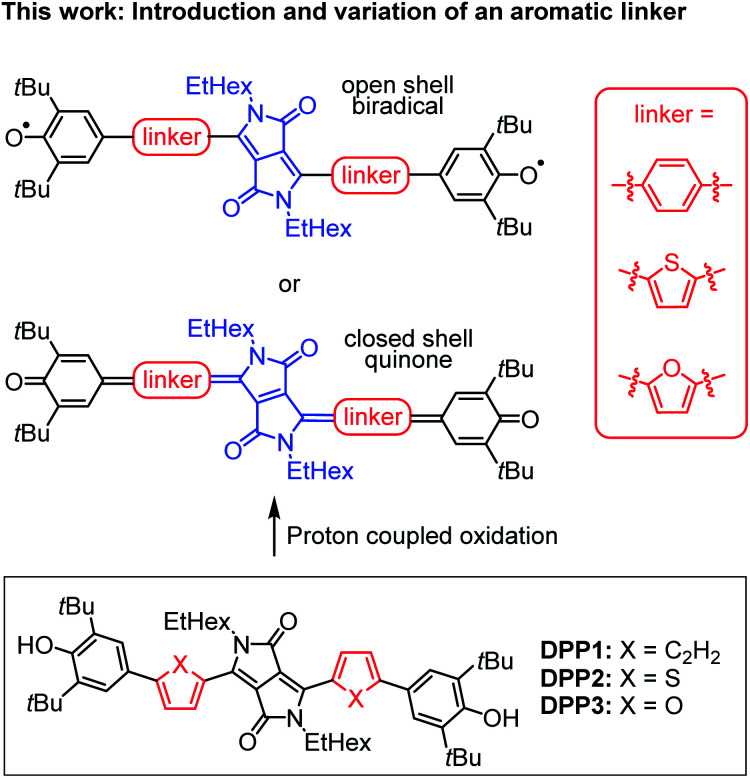
(Top) Schematic illustration of DPP bridged biradicals and quinones, as well as (bottom) structures of synthesised derivatives DPP1–3.

In this work, we present the first detailed study on a spin crossover for this important class of twofold phenoxyl functionalised π-scaffolds. Toward this goal we chose diketopyrrolopyrrole (DPP) as a core unit and carried out a simple linker variation, while maintaining the molecular size (Scheme 2). DPPs are highly versatile chromophores with tunable optical and electronic properties^45–49^ and outstanding chemical stability which explains their wide application in coatings^50^ and photoelectric devices.^51–59^ Furthermore, especially quinoidal DPPs and related oligo (hetero-)aromatic compounds have recently raised great attention as redox amphoteric dyes,^60,61^ in organic thin film transistors (OTFTs)^62–65^ or as near infrared (NIR) emitters.^66^ As DPPs are known both in the “aromatic” and the quinoidal conjugation, they are hence predestinated to study questions related to the biradical/quinone form.

As demonstrated by a recent contribution from Zheng and co-workers^32^ reporting on a similar series of DPPs (including DPP2 and DPP3 and their oxidised derivatives with just different alkyl chains) we were not alone with this idea. However, in contrast to the conclusion of these authors that these two molecules upon oxidation exhibit an open shell ground state, our high level quantum chemical calculations and multifaceted experimental methods revealed just the opposite: the ground state of these compounds is fully closed shell, *i.e.* quinoidal, which could be proven among other techniques by a X-ray crystal structure analysis for DPP3q. Most interestingly, for the oxidised species from the phenylene-spacered compound DPP1 (that was missing in the study of Zheng), indeed the desired spin crossover into the biradicaloid ground state can be observed. Thus, our systematic study of these biradicals and quinones with optical and magnetic spectroscopic methods as well as computational investigations with spin-flip TD-DFT calculations in combination with CASSCF(4,4) and harmonic oscillator model of aromaticity (HOMA) analysis allow to rationalise how the aromaticity of the linker unit tunes these DPP dyes from quinoidal to biradicaloid ground states. Accordingly, this is a lucky case where independently acquired results from two different laboratories on partly identical compounds enable insights on the importance of experimental and theoretical methods for obtaining conclusive results in this important field of research.^67^

## 2. Results and discussion

**Fig. 1:**
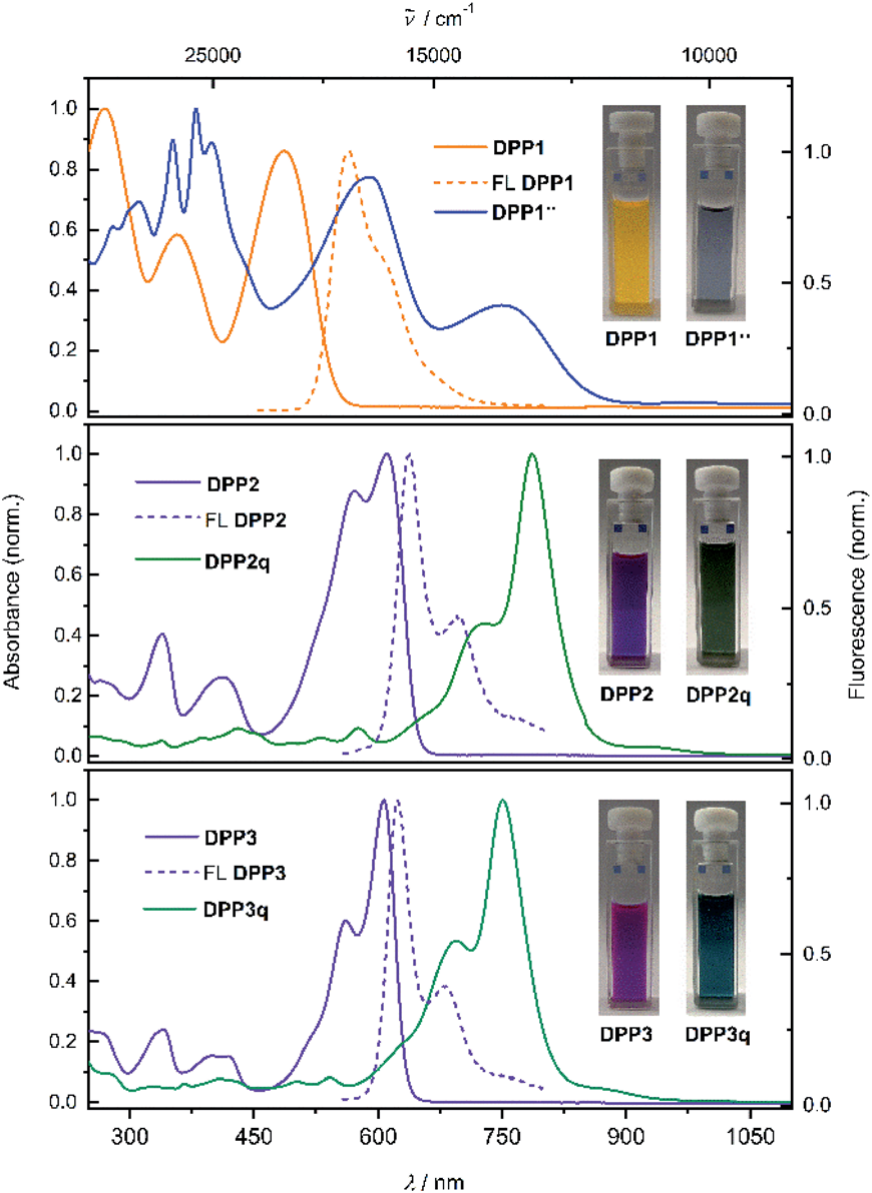
Normalised UV/vis/NIR absorption spectra (CH_2_Cl_2_, *c* ≈ 10 μM, rt) of DPP1 (orange solid line, top), DPP2 (purple solid line, middle) and DPP3 (purple solid line, bottom) as well as fluorescence spectra of DPP1–3 (orange and purple dashed lines, from the top). Also shown are the absorption spectra of biradical DPP1˙˙ (blue solid line, top), and quinones DPP2q (dark green solid line, middle) and DPP3q (green solid line, bottom) obtained from proton coupled oxidation of DPP1–3. Inset: photographs of the respective cuvettes.

Diketopyrrolopyrrole derivatives DPP1–3 were synthesised by Suzuki–Miyaura cross coupling of the respective literature known brominated DPP-precursors 1–3 with boronic ester 4 in 70%, 53% and 64% yield, respectively (Scheme S1a, ESI†). All new compounds were characterised by ^1^H and ^13^C NMR spectroscopy as well as high resolution mass spectrometry. Additionally, the structure of bisphenole DPP1 could be analysed by single crystal X-ray analysis (Fig. S4 and S5, ESI†).^68^ The optical properties of DPP1–3 in solution and in the solid state were investigated by UV/vis/NIR absorption and steady state fluorescence spectroscopy under ambient conditions (Fig. 1 and Table S3, ESI†).

The absorption spectrum of DPP1 in CH_2_Cl_2_ (Fig. 1 top, orange solid line) is characterised by broad absorption in the UV and visible spectral range with maxima located at 269, 356 and 486 nm. These absorption maxima are only slightly bathochromically shifted compared to the parent chromophore Ph_2_DPP (*λ*_abs_ = 466 nm).^69^ In contrast, heteroaromatic derivatives DPP2 (Fig. 1 middle, purple solid line) and DPP3 (Fig. 1 bottom, purple solid line) show absorption maxima at 610 nm (DPP2) and at 607 nm (DPP3) with well resolved vibronic progression, which are significantly redshifted compared to the unsubstituted parent compounds Fu_2_DPP and Th_2_DPP (*λ*_abs_ = 535–550 nm).^45,70^ The bathochromic shift observed in the UV/vis spectra suggests a pronounced electron-donating effect from the terminal 4-hydroxyphenyl units to the DPP chromophore cores. DPP1–3 show intense fluorescence at 565 nm for DPP1 (Δν̃_Stokes_ = 2580 cm^−1^, *Φ*_FL_ = 49%, Fig. 1 top, orange dashed line), at 638 nm for DPP2 (Δν̃_Stokes_ = 719 cm^−1^; *Φ*_FL_ = 38%, Fig. 1 middle, purple dashed line) and at 624 nm for DPP3 (Δν̃_Stokes_ = 449 cm^−1^; *Φ*_FL_ = 48%, Fig. 1 bottom, purple dashed line). It is noteworthy that despite of the lowering of the band gap in this series of dyes, fluorescence quantum yields remain high and comparable to those reported in literature for the respective unsubstituted chromophores Ph_2_DPP, Th_2_DPP or Fu_2_DPP with much larger band gaps.^45,69,70^

Dehydrogenation of bisphenols occurs easily using proton coupled oxidation which follows a concerted deprotonation–oxidation mechanism.^18,19,25^ Therefore, solutions of DPP1–3 were treated with basic tetra-*n*-butylammonium fluoride (TBAF) in order to study the occurring spectral changes. Gradual changes in the respective absorption spectra were monitored by UV/vis/NIR spectroscopy (Fig. S7, ESI†). Upon stepwise addition of a solution of TBAF in CH_2_Cl_2_, the intensity of the absorption bands of DPP1, DPP2 and DPP3 decreases with concomitant rise of absorption bands at longer wavelengths at 704 nm for DPP1, at 813 nm for DPP2 and at 789 nm for DPP3. The appearance of these bathochromically shifted absorption bands indicates the formation of the respective dianions DPP1^2−^, DPP2^2−^ and DPP3^2−^ and can be explained by the strong charge transfer (CT) from the electron-rich phenoxide substituents to the electron-poor DPP core. Such CT bands are commonly observed in halochromic systems.^18,19,25^ Notably, the immediate addition of an equimolar amount of trifluoroacetic acid to freshly prepared solutions of the dianions leads to a complete recovery of the absorption spectral signatures of the corresponding phenols (Fig. S7, ESI†) and hence reveals the reversibility of the deprotonation process. Additionally, during deprotonation titration experiments of the heteroaromatic derivatives DPP2 and DPP3, a concomitant rise of an additional NIR band at 1083 and 1063 nm, respectively, was observed, which does not appear upon titration of DPP1 (Fig. S7, ESI†). The appearance of these bands indicates the formation of mixed valent radical anions MV-DPP2 and MV-DPP3 upon autoxidation. As these mixed valent species arise upon oxidation of dianions DPP2^2−^ and DPP3^2−^ under ambient conditions, we monitored UV/vis/NIR absorption spectral changes over time (Fig. S8, ESI†) to study the extent of this oxidation process. Within 17 h (DPP2^2−^) and 4 h (DPP3^2−^), the bands attributed to the presence of radical anions MV-DPP2 and MV-DPP3 rise continuously, followed by a subsequent decrease. Analogous time-dependent monitoring of DPP1^2−^ (Fig. S8a and b, ESI†) merely shows a decrease of CT band intensity upon oxidation, which is much slower than the processes observed for the heteroaromatic derivatives. It is worth to mention, that there is no hint for the formation of an analogous mixed valent species upon autoxidation of DPP1^2−^. Taking the different extent and rates of the autoxidation processes into account, it can be concluded that the phenolate's sensitivity towards oxidation is decreasing in the order DPP3^2−^ > DPP2^2−^ > DPP1^2−^. This trend was proven to hold true for neutral bisphenoles DPP1–3 as well by cyclic voltammetry, although the furane and thiophene derivatives feature a quite similar redox behaviour (Fig. S28 and Table S4, ESI†). Accordingly, the disparity between heterocyclic DPP2/3 and DPP2q/3q (S ↔ O) caused by chalcogen effects is much lower than the differences (S/O ↔ Ph) to the aromatic phenyl system DPP1.

**Fig. 2:**
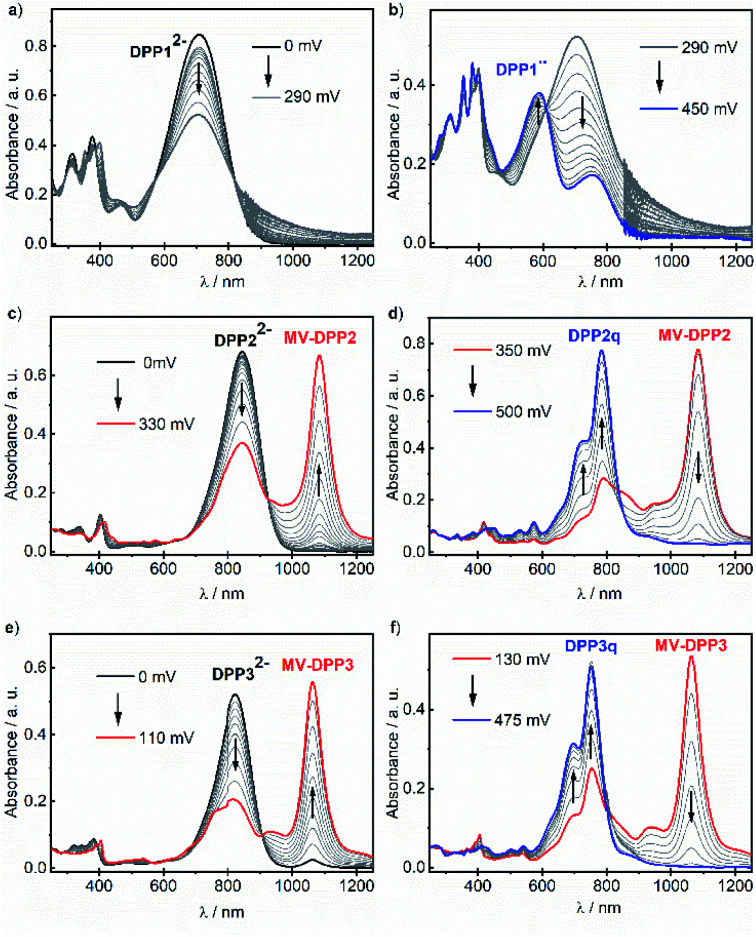
UV/vis/NIR absorption spectral changes of (a and b) DPP1^2−^, (c and d) DPP2^2−^ and (e and f) DPP3^2−^ (*c* ≈ 3 mM in CH_2_Cl_2_, rt, 0.2 M ^*n*^Bu_4_NPF_6_) upon electrochemical oxidation in a spectroelectrochemical setup. Arrows indicate spectral changes with increasing positive potential.

Our results indicate, that the first (aut-)oxidation step of DPP2^2−^ and DPP3^2−^ involves the formation of radical anions MV-DPP2 and MV-DPP3 by single electron transfer (SET). Apparently, a further defined oxidation process cannot be achieved by using ambient oxygen. Therefore, we applied electrochemical oxidation in order to study the stepwise oxidation process and gain further insight into the redox properties. Accordingly, the electro-optical properties of dianions DPP1^2−^, DPP2^2−^ and DPP3^2−^ were subsequently studied by spectroelectrochemistry (SEC, Fig. 2). Initial spectral changes observed for DPP2^2−^ and DPP3^2−^ mainly reproduce the transformations already monitored during their autoxidation (Fig. 2c, e and S8, ESI†). However, the NIR bands attributed to radical anions MV-DPP2 and MV-DPP3 vanish completely upon further raising the potential to 500 mV and 475 mV (*vs.* Pt pseudo reference electrode, Fig. 2d and f, red solid line), respectively, and simultaneously, the appearance of new absorption bands with maxima at 782 nm (DPP2) and 751 nm (DPP3) (Fig. 2d and f, blue solid line) can be observed, which resemble by spectral shape and vibronic structure those of DPP2 and DPP3, but are significantly red shifted. The formed species represent quinones DPP2q and DPP3q (*vide infra*) with outstanding high molar extinction coefficients, *i.e.* tripled and doubled compared to DPP2 and DPP3, respectively (Table S3, ESI†). Also for DPP1^2−^ a new optical signature (Fig. 2b, blue solid line) emerges upon electrochemical oxidation with increasing potential to 450 mV, which could not be observed during autoxidation experiments (Fig. S8, ESI†) and can be attributed to the formation of DPP1˙˙ (*vide infra*). The resulting absorption spectrum is significantly broadened in the visible region and shows pronounced panchromaticity with intense maxima at 380 and 588 nm and a band of lower intensity in the NIR region (749 nm). The appearance of several isosbestic points for all three dianions DPP1–3^2−^ indicates clearly defined oxidation processes. Our results indicate the formation of mixed valent radical anions MV-DPP2 and MV-DPP3 as well as twofold oxidised species DPP1˙˙, DPP2q and DPP3q by electrochemical oxidation of DPP1–3.

In order to enable in-depth studies, we attempted to obtain these species on a preparative scale as well by applying a suitable chemical oxidant. Accordingly, biradical DPP1˙˙ and quinones DPP2q and DPP3q were synthesised almost quantitatively by oxidation of DPP1 with potassium ferricyanide and by dehydrogenation of DPP2 and DPP3 using lead(iv) oxide (Scheme S1b, ESI†), which could not be applied to DPP1 as it caused decomposition. All products feature the same UV/vis/NIR absorption spectral signatures as observed for the electrochemically generated ones (Fig. S9, ESI†). By selective reduction of DPP2q and DPP3q with (Me_2_N)_2_C=C(NMe_2_)_2_ (TDMAE), as well as by comproportionation of DPP2^2−^ or DPP3^2−^ with DPP2q or DPP3q, respectively, we could furthermore generate mixed valent compounds MV-DPP2 and MV-DPP3 (Schemes S8–S11 and Fig. S12, S13, ESI†), which, however, could not be isolated. Nevertheless, the result proves that comproportionation of (extended) quinones and bisphenoxides offers an efficient and convenient access to highly desirable radical anions^71^ in appropriately functionalised chromophores.

**Fig. 3:**
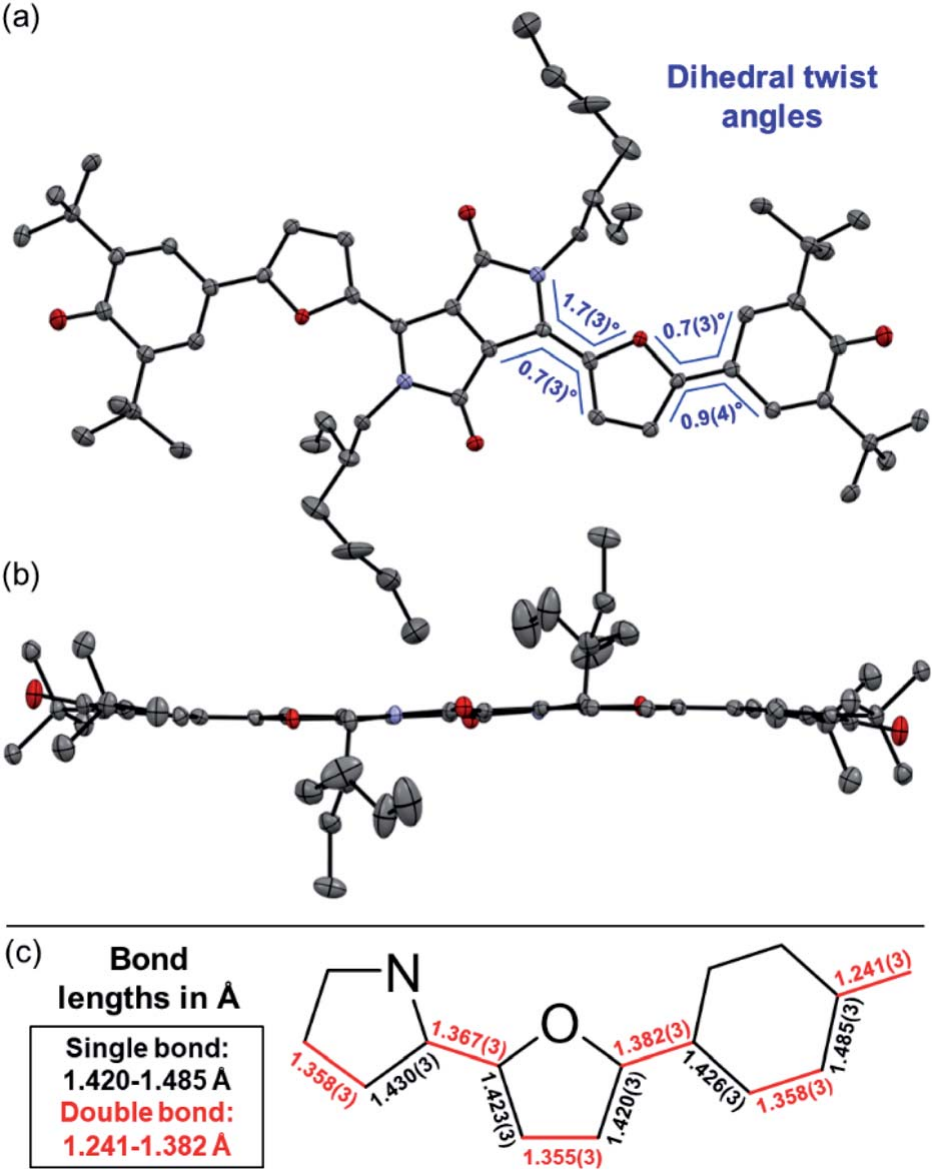
Solid state molecular structure of DPP3q in (a) top view and (b) side view as well as (c) selected bond lengths determined by single crystal X-ray diffraction (ellipsoids set to 50% probability, carbon gray, nitrogen blue, oxygen red). H atoms and solvent (MeOH) molecules are omitted for clarity.

Whereas DPP2q and DPP3q could be easily isolated out of solution by evaporation of the solvent (CH_2_Cl_2_) and even be crystallised (DPP3q, Fig. 3), attempts to isolate DPP1˙˙ failed and significant bleaching of the solution was observed instead even under inert conditions in degassed CCl_4_ and more accelerated upon raising temperature to reflux. For this reason, isolation or recrystallization was not successful as well. Hence, to quantify the stability of DPP1˙˙, DPP2q and DPP3q in solution and in the solid state under ambient conditions, time-dependent UV/vis/NIR absorption spectra were recorded. The spectral signatures of DPP2q and DPP3q in solution and in the solid state remain unchanged over several days (Fig. S10, ESI†), and thus prove the high stability of these compounds. In contrast, DPP1˙˙ decomposes within minutes in the solid state and within hours in solution as band intensities gradually decrease over time (Fig. S11, ESI†). By fitting the time-dependent data, a minimum half-life of 78 h in CCl_4_ and 39 h in CH_2_Cl_2_ was obtained for DPP1˙˙ at room temperature (Fig. S11 and Tables S1, S2, ESI†). Such a fast decomposition within the timescale of days compared to stable DPP2q and DPP3q, can be explained with a considerably higher reactivity of DPP1˙˙, presumably caused by the distinct biradical character. Life times in the range of hours to days are indeed typical for phenoxyl based biradicals.^18,25^

**Fig. 4:**
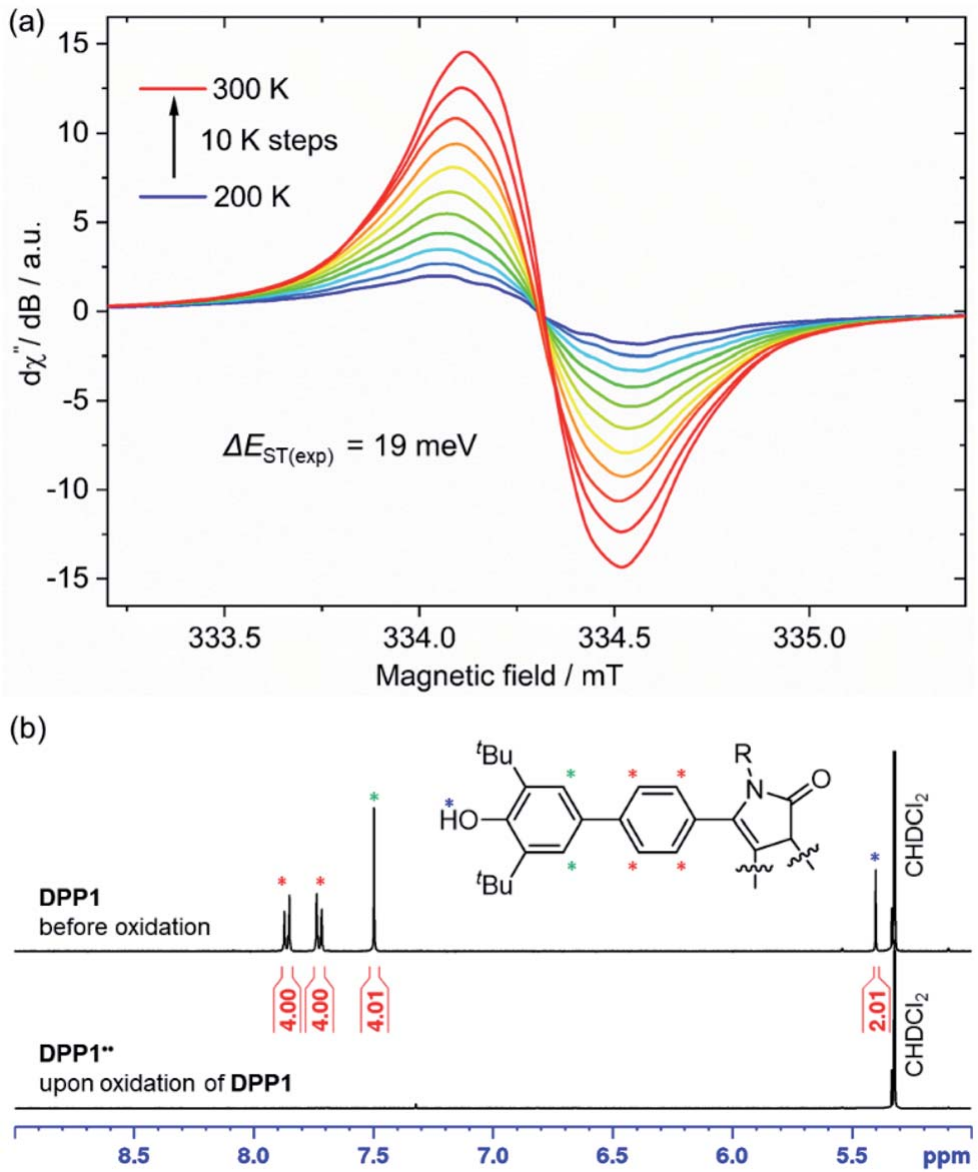
(a) Temperature dependence of the X-band EPR signal of DPP1˙˙ in CH_2_Cl_2_ (*c* ≈ 1 mM) and (b) aromatic region of the ^1^H NMR spectrum (400 MHz) of DPP1 (top) and DPP1˙˙ (bottom) in CD_2_Cl_2_ at rt.

Representatively for both quinones, the solid state structure of DPP3q could be determined by single crystal X-ray diffraction analysis (Fig. 3). Furane derivative DPP3q features an almost planar π-surface with negligible dihedral twist angles between 0.7(3)° and 1.7(3)° (Fig. 3a and b). In addition, a distinct bond length alternation over the whole chromophore can be observed (Fig. 3c), which is well in line with the quinoidal character of DPP3q concluded by NMR spectroscopy (*vide infra*). The parallel displaced chromophores with an average π–π-distance of 3.19 Å form staircase like strands, which are oriented in a herringbone-type packing (Fig. S6, ESI†). As DPP1˙˙ is stable for several hours in solution (CH_2_Cl_2_) and DPP2q and DPP3q show no significant decomposition, we were able to investigate the para- and diamagnetic properties of the compounds with electron paramagnetic resonance (EPR) and nuclear magnetic resonance (NMR) spectroscopy (Fig. 4).

DPP2q and DPP3q are virtually EPR silent (Fig. S31a and b, ESI†) in CH_2_Cl_2_ solution, but show a very weak signal in the solid state. However, temperature-dependent solid state EPR measurements in the range of 240 to 300 K did not reveal any significant signal intensity change (Fig. S31c and d, ESI†). Accordingly, no hints for thermal triplet state population were found, which proofs an insurmountable Δ*E*_ST_ and provides further evidence for a diamagnetic character. In contrast, DPP1˙˙ features a pronounced EPR signal, which is centred at *g*_iso_ = 2.0044 with a peak-to-peak line width of 3.8 G (Fig. 4a). Temperature-dependent EPR spectroscopy of DPP1˙˙ additionally allowed for a quantitative view on the energy difference between the singlet and triplet state. Double integration of EPR signals and data fitting according to the Bleaney–Bowers equation revealed a remarkably low Δ*E*_ST_ of 19 meV (1.8 kJ mol^−1^; 0.43 kcal mol^−1^, 2*J* = 147 cm^−1^) and hence indicates almost decoupled spin centres (Fig. S30, ESI†). As EPR signal integrals of DPP1˙˙ increase with temperature, an open shell singlet ground state can be concluded for DPP1˙˙. In compliance with the increasing amount of triplet species at higher temperature, also a pronounced broadening of resonance signals in the aromatic region of the respective ^1^H NMR spectra is observed, thereby confirming the substantial paramagnetic character of DPP1˙˙ at room temperature.^18,19,25^ Accordingly, the ^1^H NMR spectrum of DPP1˙˙ in CD_2_Cl_2_ at 298 K shows very broad signals (Fig. 4b), which significantly gain intensity upon decreasing the temperature to 180 K (Fig. S24, ESI†). This is in accordance with a biradical character in the ground state with a low energy difference to the NMR silent triplet state, which is thermally populated at higher temperature.

In contrast to DPP1˙˙, sharp signals are detected for DPP2q and DPP3q in the aromatic region of the respective ^1^H NMR spectra at room temperature. Notably, the resonance signals for the two heteroaryl protons are separated by 1.45 to 1.76 ppm (Fig. S16–S22, ESI†) with a significantly downfield shifted signal at 9.02 ppm (DPP2), 8.34 ppm (DPP3), 9.38 ppm (DPP2q) and 8.85 ppm (DPP3q). This shift can be explained by hydrogen bonding between a heteroaryl H atom and the carbonyl oxygen atom (Fig. S16–S22, ESI,† hydrogen bonded proton highlighted in blue).^61,62^ Accordingly, a *N*,*S*- and *N*,*O-cis* configuration can be concluded for all heteroaromatic derivatives, which was also corroborated by X-ray structure analysis in the case of DPP3q (Fig. 3). In addition, the resonance signal of the protons of the phenyl moiety in DPP2 (7.52 ppm) and DPP3 (7.63 ppm) with a singlet multiplicity splits up into two doublet signals for quinones DPP2q and DPP3q (Fig. S16–S22, ESI†). Thus, both protons are chemically not equivalent anymore, which can be explained by the formation of a double bond between the former phenyl and heteroaromatic unit.^22,32^ Therefore, the rotational freedom of the terminal phenyl unit is significantly hindered, as expected due to the quinoidal character of DPP2q and DPP3q. At an elevated temperature of 373 K, the resonance signals assigned to the heteroaryl protons of DPP3q remain sharp, whereas signals of DPP2q show a slight broadening (Fig. S25 and S26, ESI†). In particular, the resonance signals ascribed to the hydrogen bonded protons (*vide supra*) are well detectable, whereas the phenyl proton signal becomes broad in all heteroaromatic quinones.^32^ Accordingly, the different behavior of phenyl and heteroaryl proton signals upon heating can be explained by a rather rigid DPP core flanked by phenoxyl groups with thermally enhanced rotational freedom rather than by a thermal triplet population. Summarising, it can be concluded that the ground state of DPP2q and DPP3q is dominated by a closed shell character with a large, thermally insurmountable singlet–triplet energy gap (Δ*E*_ST_).

To further shine light onto the electronic ground state character of DPP1˙˙, DPP2q and DPP3q, quantum chemical calculations have been performed. In general, biradicals can be defined as molecular systems with two electrons occupying two (almost) degenerate molecular frontier orbitals.^2^ These orbital (near-)degeneracies result in wave functions, which are not dominated by a single configuration, but rather include several leading determinants. Since the accurate description of low-spin states in organic systems with partial open shell contributions requires at least two Slater determinants, biradicals usually cannot be described sufficiently using conventional density functional theory (DFT) or single-reference wavefunction-based methods,^26^ but considerably more precisely by applying the spin-flip (SF) TD-DFT approach.^72^ This method uses a single-determinant high-spin triplet state as a reference, which is well represented by a single Slater determinant. From that configuration, the target manifold of low-spin states (that is: singlets and low-spin triplet) is generated in a single excitation by applying a linear spin-flipping excitation operator 
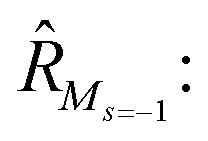
1
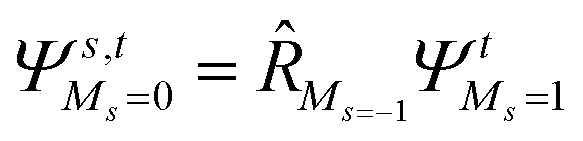


**Fig. 5:**
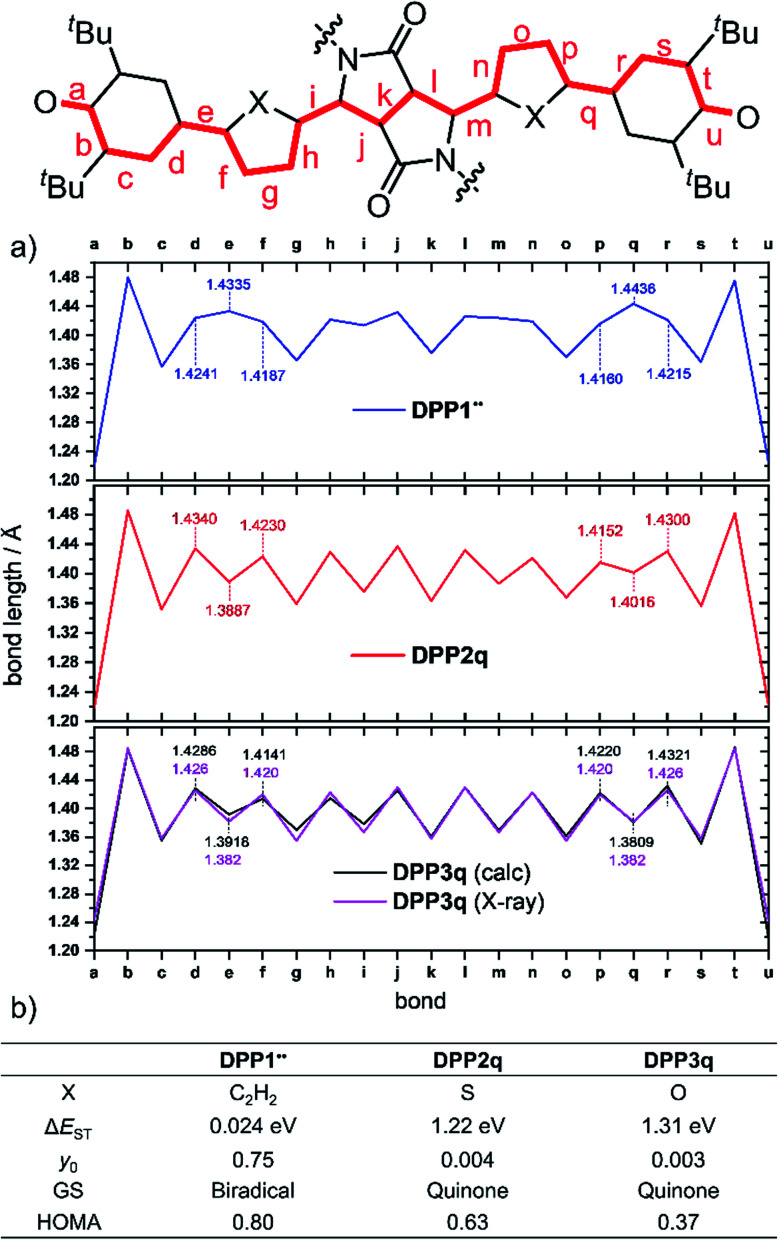
(a) Bond lengths in Å between both oxygen sites in DPP1˙˙, DPP2q and DPP3q (blue, red and black solid line, from the top) as well as (b) calculated biradical/quinoidal properties of DPP1˙˙, DPP2q and DPP3q, ((SF) TD-DFT and CASSCF(4,4)/def2-SVP level of theory). Also shown are the bond lengths determined experimentally by X-ray diffraction (purple solid line). GS = ground state.

Optimization of the formally first excited state and subsequent analysis of its character allows determination of the electronic configuration in the relaxed ground state structure of the molecule. The geometry optimization of DPP1˙˙, DPP2q and DPP3q has therefore been carried out in the framework of SF-TD-DFT employing the 50/50 functional (50% Hartree − Fock + 8% Slater^73^ + 42% Becke^74^ for exchange and 19% VWN^75^ + 81% LYP^76^ for correlation) along with the def2-SVP^77^ basis set (Fig. S35, ESI†). Dihedral angles between the terminal phenoxyl and bridging phenylene unit in DPP1˙˙ are significantly reduced to 11.14–14.73° compared to the parent bisphenole DPP1 (30.2–32.2°, Fig. S4, ESI†), which can be explained by increasing conjugation in the dehydrogenated state.^24^ In accordance with the single crystal X-ray results for DPP3q, the relaxed geometries of DPP2q and DPP3q exhibit negligible dihedral angles of only 0.5/0.6° and 0.2/0.4°, respectively. Furthermore, we observed a distinct, and continuous bond length alternation over the whole chromophore in DPP2q and DPP3q. In contrast, BLA is broken in DPP1˙˙ with significantly elongated bonds e and q indicating a much higher biradical character (Fig. 5a).

As evidenced by ^1^H NMR spectroscopy and X-ray analysis (*vide supra*), the *N*,*O*- and *N*,*S-cis* geometry – on which our calculations are based – is dominating in DPP2q and DPP3q. However, to complete our geometrical analysis, we performed optimisation of the *N*,*X-trans* geometries as well (Fig. S37, ESI†). The *trans* geometries were found to be 340 to 400 meV (=32.8–38.6 kJ mol^−1^; 7.84–9.22 kcal mol^−1^) higher in energy, making the *cis* conformation a thermodynamic global minimum. Notably, the impact of the conformation on the bond lengths is very large. Accordingly, BLA is significantly reduced in the hypothetical *trans* geometry which is in good agreement with the respective values obtained by conventional DFT.^32^ For this reason, the real bond length alternation was underestimated in the earlier study by Zheng and co-workers, which is based on the *trans* geometry.^32^

Additionally, analysis of the spin (*S*^2^ = 0) and the natural orbital occupation number was used to determine the electronic ground state configuration and biradical character *y*_0_.^78^ Both together indicate that the energetically most favorable electronic configuration is governed by a closed shell occupation of orbitals for DPP2q and DPP3q, while DPP1˙˙ is dominated by an open-shell singlet configuration and hence can be described as a singlet biradical.

In order to estimate the singlet–triplet energy gaps and quantify the singlet biradical character *y*_0_, single point CASSCF(4,4)/def2-SVP^75,77–82^ calculations were employed for all three relaxed geometries, confirming the SF-TD-DFT results. The resulting energy gap is calculated to be 24 meV (2.3 kJ mol^−1^, 0.54 kcal mol^−1^) in case of DPP1˙˙ and 1.22 eV (118 kJ mol^−1^, 28.1 kcal mol^−1^)/1.31 eV (126 kJ mol^−1^, 30.2 kcal mol^−1^) for DPP2q/DPP3q, respectively (Fig. 5b). The calculated Δ*E*_ST_ of DPP1˙˙ is in good accordance with the value obtained experimentally by temperature-dependent EPR spectroscopy. Accordingly, the value of *y*_0_ = 0.75 obtained for DPP1˙˙ clearly indicates the high biradical character of DPP1˙˙, while *y*_0_ values of 0.004 and 0.003 validate the closed shell configuration of the quinones DPP2q and DPP3q, respectively. These findings further support the structures derived from ^1^H NMR spectroscopy and X-ray analysis (*vide supra*). The low biradical character calculated for DPP2q and DPP3q is in contrast to the recent conclusions of Zheng and co-workers^32^ who reported a pronounced biradical character of *y*_0_ = 0.64–0.65 for related dyes bearing just different alkyl chains. However, these values were calculated by employing conventional DFT calculations, which become less precise in case of stronger spin interactions and low spin states (*vide supra*),^26^ as they are observed in DPP2q and DPP3q.

For the purpose of investigating the role of aromaticity as a key factor for the stabilization of the open shell configuration in DPP1˙˙, the “harmonic oscillator model of aromaticity” (HOMA)^83,84^ value was calculated for all three linkers. HOMA takes into account the deviation of bond length from an “optimal” value expected for a fully aromatic system.^83,84^2
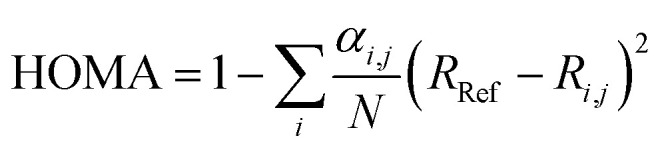
Here, *j* is the atom next to atom *i*, *N* denotes the total number of atoms, *α* and *R*_Ref_ are pre-calculated constants which are presented in the original article for each type of atom pair.^84^ A perfectly aromatic compound thereby has a HOMA value of 1, whereas a non-aromatic compound has a value of 0 or below. For the phenyl linker in DPP1˙˙ a value of 0.8 was obtained, which clearly indicates the presence of an aromatic benzene ring, thereby stabilising the biradical configuration, while in DPP2q and DPP3q, HOMA indices of 0.63 and 0.37, respectively, hint towards a much stronger quinoidal character (Fig. 5b). All theoretical findings are hence in agreement with the experimental results and our interpretation.

## 3. Conclusion

In conclusion, we reported a series of three with two 2,6-di-*tert*-butylphenoxy groups functionalised diketopyrrolopyrrole dyes DPP1–3 that on first glance look very similar, but upon deprotonation and oxidation afford electronically very distinct compounds, *i.e.* biradical DPP1˙˙ and quinones DPP2q and DPP3q. Our comprehensive optical and magnetic spectroscopic studies demonstrate the spin crossover from closed shell quinones DPP2q and DPP3q to a singlet open shell biradical DPP1˙˙ by simple linker variation. Thus, the heteroaromatic thiophene or furane linkers between the DPP core and phenoxyl substituent favour a closed shell ground state, thereby endowing DPP2q and DPP3q with bench stability and quinoidal structures as evidenced by X-ray diffraction (DPP3q) and UV/vis/NIR absorption, EPR and NMR spectroscopy as well as by quantum chemical calculations based on the spin-flip TD-DFT and CASSCF(4,4) level of theory. In contrast, the “isolating” strongly aromatic phenylene bridge endows derivative DPP1˙˙ with biradical properties with a very small singlet–triplet-energy gap of 19 meV (1.8 kJ mol^−1^; 0.43 kcal mol^−1^) and a large biradical character of *y*_0_ = 0.75. As a consequence, DPP1˙˙ loses stability and decomposes within the timescale of days in solution. The aromatic character of the bridging units in DPP1˙˙, DPP2q and DPP3q was investigated using HOMA index values, which show that the established order of aromaticity decrease (phenyl > thiophene > furane) applies for such biradicaloid systems with a central dye unit. Accordingly, we conclude that Clar's sextet rule^38^ also offers a design principle to derive open shell colourants with central dye and pigment units. In our example only DPP1˙˙ with four benzenoid sextets prevailed in an open shell configuration as a biradical, whereas energy gains through non-benzenoid heteroaromatic furane or thiophene linkers were not large enough to counteract the transformation of the π-electron system into a fully conjugated quinoidal scaffold.

## Conflicts of interest

There are no conflicts to declare.

## Supplementary Material

SC-012-D0SC05475E-s001

SC-012-D0SC05475E-s002
