# Supplementary material for: Tuning phenoxyl-substituted diketopyrrolopyrroles from quinoidal to biradical ground states through (hetero-)aromatic linkers
Source: Chem Sci. 2020 Nov 11;12(2):793–802. doi: 10.1039/d0sc05475e (PMC8179021; doi:10.1039/d0sc05475e)
Supplement: SC-012-D0SC05475E-s001 [file SC-012-D0SC05475E-s001.pdf]

Electronic Supplementary Material (ESI) for Chemical Science.
This journal is © The Royal Society of Chemistry 2020

Electronic Supplementary Information

Tuning phenoxyl-substituted diketopyrrolopyrroles from quinoidal to biradical ground states through (hetero-)aromatic linkers

Rodger Rausch[a], Merle I. S. Röhr[b], David Schmidt[ab], Ivo Krummenacher[c], Holger Braunschweig[c], and Frank Würthner*[ab]

[a] *Universität Würzburg, Institut für Organische Chemie, Am Hubland, 97074 Würzburg, Germany*

[b] *Universität Würzburg, Center for Nanosystems Chemistry (CNC), Theodor-Boveri-Weg, 97074 Würzburg, Germany*

[c] *Universität Würzburg, Institut für Anorganische Chemie and Institute for Sustainable Chemistry and Catalysis with Boron, Am Hubland, 97074 Würzburg, Germany*

* *e-mail: wuerthner@uni-wuerzburg.de*

Table of contents

1 Materials and methods S3

2 Syntheses S6

2.1 Synthesis of **DPP1** S7

2.2 Synthesis of **DPP2** S8

2.3 Synthesis of **DPP2q** S9

2.4 Synthesis of **DPP3** S10

2.5 Synthesis of **DPP3q** S11

2.6 Preparation of **DPP1••** sample S12

3 Mass spectrometry S13

4 Single crystal X-ray diffraction S14

5 UV/vis/NIR spectroscopy and spectroelectrochemistry S18

6 NMR spectroscopy S26

7 Band gap analysis and cyclic voltammetry (CV) S34

8 EPR spectroscopy S38

9 Computational chemistry S40

10 References S44

1 Materials and methods

Materials. Chemicals, reagents and solvents were purchased from commercial suppliers. Column chromatography was performed on silica gel (particle size 0.040–0.063 mm) with freshly distilled solvents as eluents. Tetrabutylammonium fluoride was purchased as its trihydrate but had a significantly higher water content due to its hygroscopic character. DPP derivatives **1**[S1], **2**[S2], and **3**[S3] as well as boronic ester **4**[S4] were synthesized according to literature known procedure. All other commercially available reagents and solvents were of reagent grade and used without further purification.

NMR Spectroscopy. ^{1}H, ^{11}B and ^{13}C NMR Spectra were recorded on a Bruker Avance III HD 400 or Bruker Avance III HD 600 spectrometer. ^{13}C NMR Spectra are broad band proton decoupled. Chemical shifts (*δ*) are listed in parts per million (ppm) and are reported relative to tetramethylsilane (TMS). Spectra are referenced internally to residual proton solvent resonances ($CDCl_3$: δ = 7.26, DMSO-d_6: δ = 2.50, CD_2Cl_2: δ = 5.32) or natural abundance carbon resonances ($CDCl_3$: δ = 77.00, CD_2Cl_2: δ = 53.84). ^{11}B NMR spectra were referenced to external $BF_3 \bullet Et_2O$. Coupling constants (*J*) are quoted in Hertz (Hz).The data are presented as follows: chemical shift, multiplicity (s = singlet, d = doublet, t = triplet, q = quartet, m = multiplet and/or multiple resonances, br = broad), coupling constant in Hertz (Hz), and integration.

Mass Spectrometry. MALDI-TOF mass spectra were recorded on a Bruker Daltronik GmbH (Autoflex II) mass spectrometer using trans-2-[3-(4-*tert*-butylphenyl)-2-methyl-2-propenylidene]malononitrile (DCTB) as matrix. High resolution ESI-TOF mass spectrometry was carried out on a microTOF focus instrument (Bruker Daltronik GmbH).

UV/vis/NIR Absorption Spectroscopy. The solvents for the spectroscopic measurements were of spectroscopic grade. UV/vis/NIR absorption spectra were recorded on a Perkin Elmer Lamda 950 or a Jasco V-670 spectrometer. Measurements in solution were carried out using quartz cuvettes with path lengths of 10 mm at ambient temperature. Measurements in solid state were performed on freshly spin coated thin films on quartz wavers at ambient temperature ($c = 2 \cdot 10^{-3}$ M, 25 µL, ambient temperature, rpm = 3000, t = 30 s).

UV/vis/NIR Spectroelectrochemistry. UV/vis/NIR spectroelectrochemical experiments in reflection mode were performed using an Agilent Cary 5000 Spectrometer and a self-made sample compartment with a layer thickness of 100 µm, consisting of a cylindrical PTFE cell, a sapphire window and an adjustable three in one electrode (6 mm platinum disc working electrode, 1 mm platinum counter and pseudo reference electrode).

Titration Experiments. Unless otherwise specified, a solution of TBAF · 3 H_2O (c = 1.90 · 10^{-3} M) in CH_2Cl_2 was added to the respective dye solution ($c \approx 2.85 \cdot 10^{-5}$ M) in CH_2Cl_2 at room temperature according to the below mentioned equivalents (1.00 equiv. = 15.0 µL) and filled up to 3.00 mL with pure CH_2Cl_2. Due to a possibly increase water content of TBAF it cannot be excluded that the absolute TBAF amount was slightly lower than assumed.

Cyclic Voltammetry. Cyclic voltammetry (CV) and square wave voltammetry (SWV) were performed using a standard commercial electrochemical analyzer (EC epsilon; BAS Instruments, UK). A Pt disc electrode was used as a working electrode, platinum wire as a counter electrode and Ag^+/Ag as a reference electrode, at a scan rate of 100 mV/s at room temperature. The compounds were dissolved in dichloromethane, and tetrabutylammonium hexafluorophosphate ($^n Bu_4NPF_6$) was added as an electrolyte to give a concentration of 0.1 M. The supporting electrolyte was recrystallized from ethanol/water and dried under vacuum. The oxidation potentials were referenced against the ferrocenium/ferrocene redox couple (Fc^+/Fc = 0.00 V).

Fluorescence Spectroscopy. Fluorescence spectra and fluorescence quantum yields were recorded with a PTI QM-4/2003 spectrometer (Photon Technology International, USA) as an average out of 4 equidistant excitation wavelengths relatively to the references *N*-(7-(diethylamino)-3*H*-phenoxazine-3-ylidene)-*N*-methylmethanaminium perchlorate[S5] (Φ_{Fl} = 0.11 in EtOH) „Oxazin 1“ (for **DPP2** und **DPP3**) und *N,N*-(2,6-Di-*iso*-propylphenyl)-1,6,7,12-tetraphenoxyperylene-3,4:9,10-tetracarboxylic acid bisimide[S6] (Φ_{Fl} = 0.96 in chloroform) „Perylene/ Lumogen red“ (for **DPP1**) in highly diluted solutions (OD < 0.05)[S7]. Measurements in solution were carried out using quartz cuvettes (Hellma optics, Germany) with path lengths of 10 mm and spectroscopic solvents at ambient temperature and atmosphere.

Computational chemistry. Spin-Flip TDDFT calculations have been carried out using the Q-Chem5.0 program package.[S8] (Ref1) For CASSCF calculations, the “Brilliantly Advanced General Electronic-structure Library” (BAGEL) by Shiozaki and coworkers[S9] has been employed. Starting orbitals have been generated by performing HF calculations and in the case of excited state calculations, they have been optimized in a state-averaged manner with equal weights for S0 and S1 state.

Single Crystal X-Ray Diffraction. Single crystal X-ray diffraction data for **DPP3q** were collected at 100 K on a Bruker X8APEX-II diffractometer with a CCD area detector and multi-layer mirror monochromated MoK_α radiation. Single crystal X-ray diffraction data for **DPP1** were collected on a Bruker D8 Quest Kappa Diffractometer with a Photon100 CMOS detector and multi-layered mirror monochromated CuK_α radiation. The structures were solved using direct methods, expanded with Fourier techniques and

refined with the Shelx software package.[S10] All non-hydrogen atoms were refined anisotropically. Hydrogen atoms were included in the structure factor calculation on geometrically idealized positions. Crystallographic data have been deposited with the Cambridge Crystallographic Data Centre as supplementary publication no. CCDC 2001481 (**DPP3q**) and CCDC 2001480 (**DPP1**). These data can be obtained free of charge from The Cambridge Crystallographic Data Centre via www.ccdc.cam.ac.uk/data_request/cif.

EPR Spectroscopy. EPR measurements at X-band (9.38 GHz) were carried out using a Bruker ELEXSYS E580 CW EPR spectrometer equipped with an Oxford Instruments helium cryostat (ESR900) and a MercuryITC temperature controller. The spectral simulations were performed using MATLAB 8.6 and the EasySpin 5.0.18 toolbox.[S11]

2 Syntheses

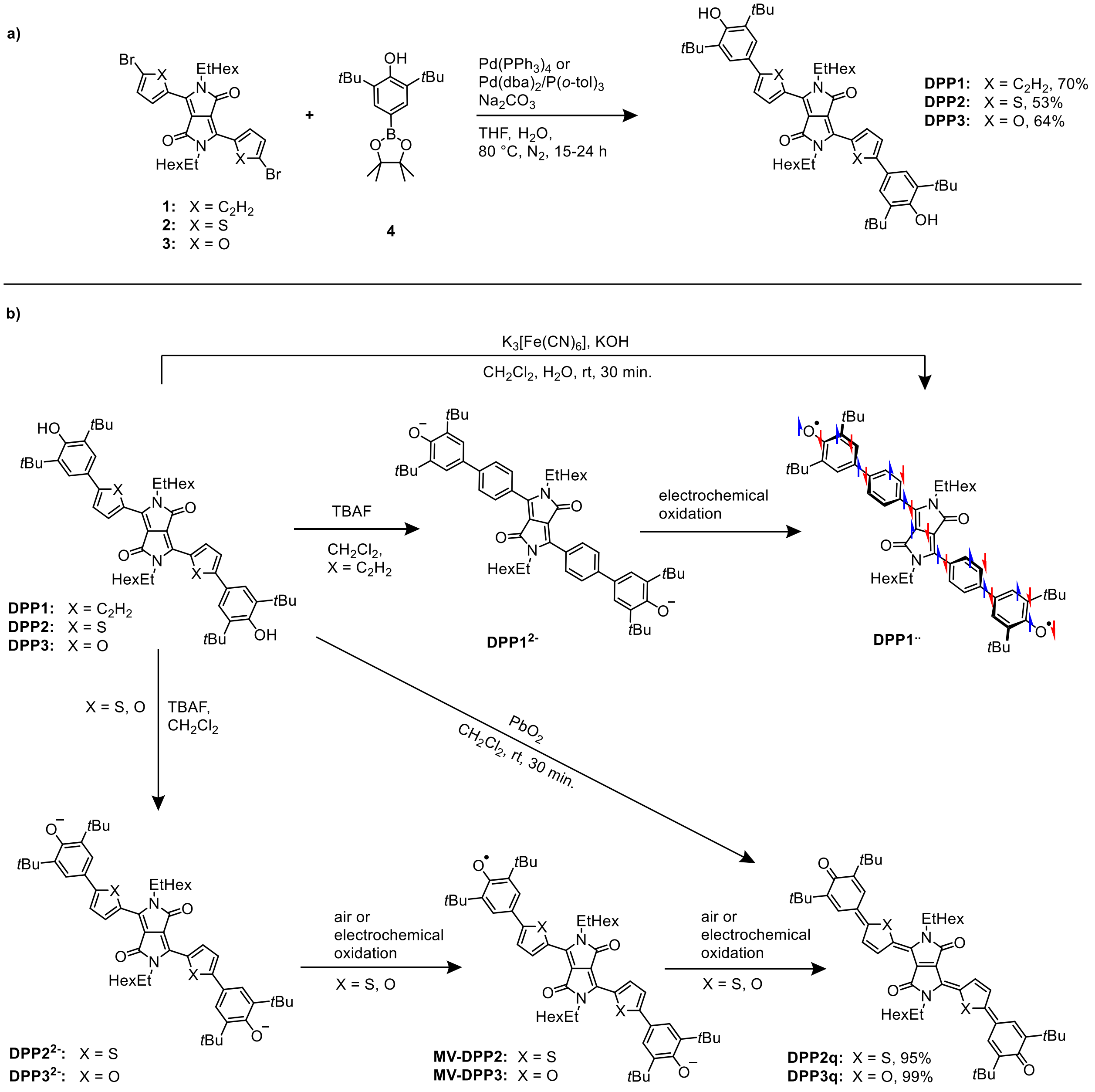

Scheme 1 (a) Synthetic routes to diketopyrrolopyrrole derivatives **DPP1**, **DPP2** and **DPP3**. (b) Synthesis of biradical **DPP1˙˙** and quinones **DPP2q** and **DPP3q**, as well as generation of dianions **DPP1–3**$^{2-}$ and radicalanions **MV-DPP2** and **MV-DPP3**. Blue and red arrows in **DPP1˙˙** indicate spin polarization and illustrate antiferromagnetic coupling.

2.1 Synthesis of DPP1

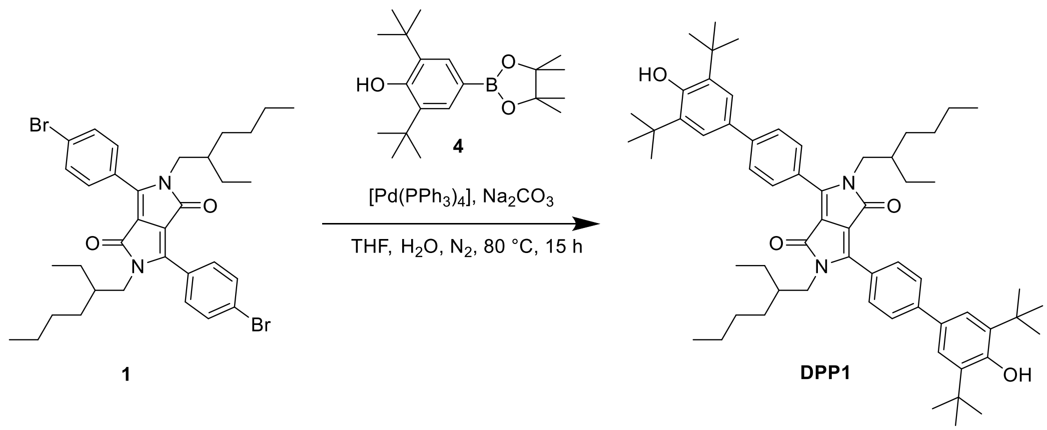

Scheme S2 Synthesis of **DPP1** by Suzuki-Miyaura cross-coupling.

Sodium carbonate (490 mg, 4.60 mmol, 17.7 equiv.), **1** (175 mg, 261 µmol, 1.00 equiv.), and tetrakis(triphenylphosphine)palladium(0) (20.0 mg, 17.0 µmol, 6.67 mol%) were dissolved in a degassed mixture of THF/H_2O (10 mL/ 2mL) under an atmosphere of nitrogen. After the mixture was heated to 50 °C for 30 min., boronic ester **4** (176 mg, 530 µmol, 2.03 equiv.) dissolved in THF (5 mL) was added and the reaction mixture was stirred at 80 °C for 15 h. Subsequently, the suspension was cooled down to room temperature and the crude product was extracted with CH_2Cl_2 (3 x 20 mL). The combined organic layers were washed with hydrochloric acid (1 M), water, dried over $MgSO_4$ and the solvent was removed under reduced pressure. The crude product was purified by silica gel column chromatography (gradient from hexane/ CH_2Cl_2 (1:1) to pure CH_2Cl_2) to yield **DPP1** as a bright orange solid.

Yield: 168 mg (183 µmol, 70 %). Melting point: 290 – 291 °C. ^{1}H NMR (400 MHz, CD_2Cl_2): δ = 0.72 – 0.77 (m, 6 H, C*H*3), 0.77 – 0.82 (m, 6 H, C*H*3), 1.04 – 1.39 (m, 18 H, βC*H,* alkyl-C*H2*), 1.51 (s, 36 H, C(C*H*3)3), 3.74 – 3.85 (m, 4 H, αC*H2*), 5.40 (s, 2 H, O*H*), 7.50 (s, 4 H, aryl-C*H*), 7.73 (d, 4 H, aryl-C*H*, $^3J_{HH}$ = 8.6 Hz), 7.86 (d, 4 H, aryl-C*H*, $^3J_{HH}$ = 8.6 Hz). ^{13}C NMR (101 MHz, CD_2Cl_2): δ = 10.7, 14.2, 23.2, 24.2, 28.8, 30.4, 30.7, 34.9, 39.0, 45.3, 110.2, 124.4, 127.0, 127.3, 129.5, 131.6, 137.0, 144.9, 148.4, 154.7, 163.1. HRMS (ESI-TOF, pos. mode, $CHCl_3$/MeCN 1/1): calculated for $C_{62}H_{84}N_2O_4^+$: *m/z* 920.6426 $[M]^+$, found 920.6410. MS (MALDI-TOF, pos. mode, DCTB 3:1 in $CHCl_3$): calculated for $C_{62}H_{84}N_2O_4^+$: *m/z* 920.6426 $[M]^+$, found 920.514. UV/Vis (DCM, $c = 9.80 \cdot 10^{-6}$ M): λ_{max} [nm] (ε_{max} [L mol^{-1} cm^{-1}]) = 269 (35000), 356 (20400), 486 (30100). IR (ATR): $\tilde{\nu}$ [cm^{-1}] = 3438 (s, $\nu_{O\text{-}H,str}$), 2951 (s, $\nu_{C\text{-}H,str}$), 2907 (s, $\nu_{C\text{-}H,str}$), 2855 (s, $\nu_{C\text{-}H,str}$), 1662 (vs, $\nu_{C=O,str}$). Fluorescence (DCM, $\lambda_{ex.}$ = 430 nm): λ_{max} = 565 nm (ϕ = 0.49 ± 0.07, standard: Perylene Orange).

2.2 Synthesis of DPP2

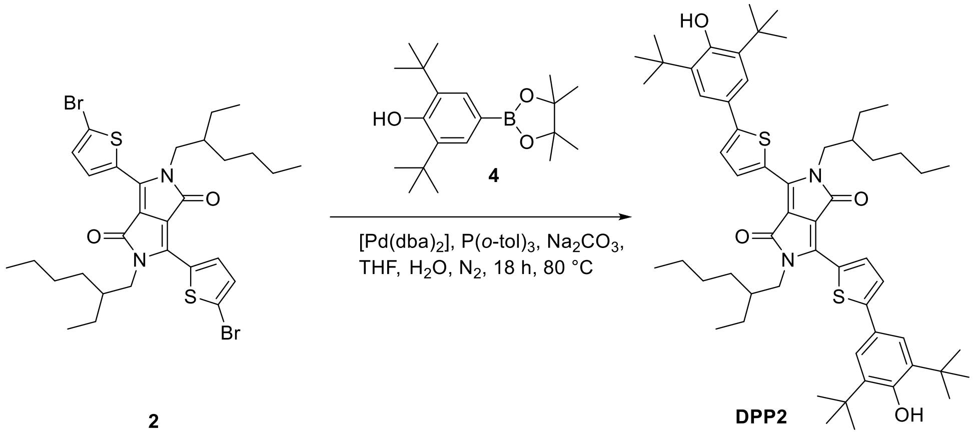

Scheme S3 Synthesis of **DPP2** by Suzuki-Miyaura cross-coupling.

Sodium carbonate (825 mg, 7.78 mmol, 17.7 equiv.), **2** (300 mg, 440 µmol, 1.00 equiv.), bis(dibenzylideneacetone)palladium(0) (17.0 mg, 29.4 µmol, 6.67 mol%), boronic ester **4** (366 mg, 1.10 mmol, 2.50 equiv.) and tri(*o*-tolyl)phosphine (18.0 mg, 58.8 µmol, 12.7 mol%) were dissolved in a degassed mixture of THF/H_2O (10 mL/ 2mL) under an atmosphere of nitrogen. After the mixture was heated to 80 °C for 18 h and cooled down to room temperature, water (30 mL) was added and the crude product was extracted with CH_2Cl_2 (3 x 20 mL). The combined organic layers were washed with hydrochloric acid (1 M), water, dried over $MgSO_4$ and the solvent was removed under reduced pressure. The crude product was purified by silica gel column chromatography (eluent: hexane/ CH_2Cl_2 (1:1)) to yield **DPP2** as a dark red solid.

Yield: 219 mg (235 µmol, 53 %). Melting point: 247 – 248 °C. ^{1}H NMR (400 MHz, CD_2Cl_2): δ = 0.87 (t, 6 H, C*H*$_3$, $^3J_{HH}$ = 7.2 Hz), 0.93 (t, 6 H, C*H*$_3$, $^3J_{HH}$ = 7.6 Hz), 1.24 – 1.46 (m, 16 H, C*H*$_2$), 1.49 (s, 36 H, C(C*H*$_3$)$_3$), 1.90 – 2.03 (m, 2 H, βC*H*), 4.00 – 4.15 (m, 4 H, αC*H*$_2$), 5.49 (s, 2 H, O*H*), 7.38 (d, 2 H, heteroaryl-C*H,* $^3J_{HH}$ = 4.1 Hz), 7.52 (s, 4 H, aryl-C*H*), 9.02 (d, 2 H, heteroaryl-C*H*, $^3J_{HH}$ = 4.1 Hz). ^{13}C NMR (101 MHz, CD_2Cl_2): δ = 10.8, 14.2, 23.5, 24.1, 25.0, 28.9, 30.3, 30.4, 34.8, 39.8, 46.1, 108.1, 123.4, 123.7, 125.1, 128.1, 132.1, 137.2, 137.3, 139.9, 151.7, 155.4, 162.1. HRMS (ESI-TOF, pos. mode, MeCN/ $CHCl_3$ 1/1): calculated for $C_{58}H_{80}N_2O_4S_2^+$: *m/z* 932.5554 [M]$^+$, found 932.5534. MS (MALDI-TOF, pos. mode, DCTB 3:1 in $CHCl_3$): calculated for $C_{58}H_{80}N_2O_4S_2^+$: *m/z* 932.555 [M]$^+$, found 932.437. UV/Vis (DCM, c = 1.01 · 10^{-5} M): λ_{max} [nm] (ε_{max} [L mol^{-1} cm^{-1}]) = 572 (48300), 610 (55000). IR (ATR): $\tilde{\nu}$ [cm^{-1}] = 3625 (s, $\nu_{O\text{-}H,str}$), 2952 (s, $\nu_{C\text{-}H,str}$), 2926 (s, $\nu_{C\text{-}H,str}$), 2867 (s, $\nu_{C\text{-}H,str}$), 1660 (vs, $\nu_{C=O,str}$). Fluorescence (DCM, $\lambda_{ex.}$ = 540 nm): λ_{max} = 638 nm (ϕ = 0.39 ± 0.03, standard: Oxazine 1).

2.3 Synthesis of DPP2q

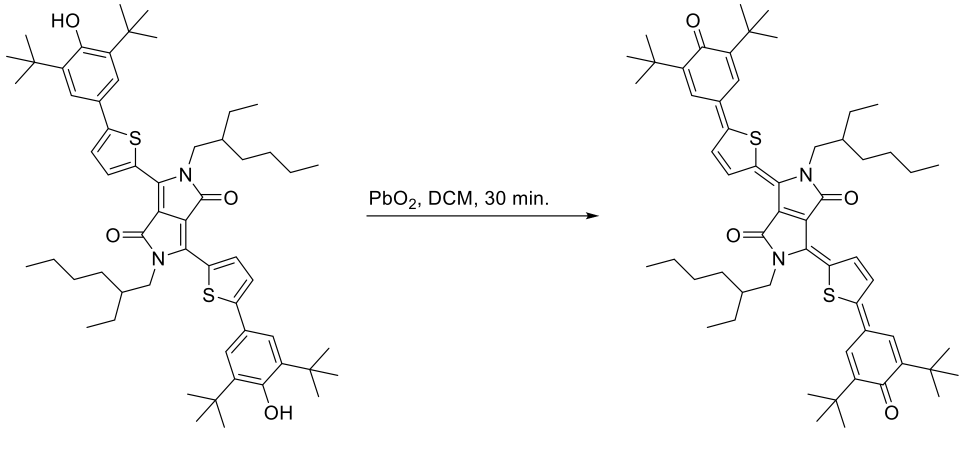

Scheme S4 Synthesis of **DPP2q** by oxidation of **DPP2** with lead(IV)oxide.

Lead(IV) oxide (210 mg, 878 µmol, 14.0 equiv.) and **DPP2** (59.0 mg, 63.0 µmol, 1.00 equiv.) were suspended in dichloromethane (50 mL). After stirring the reaction mixture at room temperature for 30 minutes, the excess lead(IV) oxide was filtered off and the solvent was removed under reduced pressure to yield **DPP2q**.

Yield 56.0 mg (60.0 µmol, 95 %). Melting point 279 °C. ^{1}H NMR (400 MHz, CD_2Cl_2): δ = 0.89 (t, 6 H, C*H*3, $^{3}J_{HH}$ = 7.1 Hz), 0.97 (t, 6 H, C*H*3, $^{3}J_{HH}$ = 7.5 Hz), 1.17 – 1.47 (m, 16 H, C*H*2), 1.34 (s, 18 H, (C*H*3)3), 1.35 (s, 18 H, C(C*H*3)3), 1.95 – 2.05 (m, 2 H, βC*H*), 3.99 – 4.15 (m, 4 H, αC*H*2), 7.30 (d, 2 H, aryl-C*H*, $^{4}J_{HH}$ = 2.5 Hz), 7.47 (d, 2 H, aryl-C*H*, $^{4}J_{HH}$ = 2.5 Hz), 7.62 (d, 2 H, heteroaryl-C*H*, $^{3}J_{HH}$ = 5.8 Hz), 9.38 (d, 2 H, heteroyral-C*H*, $^{3}J_{HH}$ = 5.8 Hz). ^{13}C NMR (100.6 MHz, CD_2Cl_2): δ = 10.8, 14.2, 23.5, 23.9, 28.7, 29.7, 29.8, 30.8, 35.9, 36.1, 40.1, 46.5, 126.0, 126.5, 128.3, 129.3, 130.6, 131.4, 133.0, 142.5, 147.6, 149.8, 155.1, 162.1, 185.9. HRMS (ESI-TOF, pos. mode, MeCN/ $CHCl_3$ 1/1): calculated for $C_{58}H_{78}N_2O_4S_2^+$: *m/z* 930.5398 $[M]^+$, found 930.5465. MS (MALDI-TOF, pos. mode, DCTB 3:1 in $CHCl_3$): calculated for $C_{58}H_{78}N_2O_4S_2^+$: *m/z* 930.540 $[M]^+$, found 930.420. UV/Vis (DCM, c = 1.00 · 10^{-5} M): λ_{max} [nm] (ε_{max} [L mol^{-1} cm^{-1}]) = 725 (67300), 782 (151800). IR (ATR): $\tilde{\nu}$ [cm^{-1}] = 2948 (s, $\nu_{-C-H,str}$), 2909 (s, $\nu_{-C-H,str}$), 2853 (s, $\nu_{-C-H,str}$), 1670 (vs, $\nu_{-C=O,str}$), 1584 (vs, $\nu_{-C=O,str}$).

2.4 Synthesis of DPP3

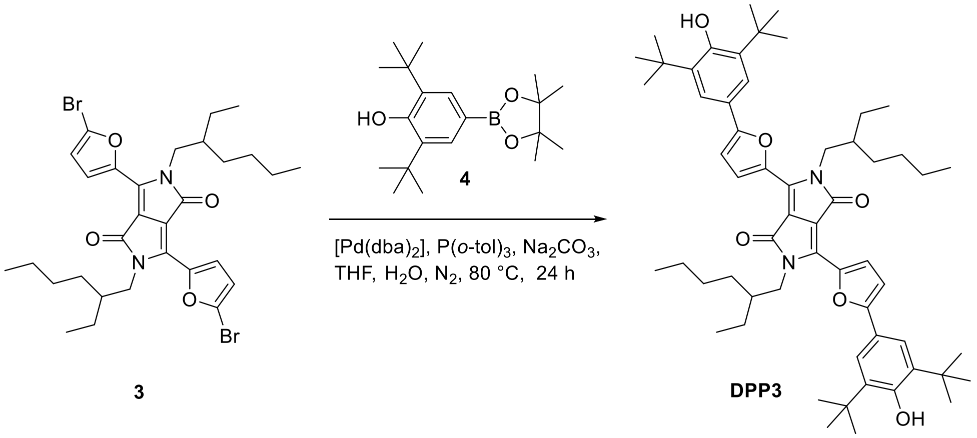

Scheme S5 Synthesis of **DPP3** by Suzuki-Miyaura cross-coupling.

Sodium carbonate (590 mg, 5.57 mmol, 18.1 equiv.), **3** (200 mg, 307 µmol, 1.00 equiv.), bis(dibenzylideneacetone)palladium(0) (11.3 mg, 19.6 µmol, 6.67 mol%), boronic ester **4** (258 mg, 770 µmol, 2.50 equiv.) and tri(*o*-tolyl)phosphine (12.0 mg, 39.0 µmol, 12.7 mol%) were dissolved in a degassed mixture of THF/H_2O (10 mL/ 2mL) under an atmosphere of nitrogen. After the mixture was heated to 80 °C for 24 h and cooled down to room temperature, water (30 mL) was added and the crude product was extracted with CH_2Cl_2 (3 x 20 mL). The combined organic layers were washed with hydrochloric acid (1 M), water, dried over $MgSO_4$ and the solvent was removed under reduced pressure. The crude product was purified by silica gel column chromatography (eluent: hexane/ CH_2Cl_2 (1:1)) to yield **DPP3** as a dark red solid.

Yield: 177 mg (197 µmol, 64 %). Melting point: 126–127 °C. ^{1}H NMR (400 MHz, CD_2Cl_2): δ = 0.78 (t, 6 H, CH_3, $^3J_{HH}$ = 7.0 Hz), 0.86 (t, 6 H, CH_3, $^3J_{HH}$ = 7.4 Hz), 1.14 – 1.41 (m, 16 H, CH_2), 1.50 (s, 36 H, C(CH_3)$_3$), 1.78 – 1.89 (m, 2 H, βC*H*), 4.12 – 4.24 (m, 4 H, αCH_2), 5.52 (s, 2 H, O*H*), 6.85 (d, 2 H, heteroaryl-C*H*, $^3J_{HH}$ = 3.8 Hz), 7.63 (s, 4 H, aryl-C*H*), 8.34 (d, 2 H, heteroaryl-C*H*, $^3J_{HH}$ = 3.8 Hz). ^{13}C NMR (101 MHz, CD_2Cl_2): δ = 10.9, 14.1, 23.4, 24.1, 29.0, 30.4, 30.8, 34.8, 39.9, 46.8, 106.8, 107.7, 121.6, 122.4, 122.6, 133.1, 137.2, 143.9, 155.4, 158.6, 161.4. HRMS (ESI-TOF, pos. mode, MeCN/ $CHCl_3$ 1/1): calculated for $C_{58}H_{80}N_2O_6^+$: *m/z* 900.6011 [M]$^+$, found 900.5951. MS (MALDI-TOF, pos. mode, DCTB 3:1 in $CHCl_3$): calculated for $C_{58}H_{80}N_2O_6^+$: *m/z* 900.601 [M]$^+$, found 900.445. UV/Vis (DCM, $c = 9.50 \cdot 10^{-6}$ M): λ_{max} [nm] (ε_{max} [M^{-1} cm^{-1}]) = 561 (49600), 607 (82500). IR (ATR): $\tilde{\nu}$ [cm^{-1}] = 3625 (s, $\nu_{O\text{-}H,str}$), 2950 (s, $\nu_{C\text{-}H,str}$), 2918 (s, $\nu_{C\text{-}H,str}$), 2855 (s, $\nu_{C\text{-}H,str}$), 1654 (vs, $\nu_{C=O,str}$). Fluorescence (DCM, $\lambda_{ex.}$ = 540 nm): λ_{max} = 624 nm (ϕ = 0.48 ± 0.08, standard: Oxazine 1).

2.5 Synthesis of DPP3q

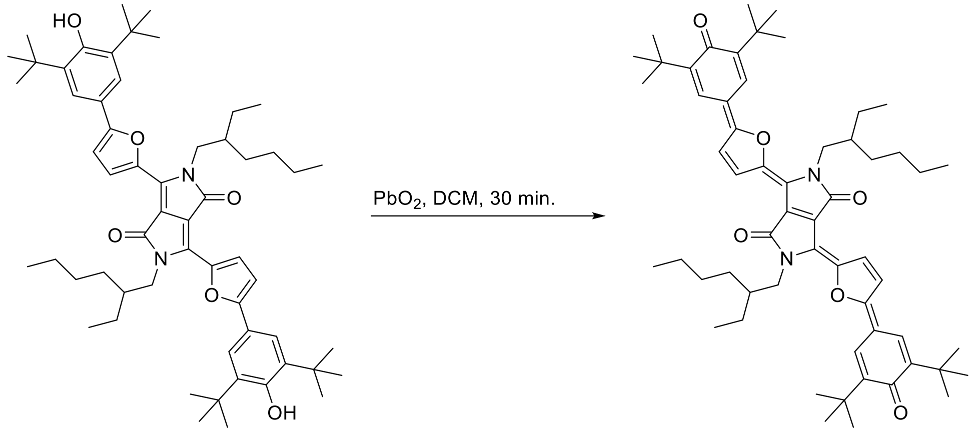

DPP3 **DPP3q**

Scheme S6 Synthesis of **DPP3q** by oxidation of **DPP3** with lead(IV)oxide.

Lead(IV) oxide (210 mg, 878 µmol, 14.0 equiv.) and **DPP3** (57.0 mg, 63.0 µmol, 1.00 equiv.) were suspended in dichloromethane (50 mL). After stirring the reaction mixture at room temperature for 30 minutes the excess lead(IV) oxide was filtered off and the solvent was removed under reduced pressure to yield **DPP3q**.

Yield: 56.0 mg (62.3 µmol, 99 %). Melting point: 274 °C. ^{1}H NMR (400 MHz, CD_2Cl_2): δ = 0.84 (t, 6 H, C*H*$_3$, $^{3}J_{HH}$ = 7.0 Hz), 0.90 (t, 6 H, C*H*$_3$, $^{3}J_{HH}$ = 7.4 Hz), 1.20 – 1.53 (m, 16 H, C*H*$_2$), 1.35 (s, 18 H, C(C*H*$_3$)$_3$), 1.37 (s, 18 H, C(C*H*$_3$)$_3$), 1.84 – 1.95 (m, 2 H, βC*H*), 4.16 (d, 4 H, αC*H*$_2$, $^{3}J_{HH}$ = 7.7 Hz), 7.32 (d, 2 H, aryl-C*H*, $^{4}J_{HH}$ = 2.4 Hz), 7.40 (d, 2 H, heteroaryl-C*H*, $^{3}J_{HH}$ = 5.5 Hz), 7.60 (d, 2 H, aryl-C*H*, $^{4}J_{HH}$ = 2.4 Hz), 8.85 (d, 2 H, heteroaryl-C*H*, $^{3}J_{HH}$ = 5.5 Hz). ^{13}C NMR (100.6 MHz, CD_2Cl_2): δ = 10.9, 14.2, 23.4, 24.1, 28.9, 29.7, 29.9, 30.7, 35.9, 36.2, 39.6, 47.7, 115.5, 119.9, 123.6, 124.5, 126.9, 130.6, 133.6, 146.8, 148.4, 149.6, 161.3, 162.9, 185.8. HRMS (ESI-TOF, pos. mode, MeCN/ $CHCl_3$ 1/1): calculated for $C_{58}H_{78}N_2O_6^+$: *m/z* 898.5854 $[M]^+$, found 898.5900. MS (MALDI-TOF, pos. mode, DCTB 3:1 in $CHCl_3$): calculated for $C_{58}H_{78}N_2O_6^+$: *m/z* 898.585 $[M]^+$, found 898.457. UV/Vis (DCM, c = 1.00 · 10^{-5} M): λ_{max} [nm] (ε_{max} [M^{-1} cm^{-1}]) = 695 (92200), 751 (172500). IR (ATR): $\tilde{\nu}$ [cm^{-1}] = 3105 (m, $\nu_{C=O,str\_ot}$), 2953 (s, $\nu_{C-H,str}$), 2918 (s, $\nu_{C-H,str}$), 2860 (s, $\nu_{C-H,str}$), 1660 (vs, $\nu_{C=O,str}$), 1578 (vs, $\nu_{C=O,str}$).

2.6 Preparation of DPP1•• sample

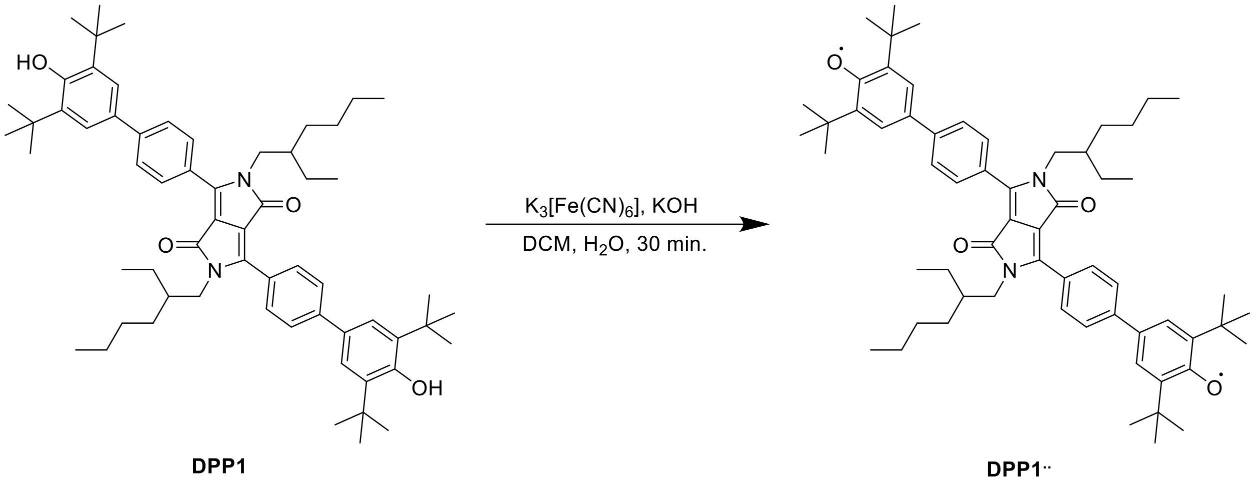

Scheme S7 Synthesis of biradical **DPP1••** by oxidation of **DPP1** with potassium ferricyanide(III).

DPP1 (3.00 mg, 3.26 µmol, 1.00 equiv.), potassium ferricyanide(III) (127 mg, 386 µmol, 118 equiv.) and potassium hydroxide (200 mg, 3.56 mmol, 1092 equiv.) were dissolved in a biphasic mixture of CH_2Cl_2/ H_2O (1.5 mL/ 2.0 mL) and vigorously stirred for 30 min. The organic layer was subsequently washed with water and dried over $MgSO_4$ to give a solution of **DPP1••** in CH_2Cl_2 (c = 2.17 mM).

HRMS (ESI-TOF, pos. mode, CH_2Cl_2/MeCN 1/1): calculated for $C_{62}H_{82}N_2O_4^+$: *m/z* 918.6269 $[M]^+$, found 918.6250. UV/Vis (DCM, $c = 9.80 \cdot 10^{-6}$ M): λ_{max} [nm] (ε_{max} [L mol^{-1} cm^{-1}]) = 269 (35000), 356 (20400), 486 (30100).

3 Mass spectrometry

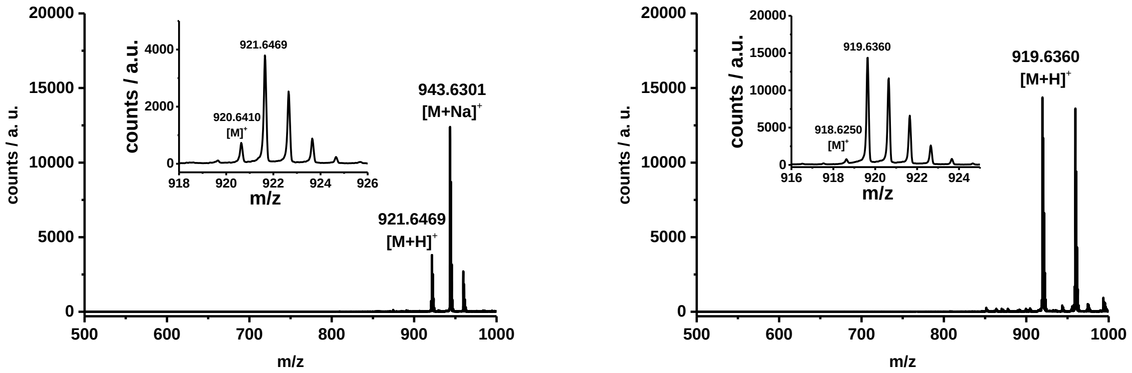

Fig. S1 ESI-TOF high resolution mass spectra of **DPP1** (left) and **DPP1••** (right). Inset: Isotopic distribution.

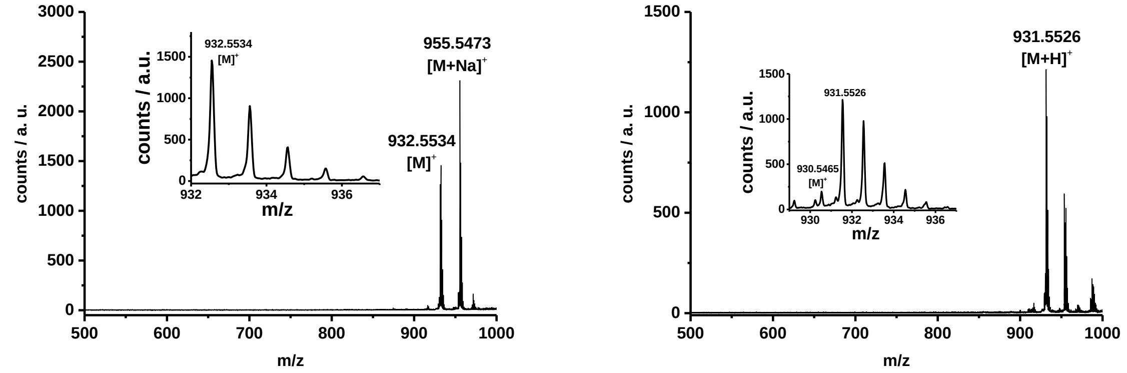

Fig. S2 ESI-TOF high resolution mass spectra of **DPP2** (left) and **DPP2q** (right). Inset: Isotopic distribution.

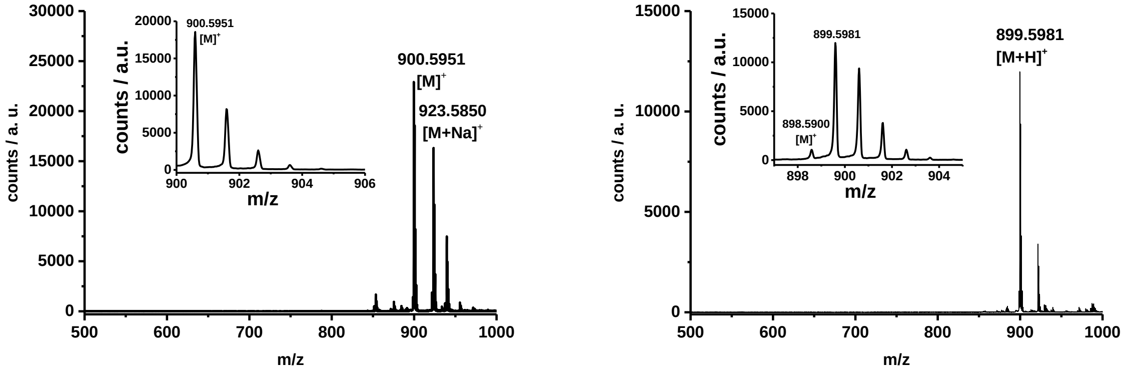

Fig. S3 ESI-TOF high resolution mass spectra of **DPP3** (left) and **DPP3q** (right). Inset: Isotopic distribution.

4 Single crystal X-ray diffraction

Single crystals of **DPP3q** were obtained by slow diffusion of methanol into a solution of **DPP3q** in $CHCl_3$. Two almost identical structures were found with bond length deviations ≤ 0.5 pm.

*Crystal data for **DPP3q*** ($C_{58}H_{78}N_2O_6$ • 2 (CH_4O)): *M*r = 963.31, 0.489x0.361x0.327 mm^3, monoclinic space group $P2_1/n$, a = 15.3311(16) Å, α = 90°, b = 23.844(3) Å, β = 95.515(3)°, c = 15.4674(16) Å, γ = 90°, V = 5627.9(10) $Å^3$, Z = 4, $\rho(calcd)$ = 1.137 g·cm^{-3}, μ = 0.074 mm^{-1}, $F_{(000)}$ = 2096, $GooF(F^2)$ = 1.037, R_1 = 0.0591, wR^2 = 0.1454 for I>2σ(I), R_1 = 0.0860, wR^2 = 0.1599 for all data, 11128 unique reflections [θ ≤ 26.158°] with a completeness of 98.9 % and 718 parameters, 72 restraints.

Single crystals of **DPP1** were obtained slow diffusion of methanol into a solution of **DPP1** in $CHCl_3$.

*Crystal data for **DPP1*** ($C_{62}H_{84}N_2O_4$): *M*r = 921.31, 0.281x0.133x0.114 mm^3, triclinic space group $P\bar{1}$, a = 9.8347(4) Å, α = 118.5760(10)°, b = 12.6226(5) Å, β = 101.4230(10)°, c = 12.8064(6) Å, γ = 94.6720(10)°, V = 1339.49(10) $Å^3$, Z = 1, $\rho(calcd)$ = 1.142 g·cm^{-3}, μ = 0.537 mm^{-1}, $F_{(000)}$ = 502, $GooF(F^2)$ = 1.041, R_1 = 0.0464, wR^2 = 0.1195 for I>2σ(I), R_1 = 0.0492, wR^2 = 0.1220 for all data, 5260 unique reflections [θ ≤ 72.294°] with a completeness of 99.3 % and 426 parameters, 61 restraints.

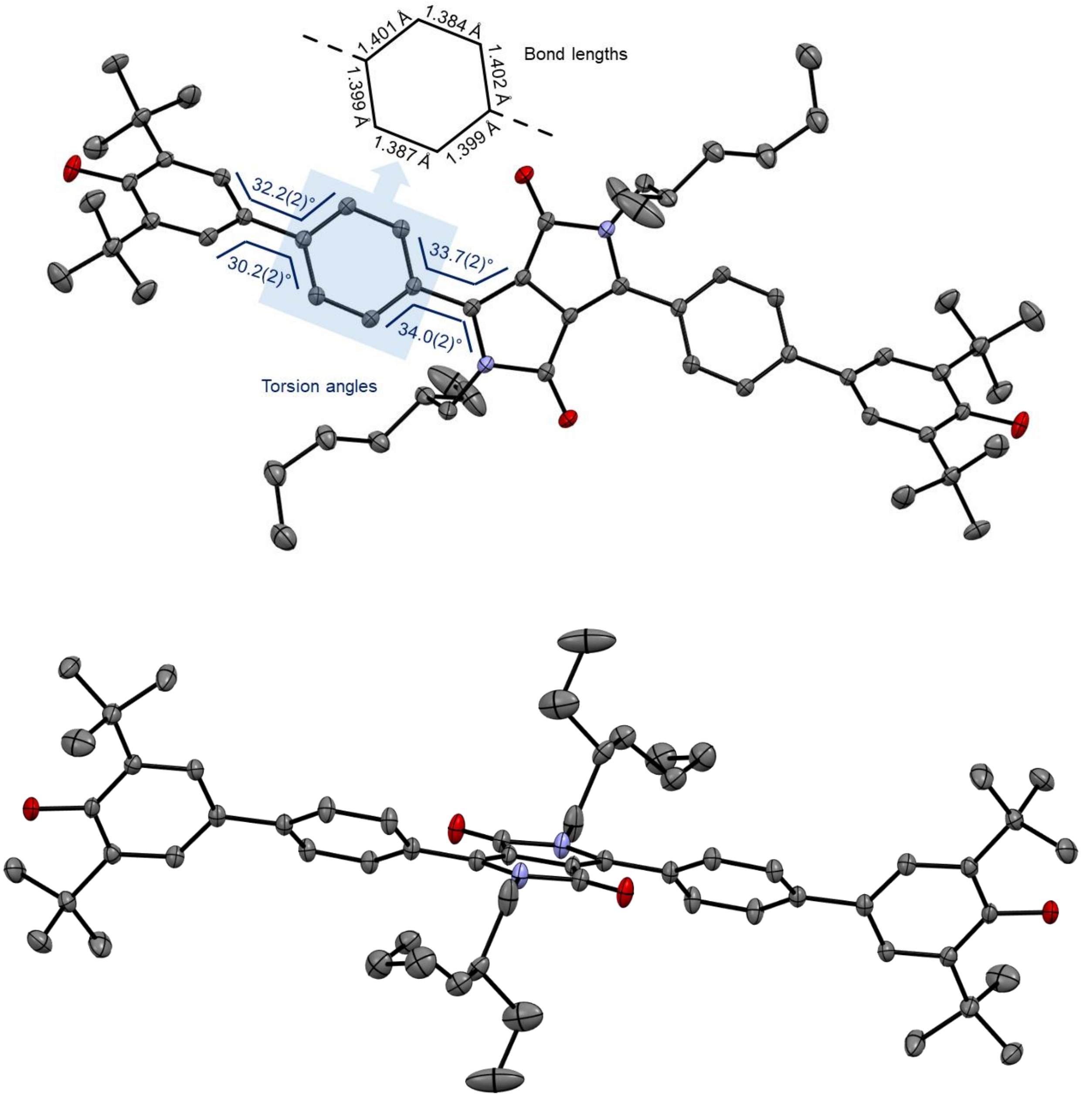

Fig. S4 Solid state molecular structure of **DPP1** in topview (top) and sideview (bottom) determined by single crystal X-ray diffraction (ellipsoids set to 50 % probability, carbon gray, nitrogen blue, oxygen red). Hydrogen omitted for clarity.

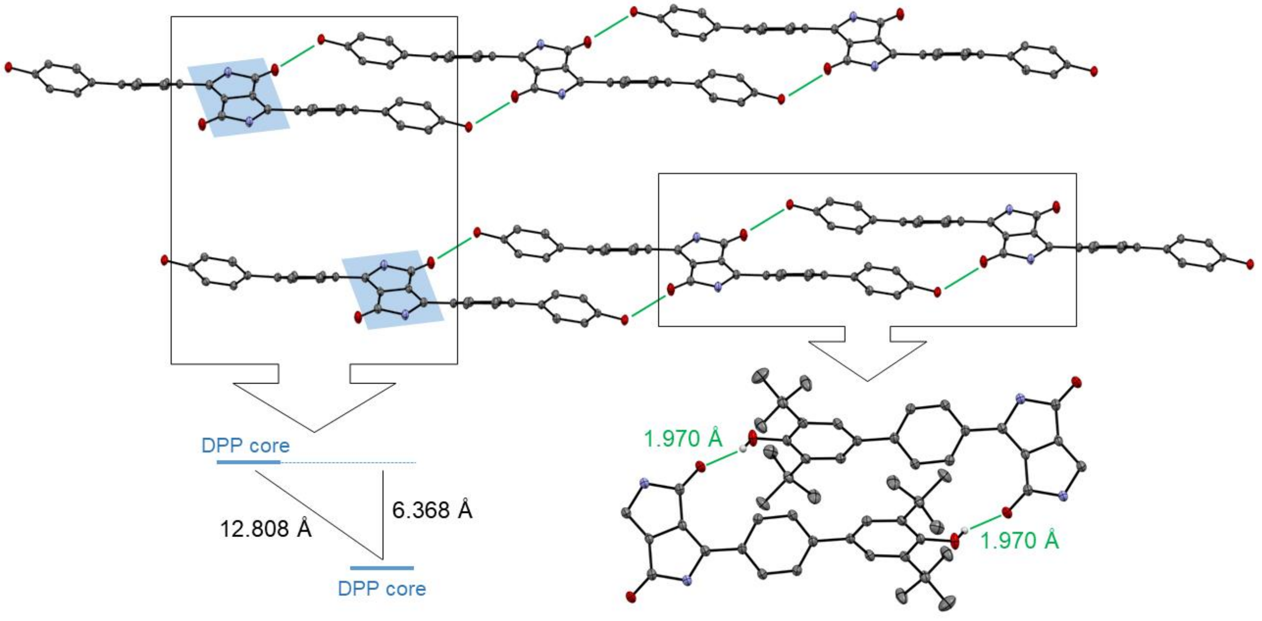

Fig. S5 Solid state molecular structure of **DPP1** determined by X-ray diffraction (ellipsoids set to 50 % probability) with intermolecular hydrogen bonding (hydrogen bond = green line, carbon gray, nitrogen blue, oxygen red, hydrogen white). Alkyl chains and hydrogen atoms partially omitted for clarity.

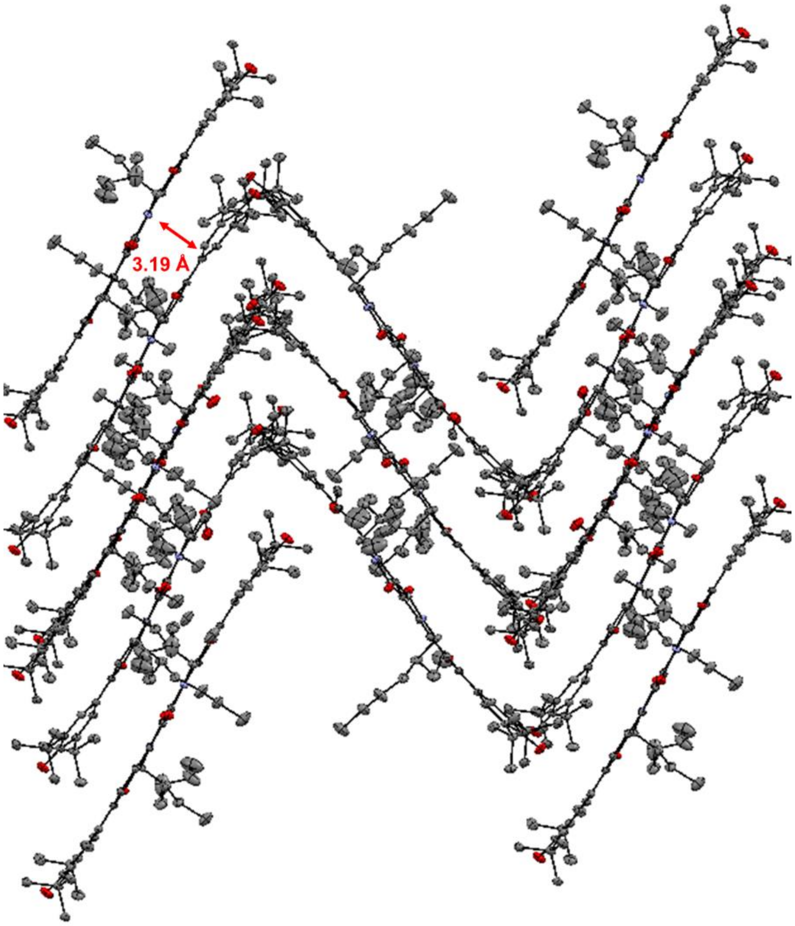

Fig. S6 Solid state molecular packing of **DPP3q** in herringbone type arrangement determined by single crystal X-ray diffraction (ellipsoids set to 50 % probability, carbon gray, nitrogen blue, oxygen red). Solvent molecules (methanol) and hydrogen atoms omitted for clarity.

5 UV/vis/NIR spectroscopy and spectroelectrochemistry

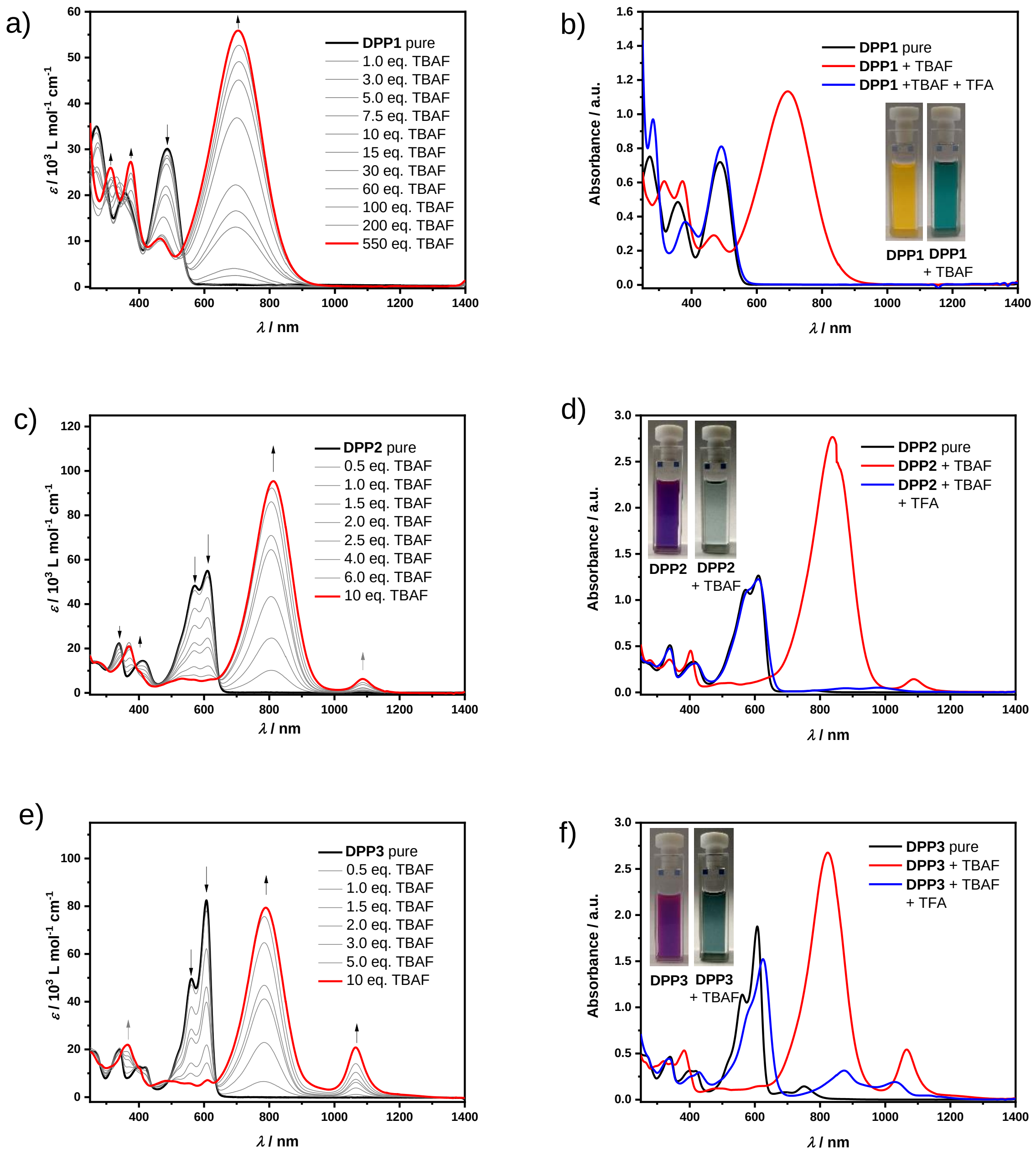

Fig. S7 UV/vis/NIR absorption spectral changes of (a) **DPP1** (c = 1.01 · 10^{-5} M in CH_2Cl_2), (c) **DPP2** (c = 9.30 · 10^{-6} M in CH_2Cl_2) and (e) **DPP3** (c = 9.60 · 10^{-6} M in CH_2Cl_2) upon titration with $^{n}Bu_4NF$ (TBAF) solutions (c = 7.50 · 10^{-2} M in CH_2Cl_2) to form dianions **DPP1^{2-}**, **DPP2^{2-}** and **DPP3^{2-}**, respectively. UV/vis/NIR absorption spectra of (b) **DPP1^{2-}** (red, c = 2.44 · 10^{-5} M in CH_2Cl_2), (d) **DPP2^{2-}** (red, c = 2.19 · 10^{-5} M in CH_2Cl_2) and (f) **DPP3^{2-}** (red, c = 2.26 · 10^{-5} M in CH_2Cl_2) freshly prepared by adding 100 eq. $^{n}Bu_4NF$ to a solution of the respective neutral dye (black line) and after addition of 100 eq. trifluoroacetic acid (TFA) (blue line) in CH_2Cl_2. Inset: Photographs of respective cuvettes.

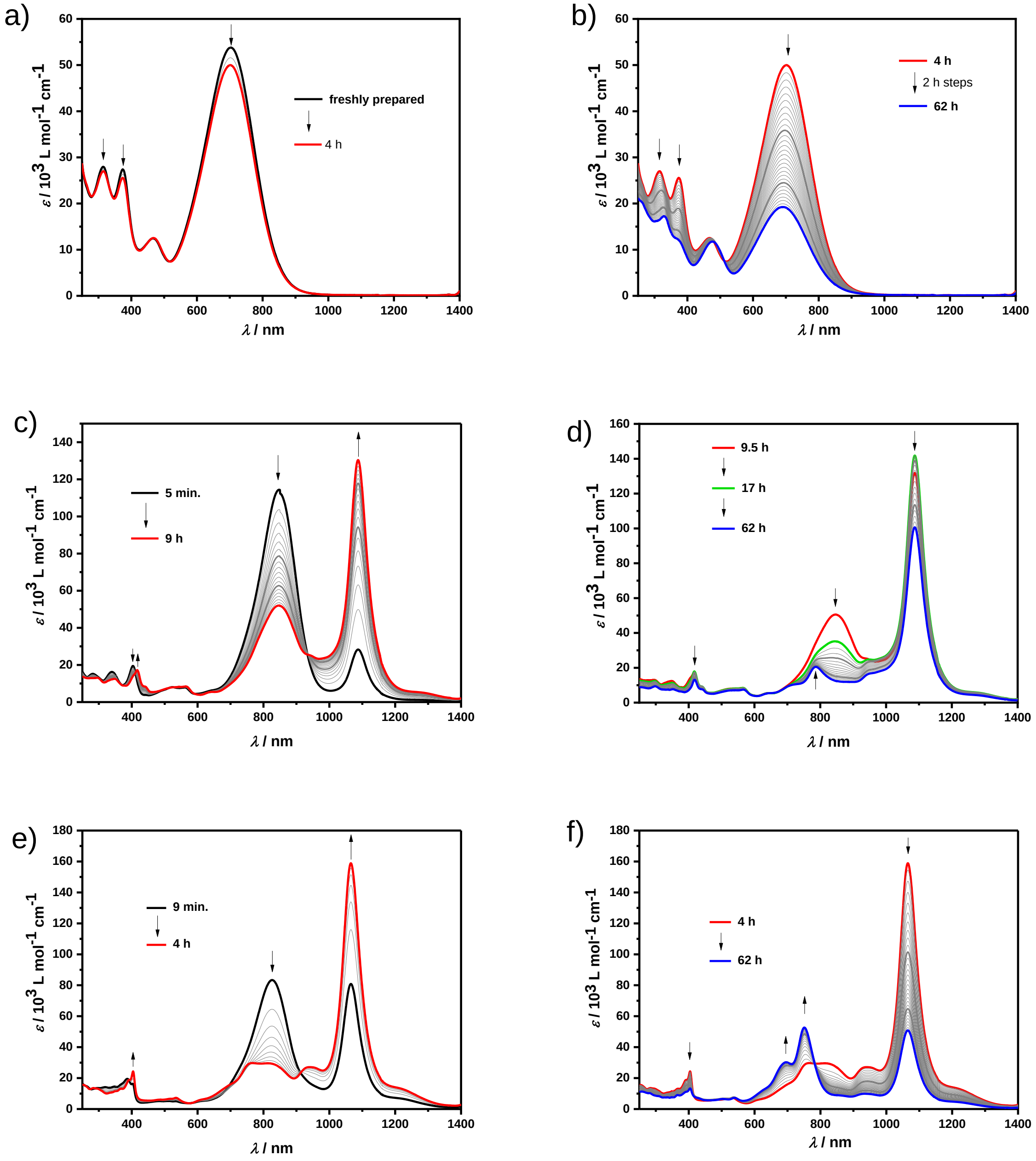

Fig. S8 Time-dependent UV/vis/NIR absorption spectral changes of (a, b) **DPP1**$^{2-}$, (c, d) **DPP2**$^{2-}$ and (e, f) **DPP3**$^{2-}$ under ambient conditions (c = 1.00 · 10^{-5} M in CH_2Cl_2, counter ion: $^{n}Bu_4N^+$). Arrows indicate spectral changes over time.

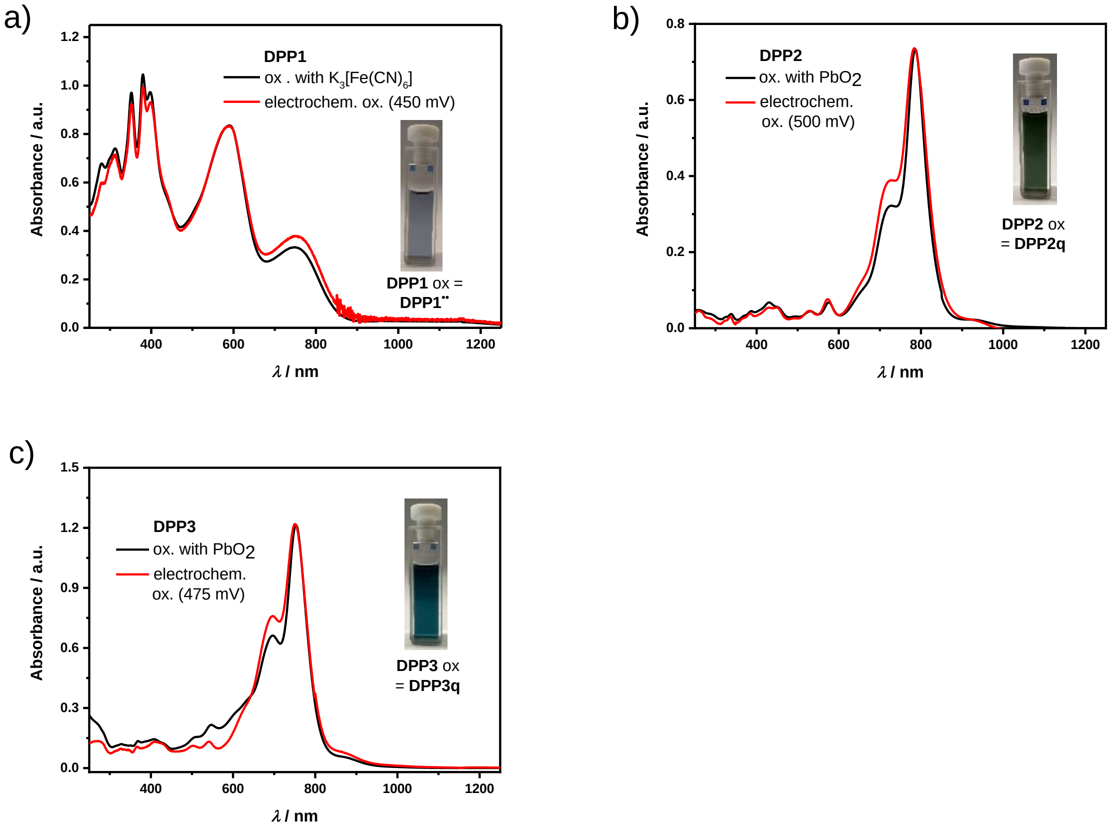

Fig. S9 Normalized UV/vis/NIR absorption spectra of (a) **DPP1•+**, (b) **DPP2q** and (c) **DPP3q** obtained by chemical oxidation with $K_3[Fe(CN)_6]$ or PbO_2 (black solid line, *c* ≈ 10 µM in CH_2Cl_2) and electrochemical oxidation (red solid line, *c* ≈ 3 mM in CH_2Cl_2) of the respective precursors **DPP1**, **DPP2** and **DPP3**. Inset: Photographs of respective cuvettes.

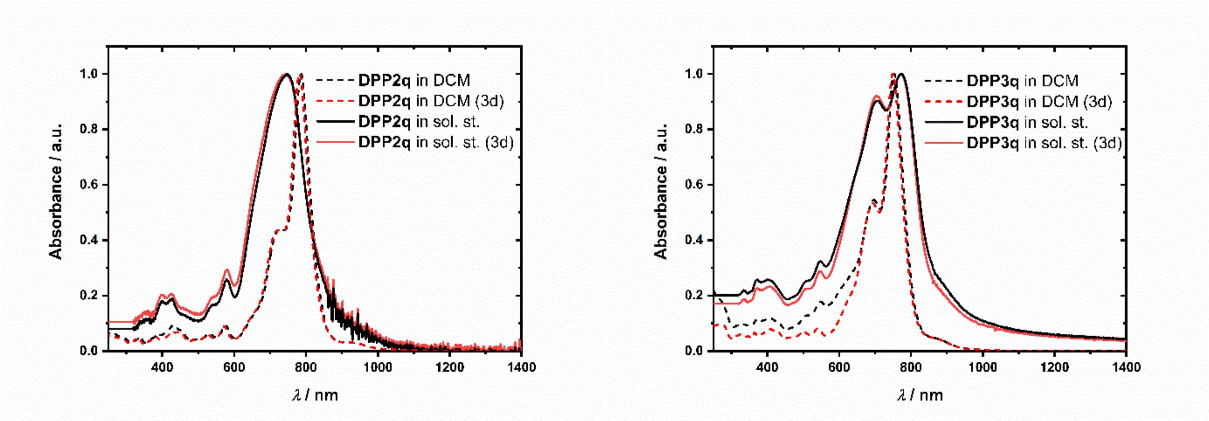

Fig. S10 Normalized UV/vis/NIR absorption spectra of **DPP2q** (left) and **DPP3q** (right) in solution (black dashed line, *c* ≈ 10 µM in CH_2Cl_2) and in the solid state (*c* ≈ 2 mM, 25 µL, ambient temperature, *rpm* = 3000, *t* = 30 s) as spincoated (red solid line) and after 3 days (black solid line) under ambient conditions.

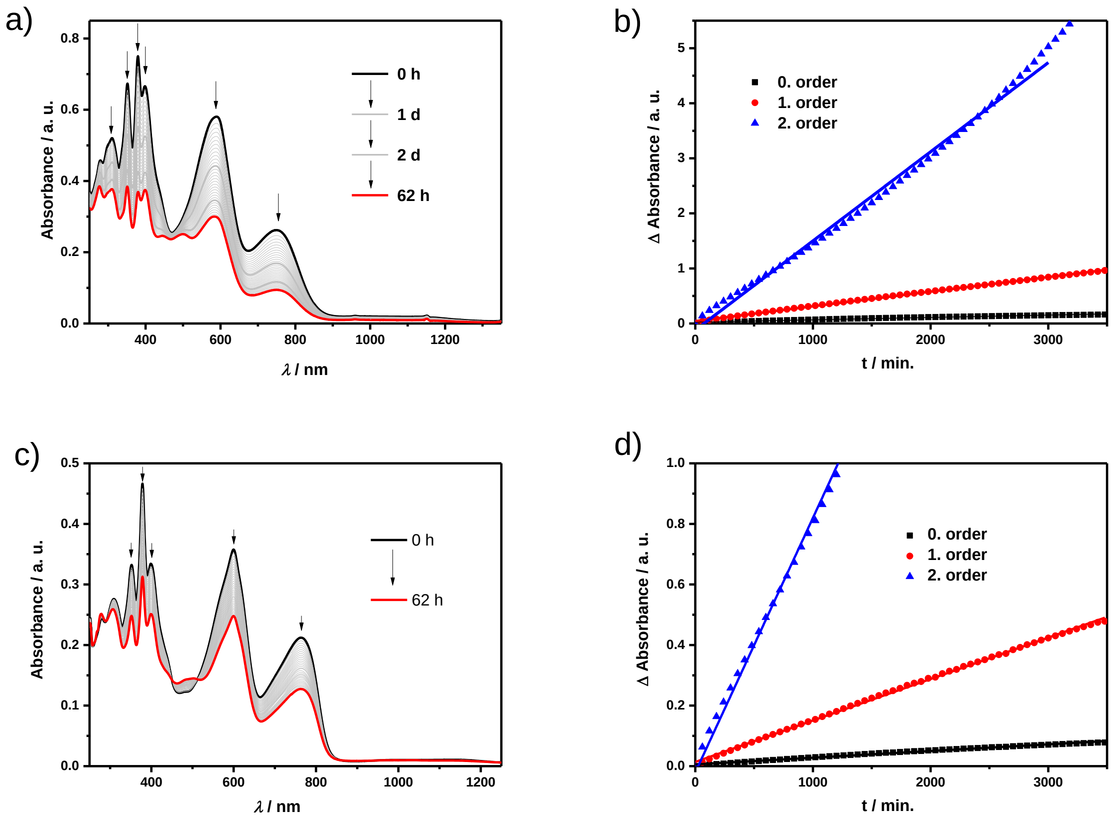

Fig. S11 Time-dependent UV/vis/NIR absorption spectral changes of **DPP1**$^{\bullet\bullet}$ (c = $1.00 \cdot 10^{-5}$ M, ambient conditions) in (a) CH_2Cl_2 and (c) CCl_4. Absorption spectral changes of **DPP1**$^{\bullet\bullet}$ (in CCl_4) at 750 nm in (b) CH_2Cl_2 and (d) CCl_4 plotted versus time using zero-, first- and second-order rate equations. Arrows indicate spectral changes over time. Triangles, dots and circles represent the experimental results and the solid lines correspond to the respective fit.

Spectral changes observed were fitted with zero, first and second order kinetic models. As measurement data can be described with all three models with good accuracy, the minimum lifetime was chosen to be the lowest value obtained.

Table S1 Kinetic evaluation of the time-dependent UV/vis/NIR absorption spectral changes of **DPP1••** in CH_2Cl_2 (c = 1.00 · 10^{-5} M, ambient conditions) upon decomposition under ambient conditions. $[A]_0$ = initial absorption, $[A]_t$ = time dependent absorption.

rate law	equations		linear regression	rate constant *k*	half-life $\tau_{\frac{1}{2}}$ [h]	Pearson R^2
	rate equation	half-life				
**zero order**	$[A]_0 - [A]_t = kt$	$\tau_{\frac{1}{2}} = \frac{A_0}{2k}$	y = 0.0000430284x + 0.02372	2.58 · 10^{-4} $\frac{1}{h}$	51	0.97590
**first order**	$ln\frac{[A]_0}{[A]_t} = kt$	$\tau_{\frac{1}{2}} = \frac{ln2}{k}$	y = 0.000266114x + 0.04563	1.60 · 10^{-2} $\frac{1}{h}$	43	0.99894
**second order**	$\frac{1}{[A]_t} - \frac{1}{[A]_0} = kt$	$\tau_{\frac{1}{2}} = \frac{1}{kA_0}$	y = 0.00162x - 0.11879	9.72 · 10^{-2} $\frac{1}{h}$	**39**	0.99410

Table S2 Kinetic evaluation of the time-dependent UV/vis/NIR absorption spectral changes of **DPP1••** in CCl_4 (c = 1.00 · 10^{-5} M, ambient conditions) upon decomposition under ambient conditions. $[A]_0$ = initial absorption, $[A]_t$ = time dependent absorption.

rate law	equations		linear regression	rate constant *k*	half-life $\tau_{\frac{1}{2}}$ [h]	Pearson R^2
	rate equation	half-life				
**zero order**	$[A]_0 - [A]_t = kt$	$\tau_{\frac{1}{2}} = \frac{A_0}{2k}$	y = 0.0000220889x + 0.00586	1.33 · 10^{-3} $\frac{1}{h}$	**78**	0.99140
**first order**	$ln\frac{[A]_0}{[A]_t} = kt$	$\tau_{\frac{1}{2}} = \frac{ln2}{k}$	y = 0.000136849x + 0.01397	8.21 · 10^{-3} $\frac{1}{h}$	84	0.99901
**second order**	$\frac{1}{[A]_t} - \frac{1}{[A]_0} = kt$	$\tau_{\frac{1}{2}} = \frac{1}{kA_0}$	y = 0.000840573x - 0.02077	50.4 · 10^{-3} $\frac{1}{h}$	96	0.99906

Table S3 Absorption maxima of neutral diketopyrrolopyrrole derivatives **DPP1, DPP2** and **DPP3**, dianions **DPP1**$^{2-}$, **DPP2**$^{2-}$ and **DPP3**$^{2-}$, mixed valent intermediates **MV-DPP2** and **MV-DPP3**, biradical **DPP1**$^{\bullet\bullet}$ and quinones **DPP2q** and **DPP3q** in dichloromethane solution ($c \approx 10$ µM, ambient conditions).

Compound	λ_{max} [nm]	ε [M^{-1} cm^{-1}] (λ)
**DPP1**	269, 356, 486	30100 (486 nm)
**DPP1**$^{2-}$	312, 374, 704	55900 (704 nm)
**DPP1**$^{\bullet\bullet}$	352, 380, 399, 588, 749	18500 (749 nm)
**DPP2**	572, 610	55000 (610 nm)
**DPP2**$^{2-}$	813	95400 (813 nm)
**MV-DPP2**	1083	152600 (1083 nm)
**DPP2q**	725, 782	151800 (782 nm)
**DPP3**	561, 607	82500 (607 nm)
**DPP3**$^{2-}$	789	79400 (789 nm)
**MV-DPP3**	1063	190800 (1063 nm)
**DPP3q**	695, 751	172500 (751 nm)

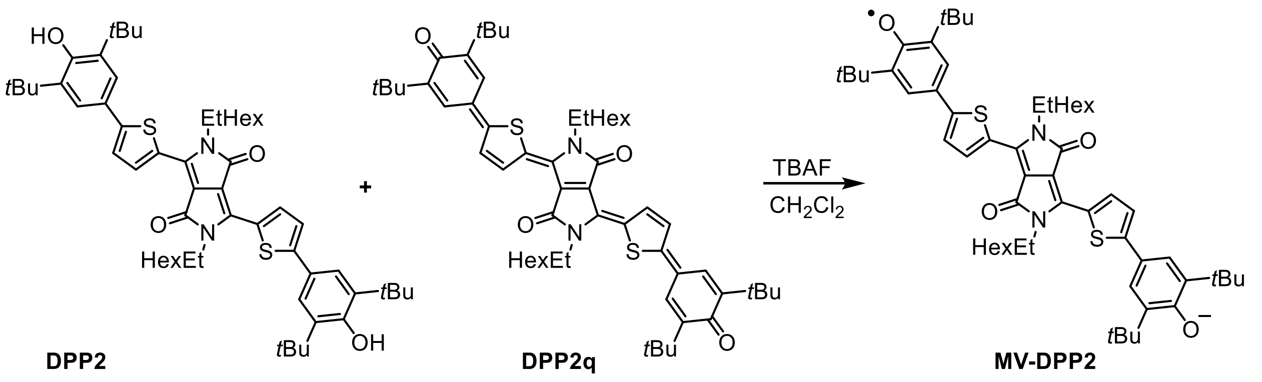

Scheme S8 Generation of **MV-DPP2** by comproportionation of **DPP2q** and *in situ* generated **DPP2**$^{2-}$.

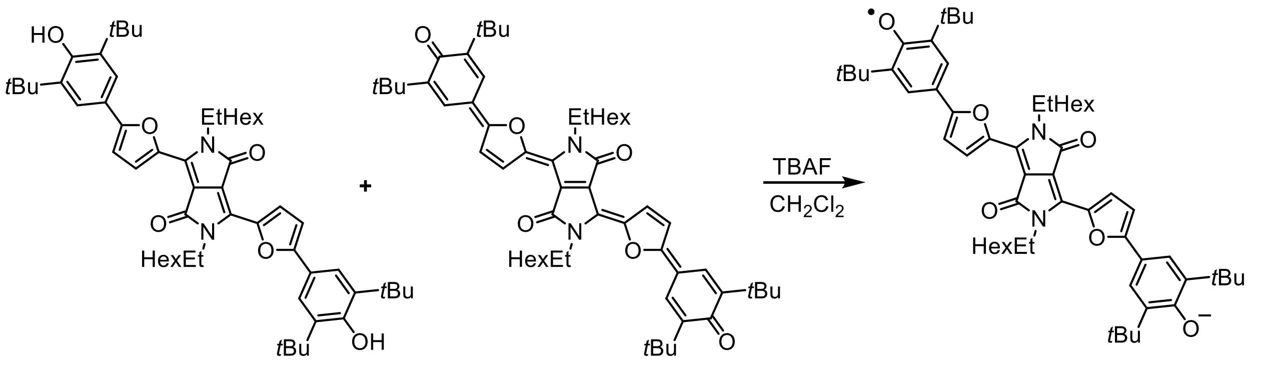

DPP3 **DPP3q** **MV-DPP3**

Scheme S9 Generation of **MV-DPP3** by comproportionation of **DPP3q** and *in situ* generated **DPP3^{2-}**.

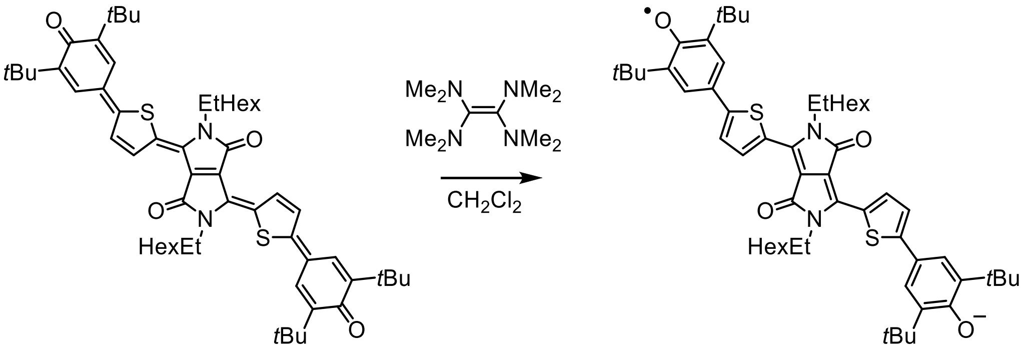

DPP2q **MV-DPP2**

Scheme S10 Generation of **MV-DPP2** by single electron transfer of TDMAE to **DPP2q**.

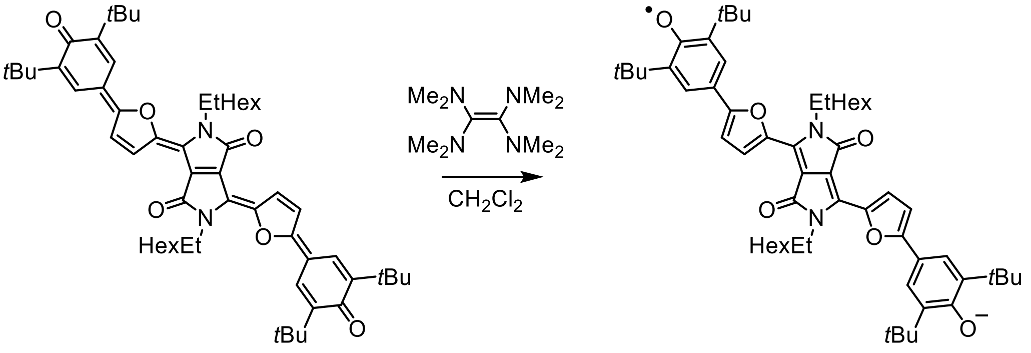

DPP3q **MV-DPP3**

Scheme S11 Generation of **MV-DPP3** by single electron transfer of TDMAE to **DPP3q**.

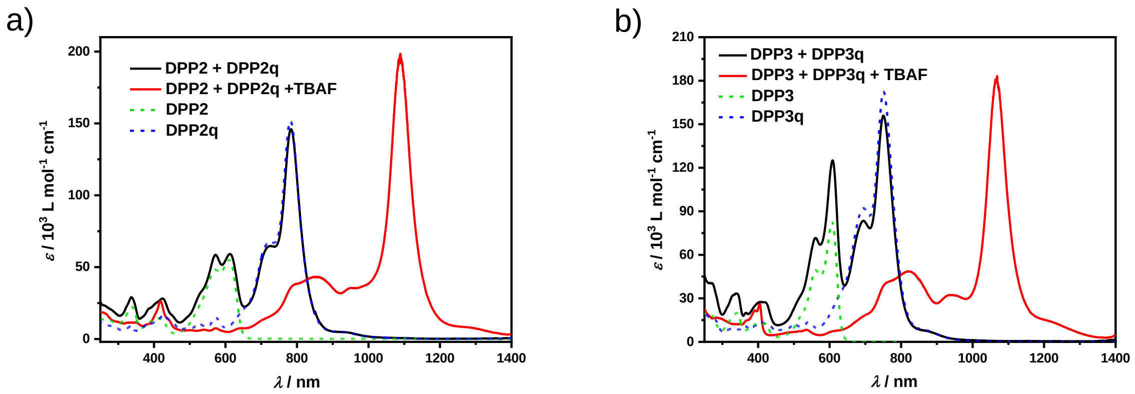

Fig. S12 UV/vis/NIR absorption spectra of equimolar mixtures of (a) **DPP2** and **DPP2q**, and (b) **DPP3** and **DPP3q** (each c = 1.00 · 10^{-5} M in CH_2Cl_2) before (black solid line) and after (red solid line, concentration corrected: Absorbance/2) addition of TBAF.

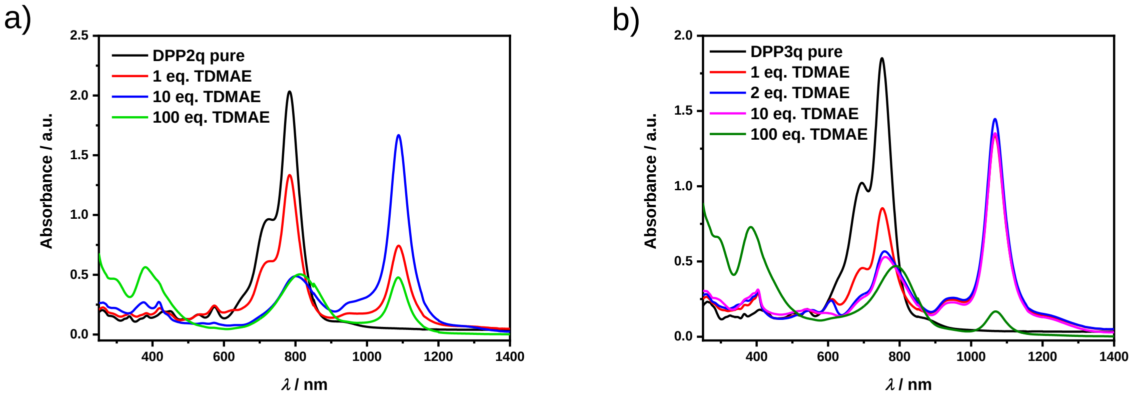

Fig. S13 UV/vis/NIR absorption spectral changes of (a) **DPP2q**, and (b) **DPP3q** (both c = 1.00 · 10^{-5} M in CH_2Cl_2) upon addition of tetrakis(dimethylamino)ethylene (TDMAE) solutions (c = 2.00 · 10^{-3} M in CH_2Cl_2) to form mixed valent **MV-DPP2** and **MV-DPP3**, respectively.

6 NMR spectroscopy

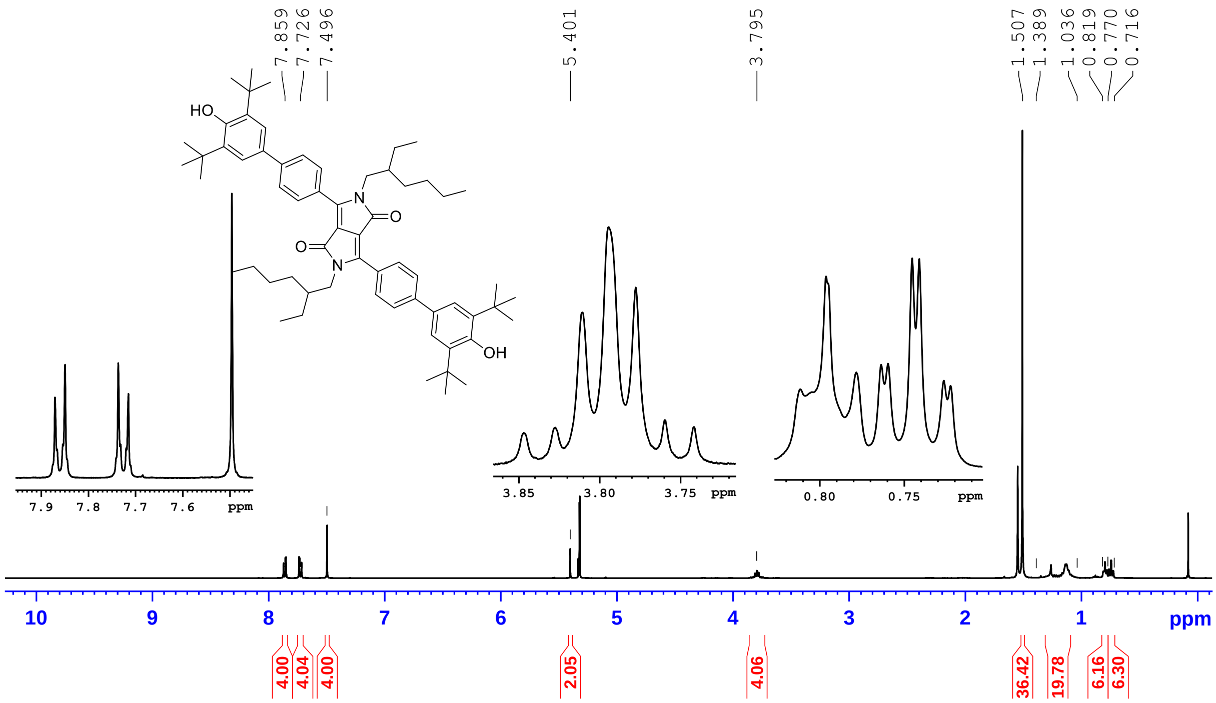

Fig. S14 ^{1}H NMR spectrum (400 MHz) of **DPP1** in CD_2Cl_2 at 295 K.

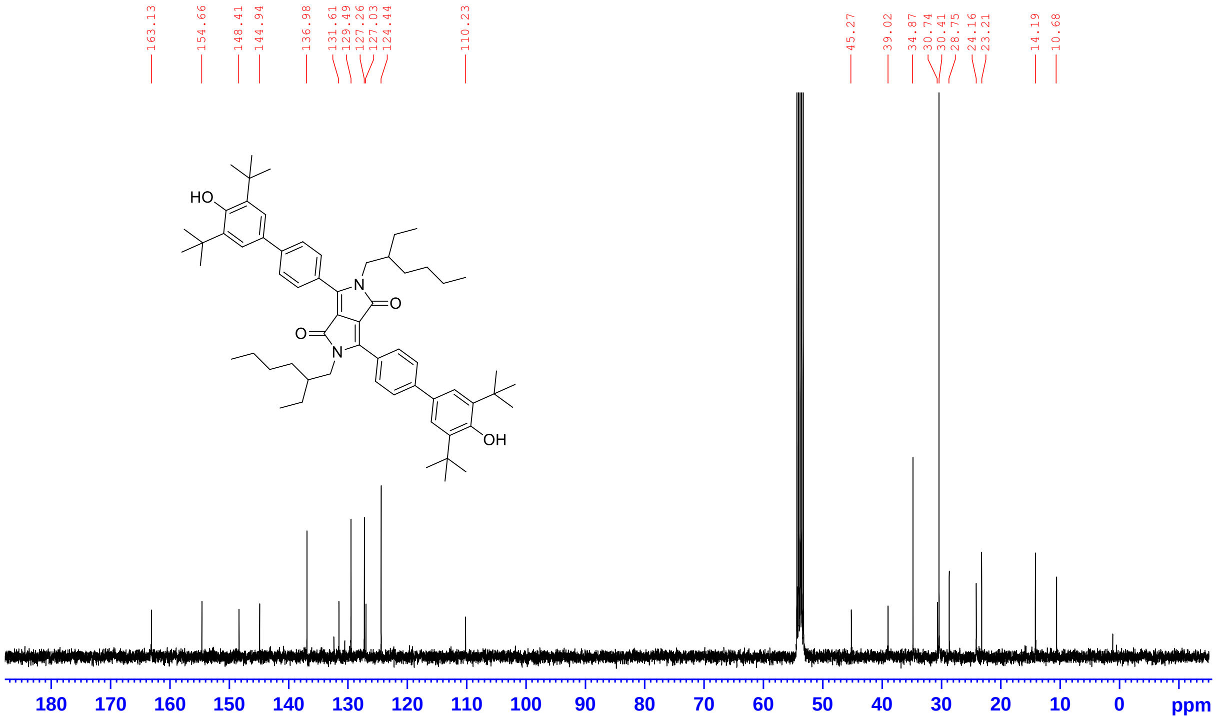

Fig. S15 ^{13}C NMR spectrum (101 MHz) of **DPP1** in CD_2Cl_2 at 295 K.

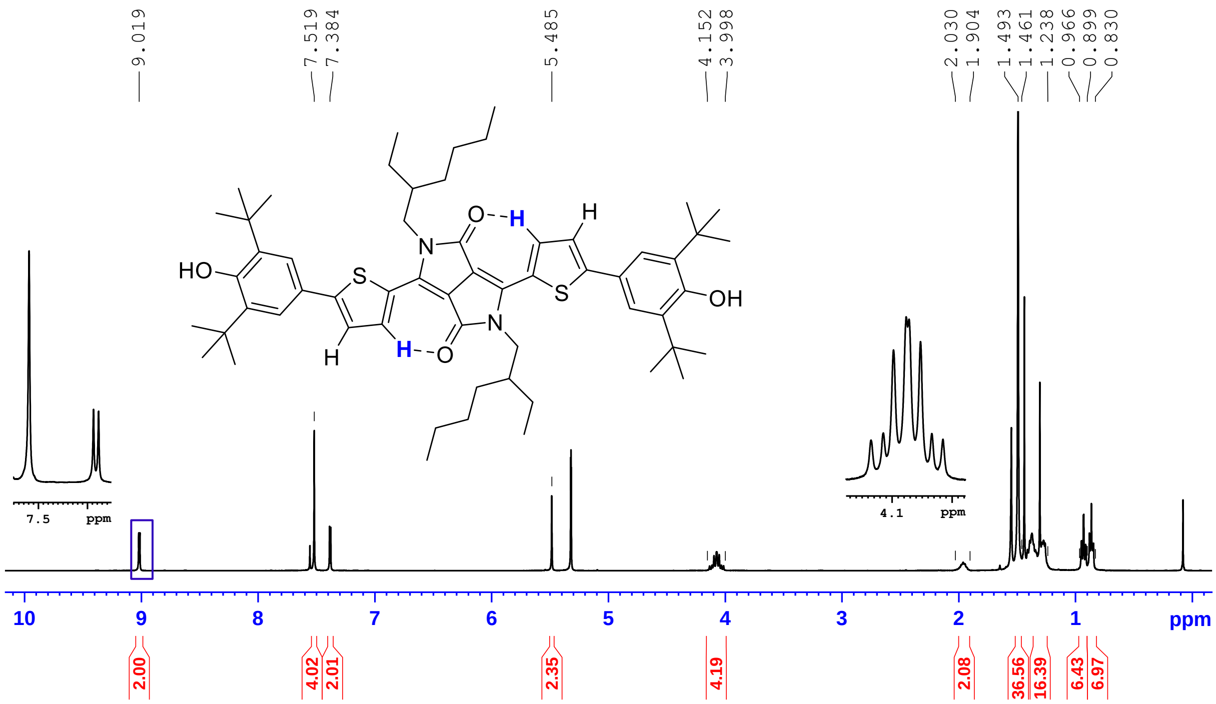

Fig. S16 ^{1}H NMR spectrum (400 MHz) of **DPP2** in CD_2Cl_2 at 295 K. Hydrogen bonded proton highlighted in blue.

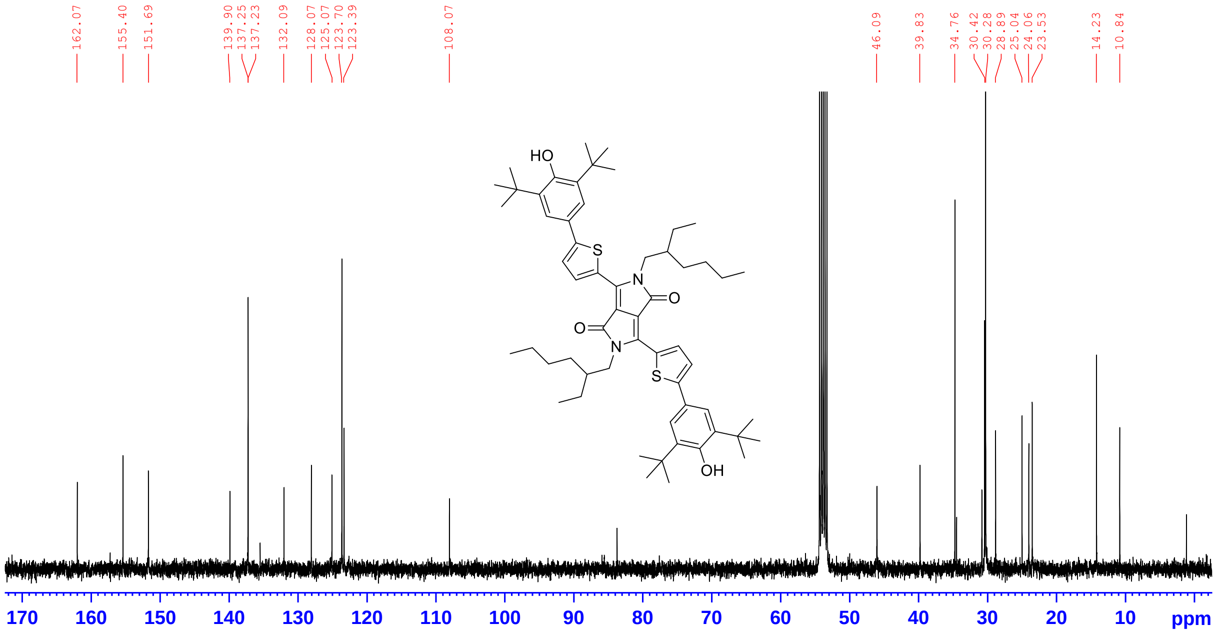

Fig. S17 ^{13}C NMR spectrum (101 MHz) of **DPP2** in CD_2Cl_2 at 295 K.

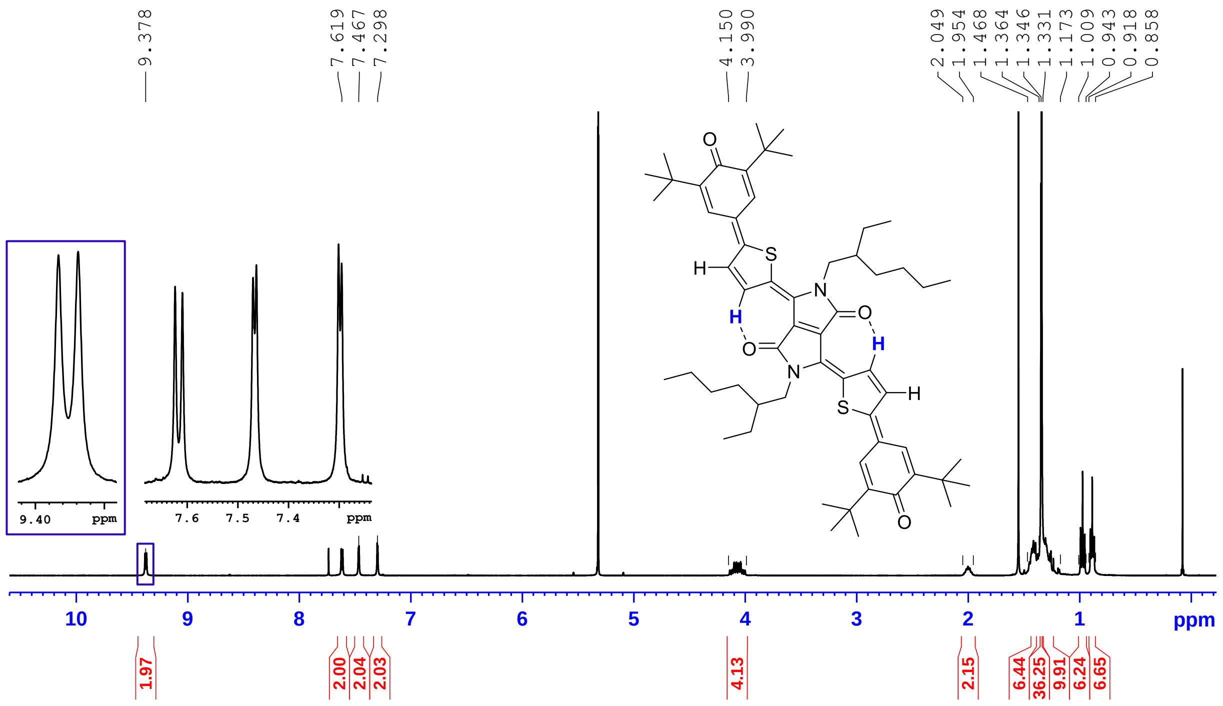

Fig. S18 ^{1}H NMR spectrum (400 MHz) of **DPP2q** in CD_2Cl_2 at 295 K. Hydrogen bonded proton highlighted in blue.

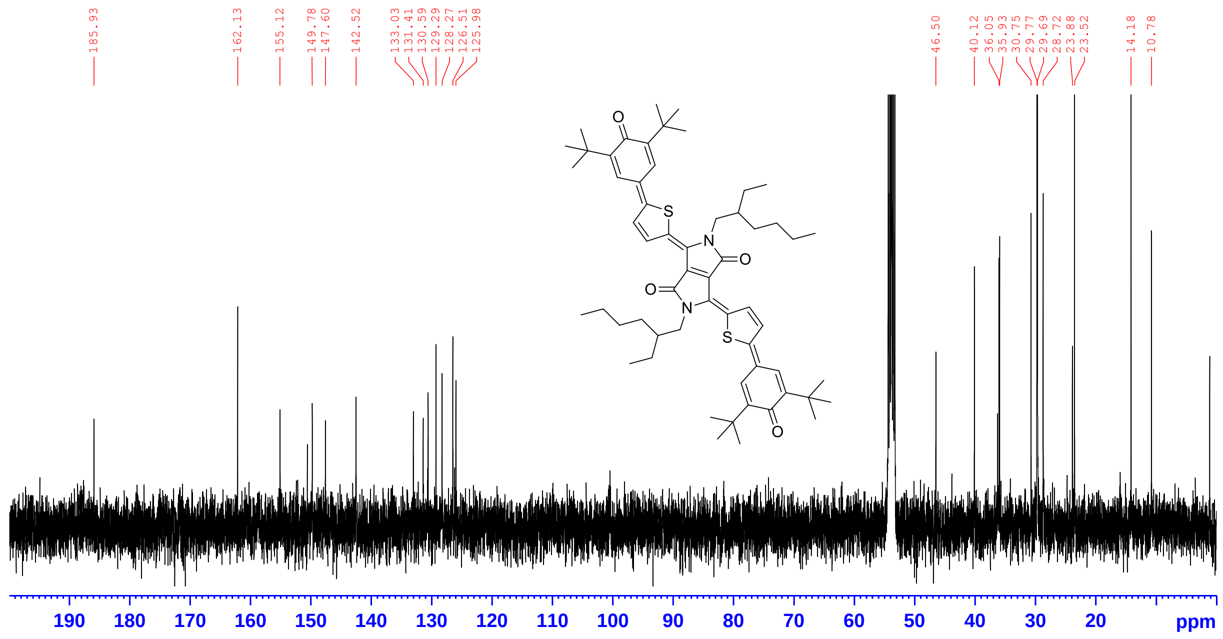

Fig. S19 ^{13}C NMR spectrum (101 MHz) of **DPP2q** in CD_2Cl_2 at 295 K.

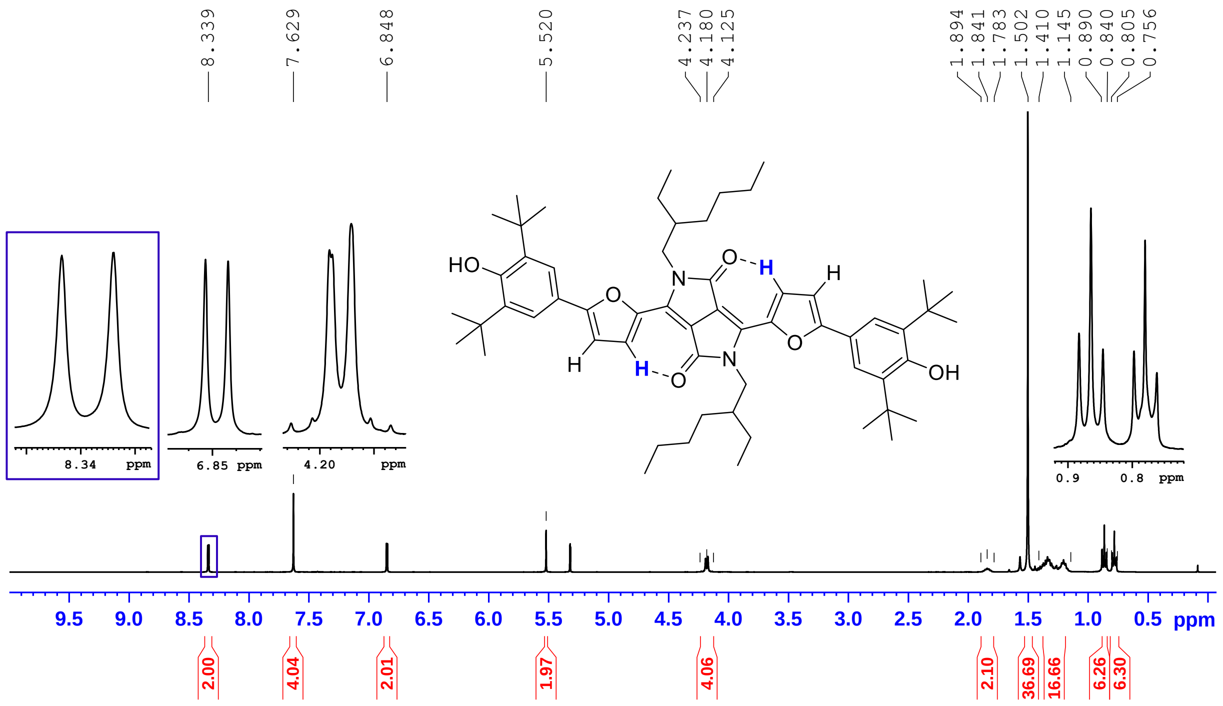

Fig. S20 ^{1}H NMR spectrum (400 MHz) of **DPP3** in CD_2Cl_2 at 295 K. Hydrogen bonded proton highlighted in blue.

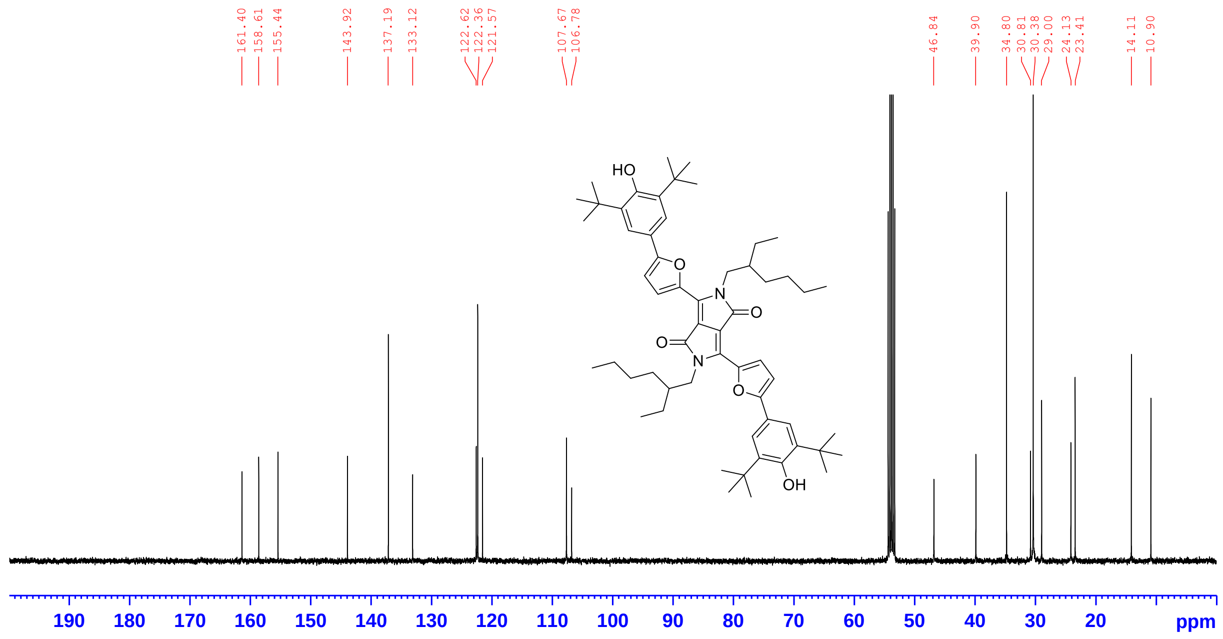

Fig. S21 ^{13}C NMR spectrum (101 MHz) of **DPP3** in CD_2Cl_2 at 295 K.

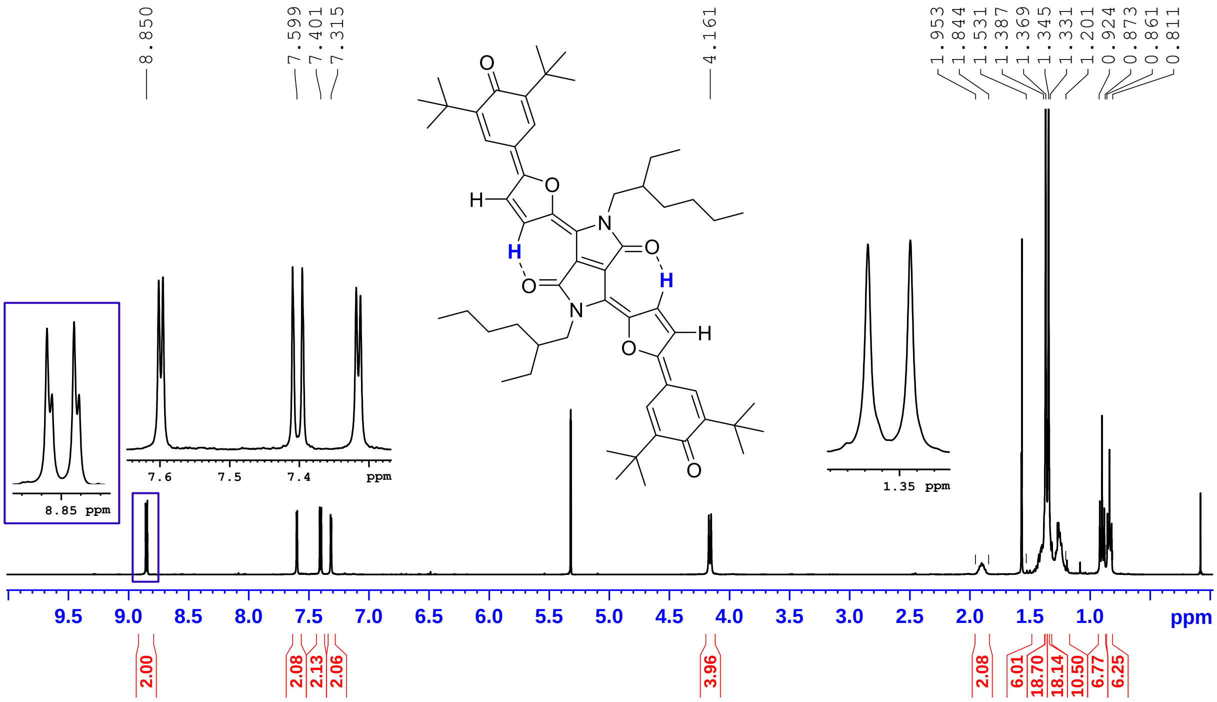

Fig. S22 ^{1}H NMR spectrum (400 MHz) of **DPP3q** in CD_2Cl_2 at 295 K. Hydrogen bonded proton highlighted in blue.

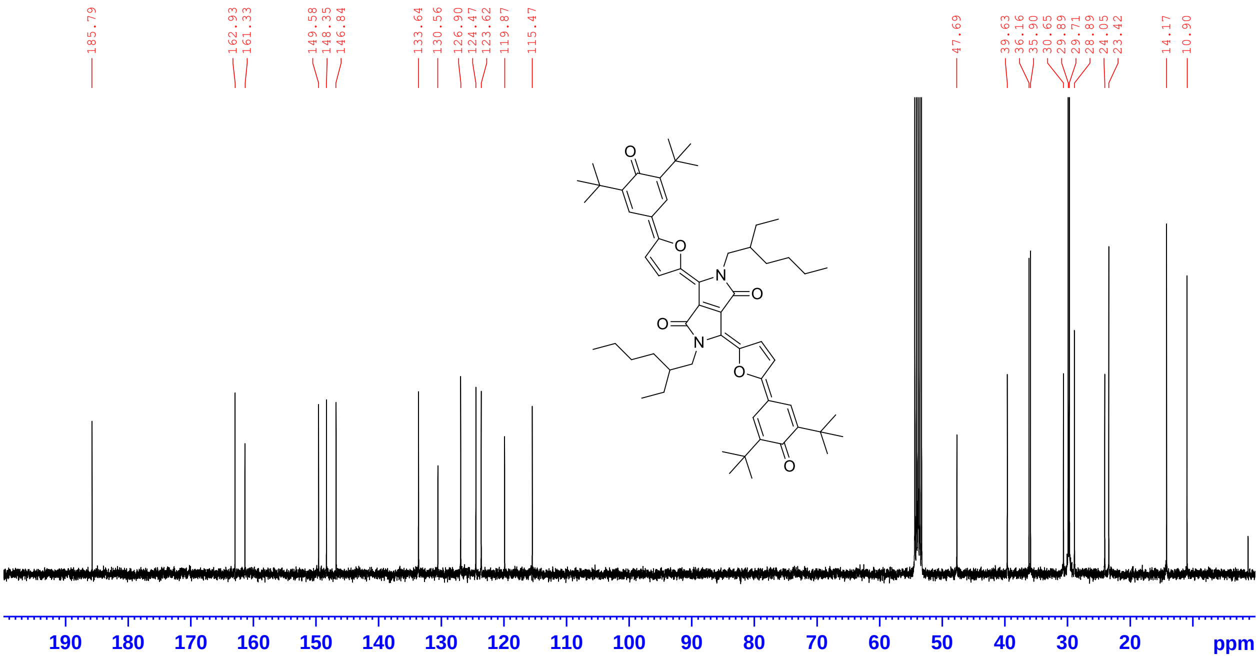

Fig. S23 ^{13}C NMR spectrum (101 MHz) of **DPP3q** in CD_2Cl_2 at 295 K.

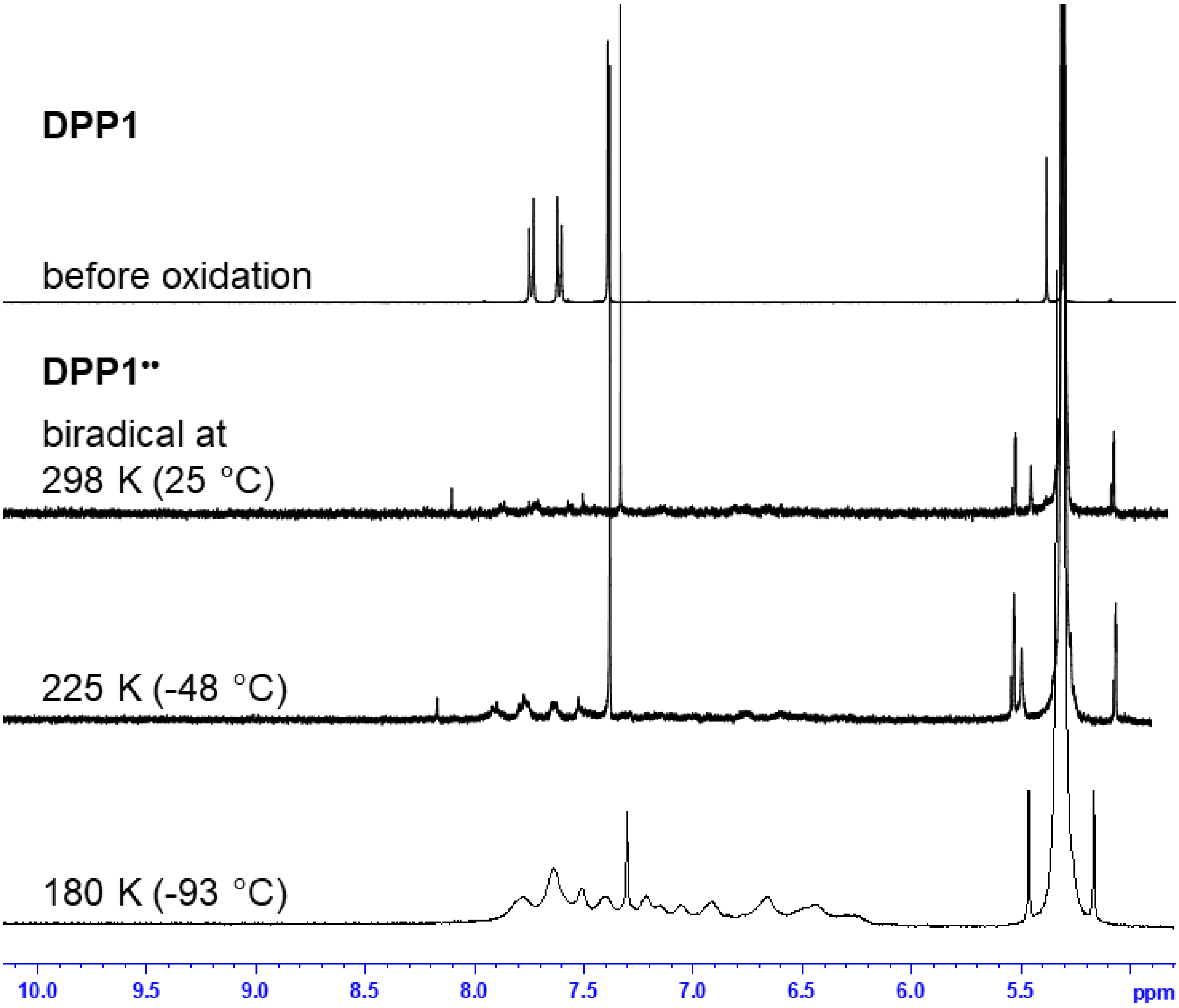

Fig. S24 Aromatic region of the ^{1}H NMR spectrum (600 MHz) of **DPP1** in CD_2Cl_2 before oxidation, and after oxidation to the biradical **DPP1••** at 298 K, 225 K and 180 K (from the top).

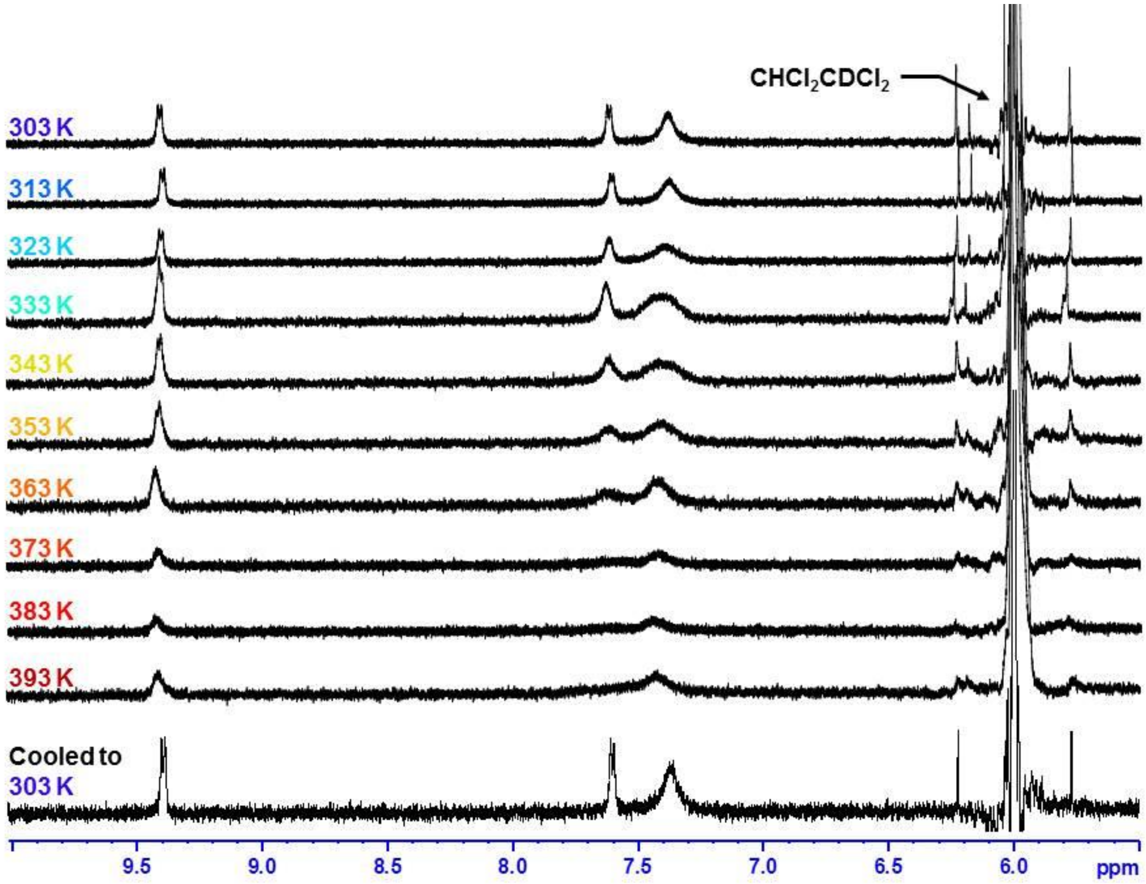

Fig. S25 Aromatic region of the ^{1}H NMR spectrum (400 MHz) of **DPP2q** in $C_2D_2Cl_4$ at various temperatures from 303 K to 393 K (from the top) and after cooling back to 303 K (bottom).

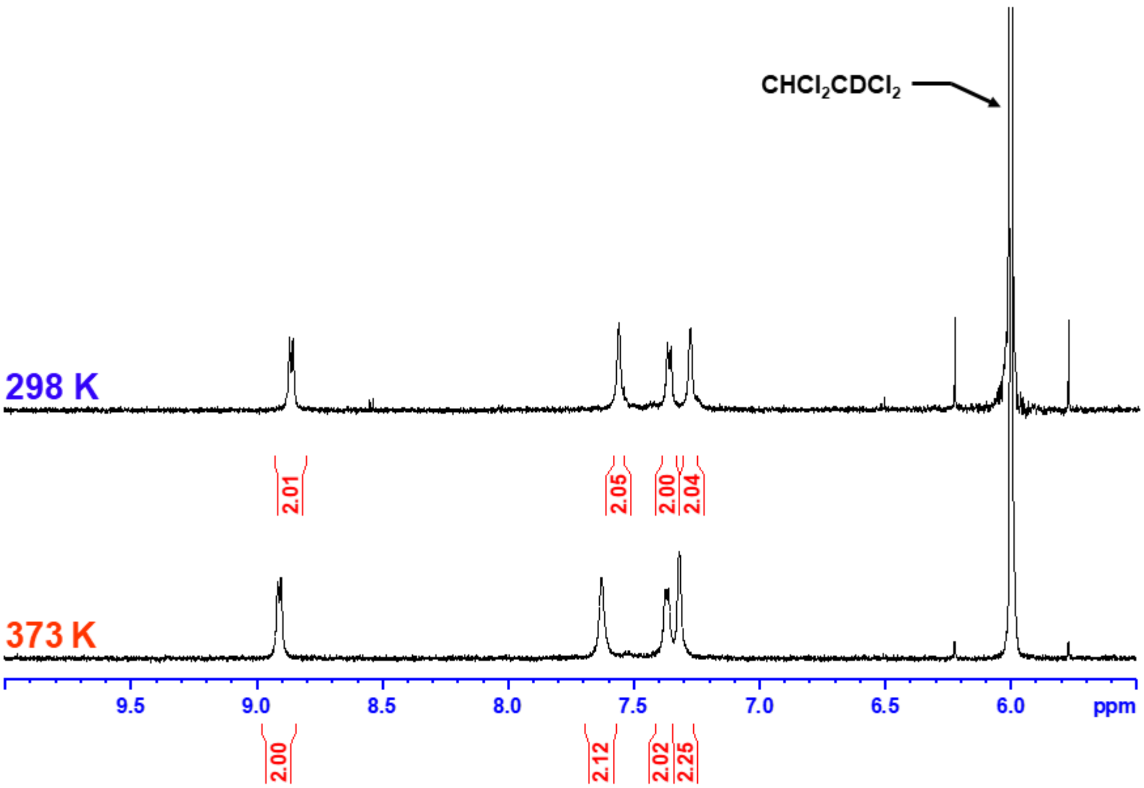

Fig. S26 Aromatic region of the ^{1}H NMR spectrum (400 MHz) of **DPP3q** in $C_2D_2Cl_4$ at 298 K (top) and at 373 K (bottom).

7 Band gap analysis and cyclic voltammetry (CV)

Tauc plot

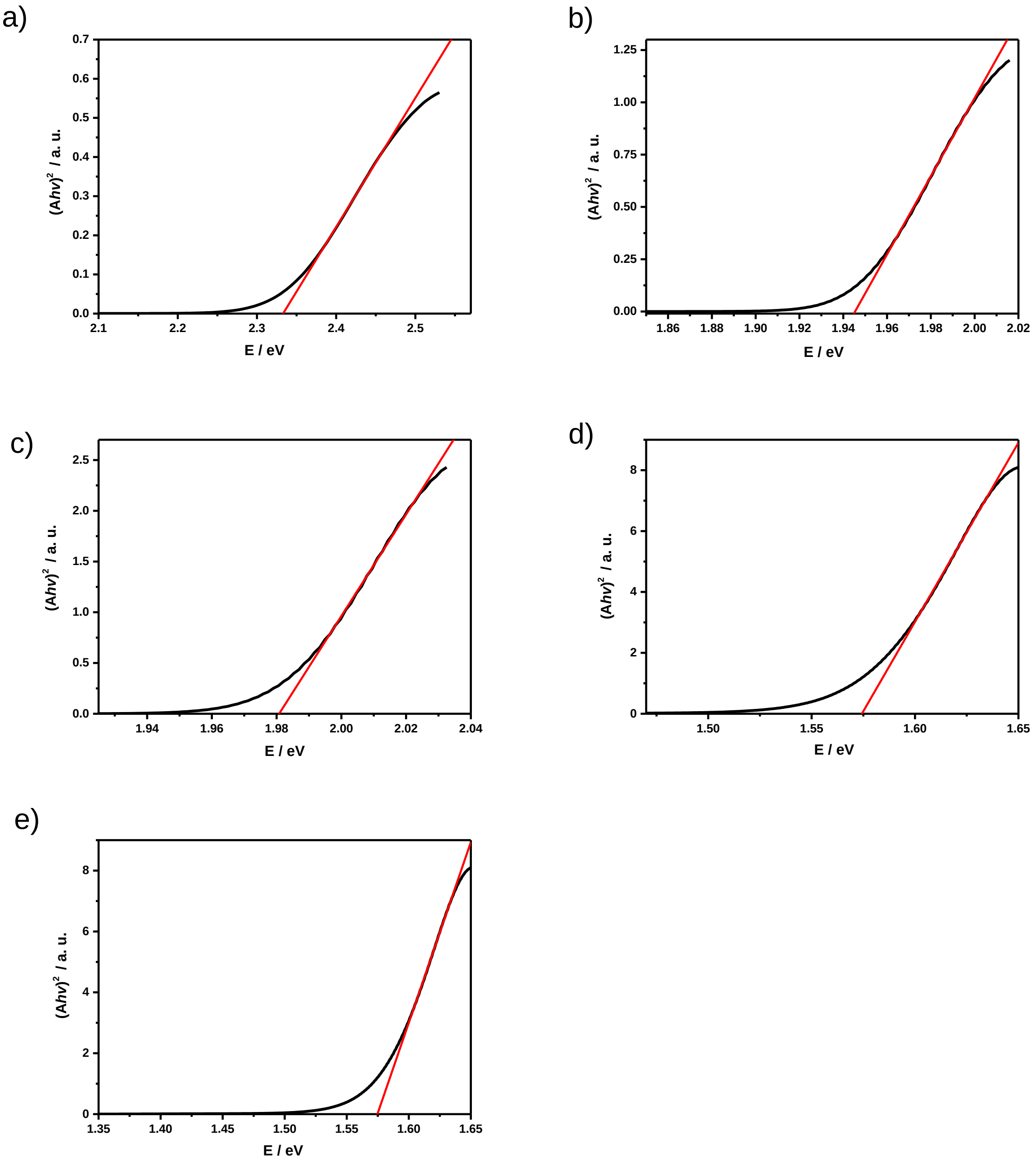

Fig. S27 Plot according to Tauc[S12-S14] and optical energy gap determination of (a) **DPP1**, (b) **DPP2**, (c) **DPP3**, (d) **DPP2q** and (e) **DPP3q**.

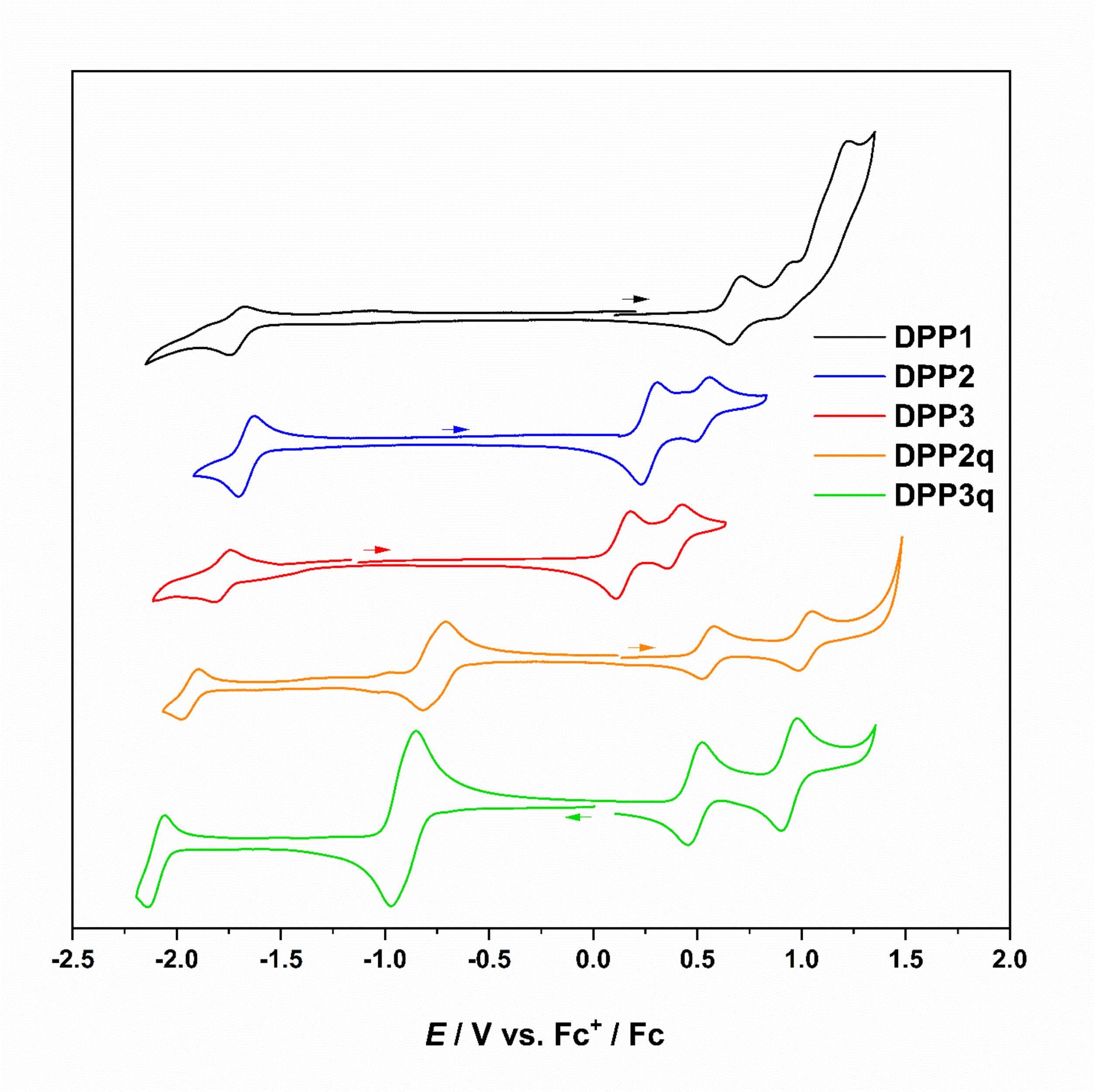

Fig. S28 Cyclic voltammetry of **DPP1, DPP2, DPP3, DPP2q** and **DPP3q** (from the top) in CH_2Cl_2 at room temperature (c ≈ 20 µM, electrolyte: 0.1 M $^{n}Bu_4NPF_6$, scan rate: 100 mVs^{-1}).

Table S4 Reduction potentials of **DPP1**, **DPP2**, **DPP3**, **DPP2q** and **DPP3q** versus the ferrocenium/ferrocene redox couple.

	$E_{1/2}^{Red2}$ [V]	$E_{1/2}^{Red1}$ [V]	$E_{1/2}^{Ox1}$ [V]	$E_{1/2}^{Ox2}$ [V]
**DPP1**		–1.711	0.683	
**DPP2**		–1.664	0.270	0.523
**DPP3**		–1.780	0.145	0.392
**DPP2q**	–1.937	–0.764	0.489	0.943
**DPP3q**	–2.098	–0.911	0.552	1.019

Fc^+/Fc = 0.00 V

Table S5 Frontier molecular orbital energies of **DPP1**, **DPP2**, **DPP3**, **DPP2q** and **DPP3q** as well as electrochemical and optical energy gap.

	***E*(HOMO)**[a] **[eV]**	***E*(LUMO)**[b] **[eV]**	$\boldsymbol{E_g}$ **[eV]**[c]	$\boldsymbol{E_g^{opt}}$ **[eV]**[d]
**DPP1**	–5.48	–3.09	2.39	2.33
**DPP2**	–5.07	–3.14	1.93	1.95
**DPP3**	–4.95	–3.02	1.93	1.98
**DPP2q**	–5.35	–4.04	1.31	1.57
**DPP3q**	–5.29	–3.89	1.40	1.57

a) *E*(HOMO) = –4.80 eV – $E_{1/2}^{Ox1}$. b) *E*(LUMO) = –4.80 eV – $E_{1/2}^{Red1}$. c) E_g = *E*(LUMO) – *E*(HOMO). d) E_g^{opt} according to Tauc.[S12-S14]

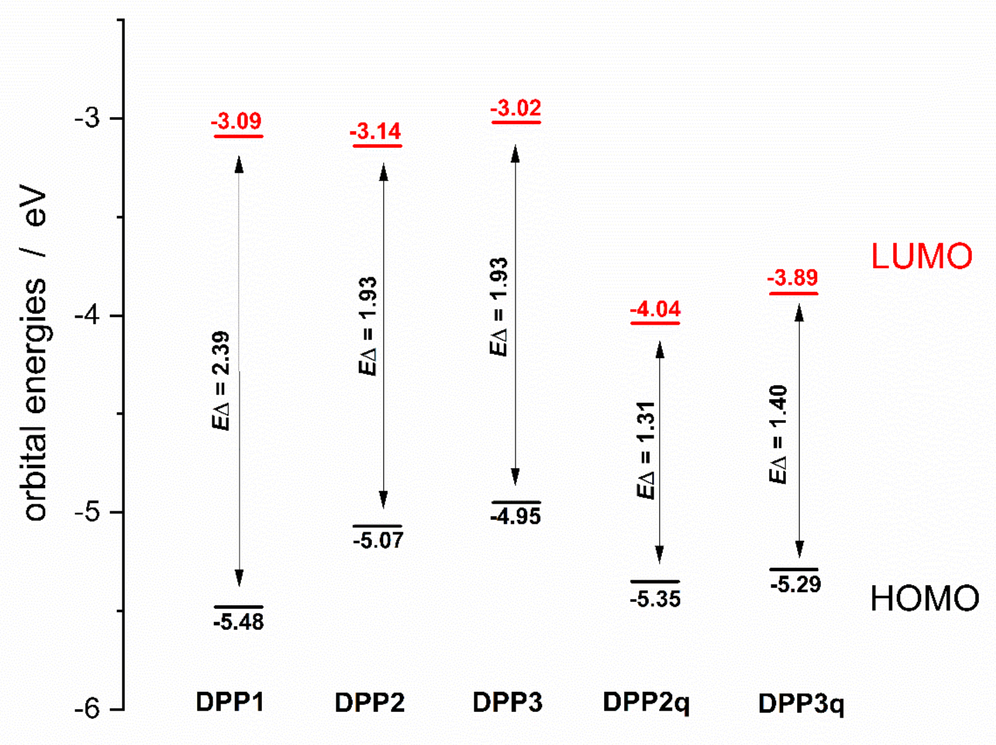

Fig. S29 Frontier orbital energies (in eV) of **DPP1**, **DPP2**, **DPP3**, **DPP2q** and **DPP3q** and electrochemical energy gap obtained from CV and differential pulse voltammetry (DP) data.

For the sake of completeness, the redox behavior of **DPP1**, **DPP2**, **DPP3**, **DPP2q** and **DPP3q** was additionally investigated by cyclic voltammetry (CV) experiments and the results compared to optical band gap analysis. The order of first oxidation potentials **DPP1** >> **DPP2** > **DPP3** can be explained by the more electron rich character of the heteroaromatic cores and is well in line with the higher autoxidation tendencies observed for **DPP2**$^{2-}$ and **DPP3**$^{2-}$ compared to **DPP1**$^{2-}$. Frontier orbital energies derived from CV are in good accordance with the optical band gap (Fig. S27–S29 and Table S5).

8 EPR spectroscopy

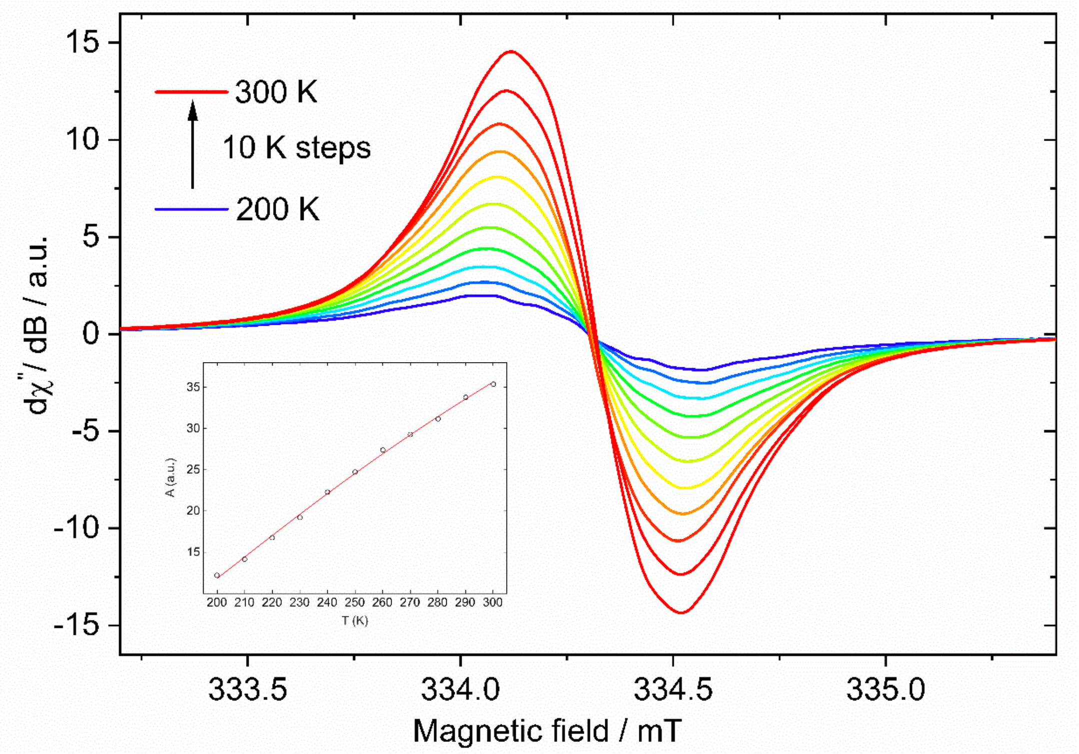

Fig. S30 Temperature dependence of the X-band EPR spectra of **DPP1••** in CH_2Cl_2 ($c \approx$ 1 mM). The signal is centered at g_{iso} = 2.0044 with a peak-to-peak line width of 3.8 G. Temperature dependence of the double integral EPR intensity (A) of **DPP1••** in CH_2Cl_2 wherein circles (○) represent the experimental results and the red line corresponds to the fit with the Bleaney-Bowers equation (inset).[S15]

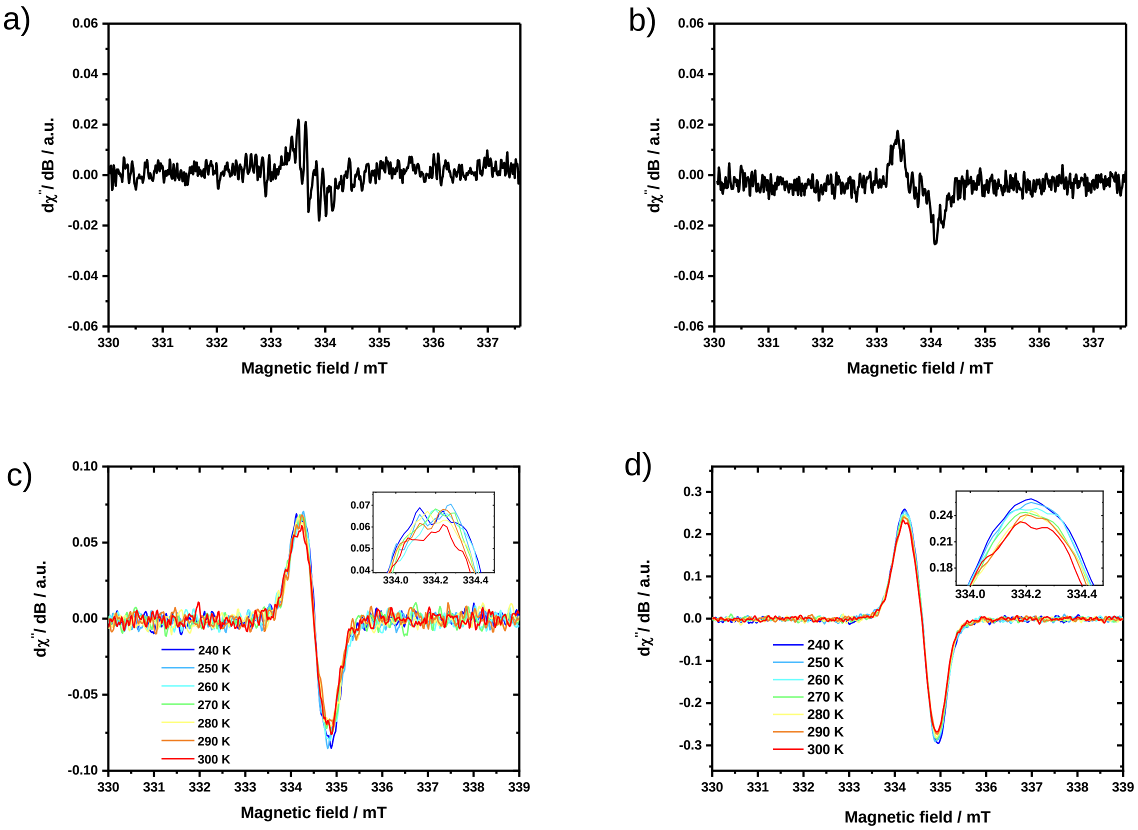

Fig. S31 Continuous-wave (CW) X-band EPR spectrum in CH_2Cl_2 solution ($c \approx 1$ mM) at 280 K of **DPP2q** (a) and **DPP3q** (b) as well as in the solid state at 240 to 300 K (diluted with KBr) of **DPP2q** (c) and **DPP3q** (d).

In the solid state VT-EPR measurements of **DPP2q** and **DPP3q**, no temperature dependence of the weak signal intensity could be observed. Accordingly, any fitting according to the Bleaney-Bowers equation[S15] is not justified as these signals are not attributable to triplet states of **DPP2q** and **DPP3q** but rather to impurities.

9 Computational chemistry

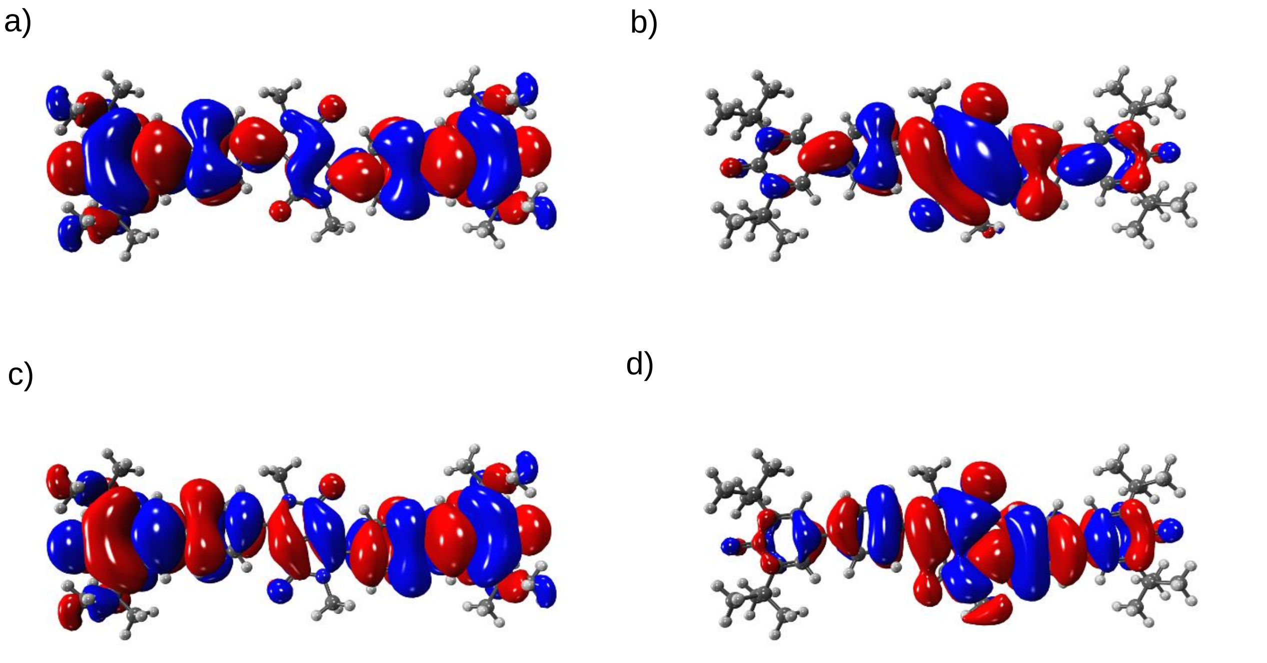

Fig. S32 Calculated frontier molecular orbital representations of **DPP1**$^{\bullet\bullet}$ (a) HOMO, (b) HOMO-1, (c) LUMO and (d) LUMO+1 (CASSCF(4,4)/def2-SVP level of theory).

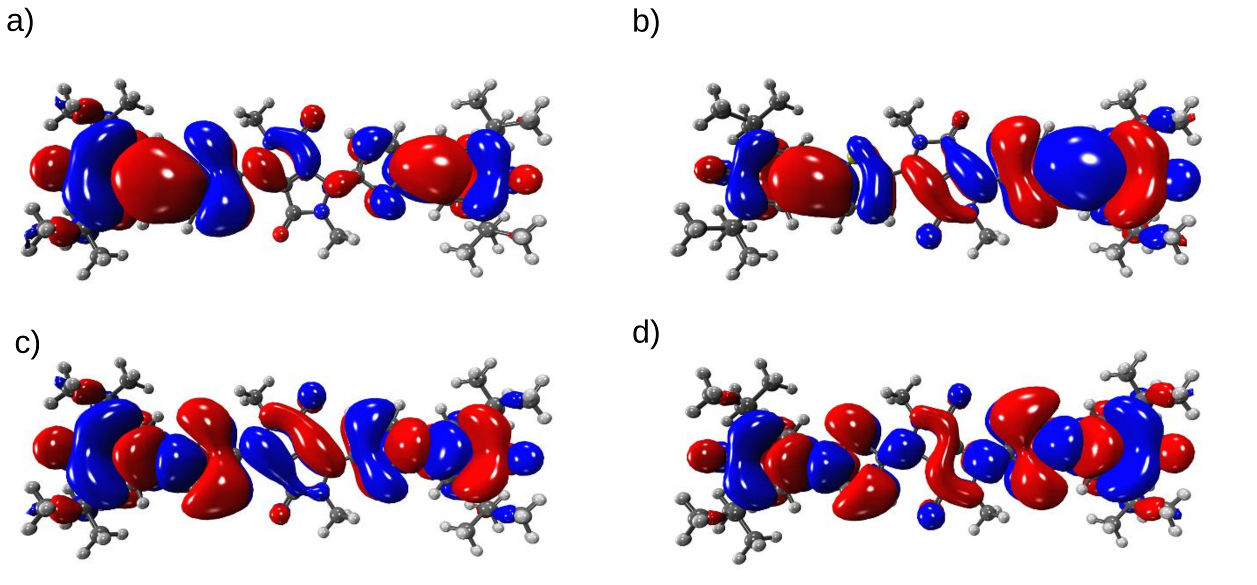

Fig. S33 Calculated frontier molecular orbital representations of **DPP2q** (a) HOMO, (b) HOMO-1, (c) LUMO and (d) LUMO+1 (CASSCF(4,4)/def2-SVP level of theory).

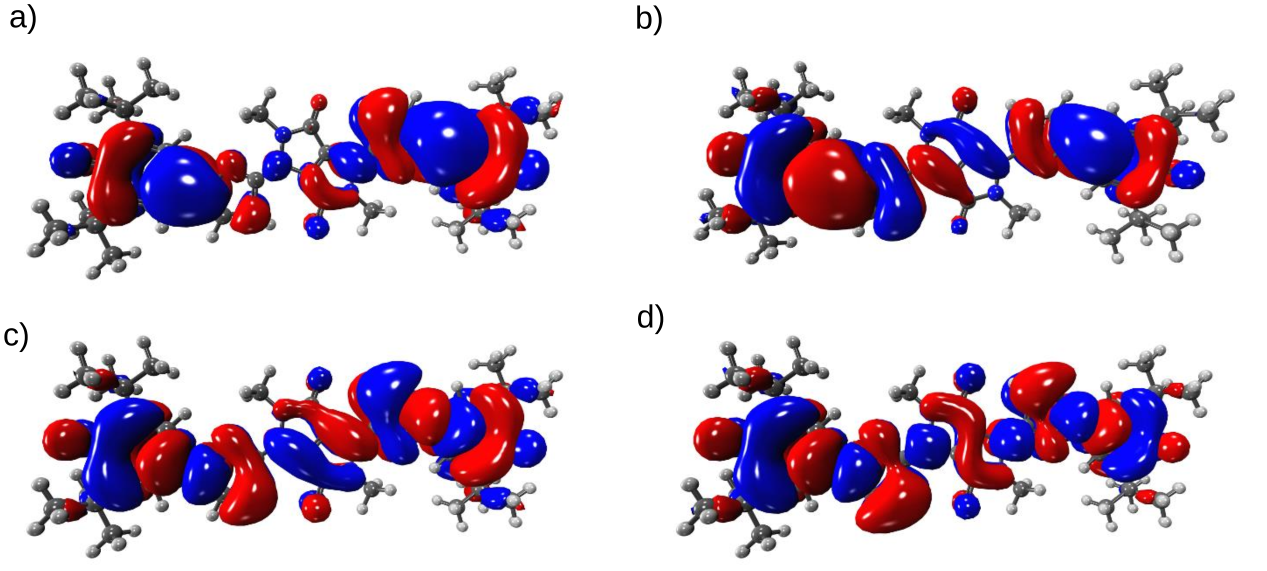

Fig. S34 Calculated frontier molecular orbital representations of **DPP3q** (a) HOMO, (b) HOMO-1, (c) LUMO and (d) LUMO+1 (CASSCF(4,4)/def2-SVP level of theory).

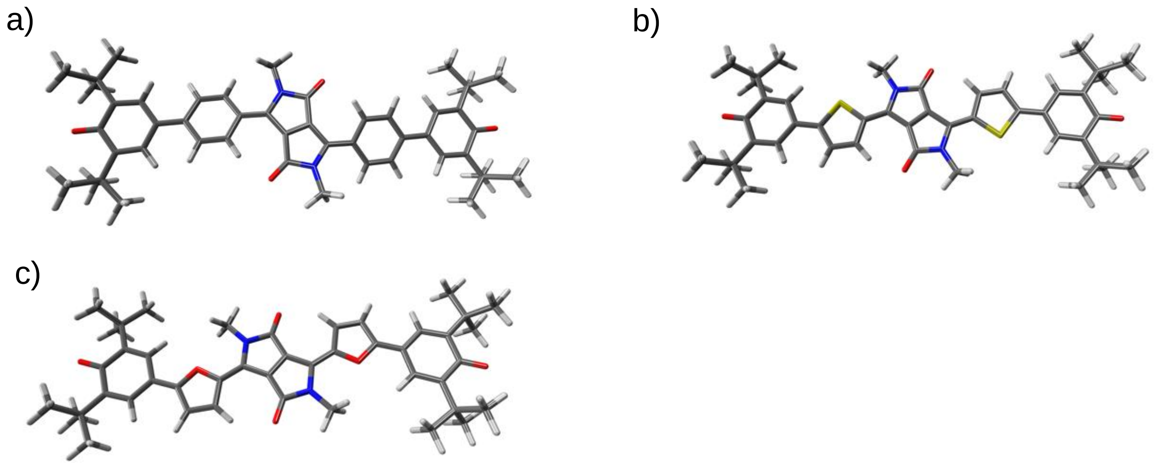

Fig. S35 Optimized geometries of (a) **DPP1**$^{\bullet\bullet}$, (b) **DPP2q** and (c) **DPP3q** (SF-TDDFT (BHHLYP/def2-SVP) level of theory).

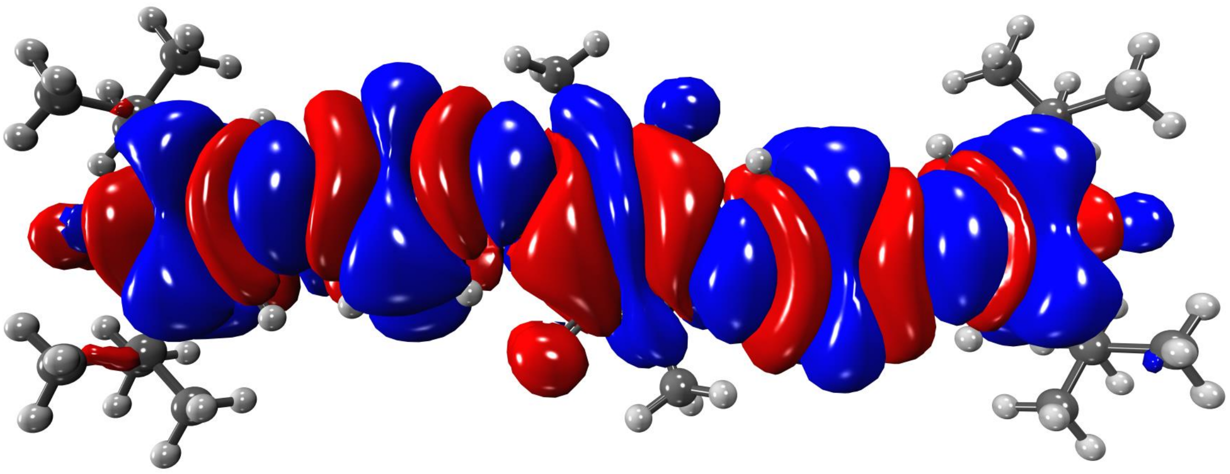

Fig. S36 Spin density distribution of **DPP1$^{\bullet\bullet}$** calculated on the CASSCF(4,4)/def2-SVP level of theory.

Table S6 Natural orbital occupation numbers (NOONs) of the frontier orbitals, singlet biradical character (y_0) and singlet-triplet energy gap (ΔE_{ST}) for **DPP1$^{\bullet\bullet}$**, **DPP2q** and **DPP3q** at the level of CASSCF(4,4)/def2-SVP.

	HONO-1	HONO	LUNO	LUNO+1	y_0	ΔE_{ST} (eV)
**DPP1$^{\bullet\bullet}$**	1.9111	1.1252	0.8754	0.0883	0.7540	0.0236
**DPP2q**	1.9227	1.9194	0.0818	0.0761	0.0035	1.2186
**DPP3q**	1.9555	1.9331	0.0707	0.0408	0.0025	1.3077

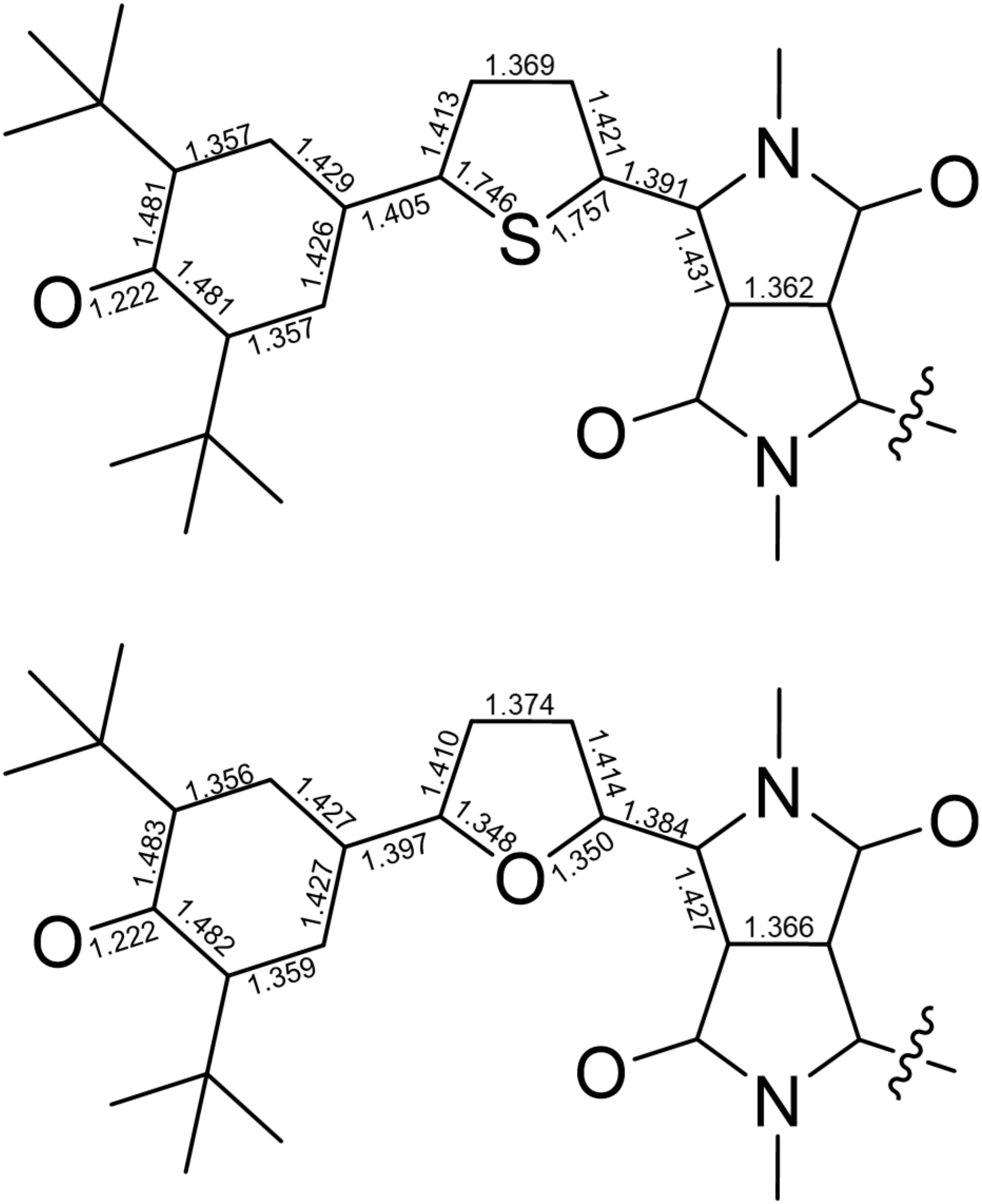

Fig. S37 Optimized *N,X-trans* geometries of **DPP2q** (top) and **DPP3q** (bottom) on the SF-TD-DFT (BHHLYP/def2-SVP) level of theory with selected bond lengths in Å.

10 References

S1 Y. Li, H. Li, H. F. Chen, Y. Wan, N. J. Li, Q. F. Xu, J. H. He, D. Y. Chen, L. H. Wang and J. M. Lu, *Adv. Funct. Mater.,* 2015, **25**, 4246–4254.

S2 L. J. Huo, J. H. Hou, H. Y. Chen, S. Q. Zhang, Y. Jiang, T. L. Chen and Y. Yang, *Macromolecules,* 2009, **42**, 6564–6571.

S3 C. H. Woo, P. M. Beaujuge, T. W. Holcombe, O. P. Lee and J. M. J. Fréchet, *J. Am. Chem. Soc.,* 2010, **132**, 15547–15549.

S4 R. Rausch, D. Schmidt, D. Bialas, I. Krummenacher, H. Braunschweig and F. Würthner, *Chem. Eur. J.,* 2018, **24**, 3420–3424.

S5 R. Sens and K. H. Drexhage, *J. Lumin.,* 1981, **24**/**25**, 709–712.

S6 G. Seybold and G. Wagenblast, *Dyes Pigm.,* 1989, **11**, 303–317.

S7 J. R. Lakowitz, *Principles of Fluorescence Spectroscopy*, 2nd ed., Kluwer Academic/Plenum, New York, 1999.

S8 Y. Shao, Z. Gan, E. Epifanovsky, A. T. B. Gilbert, M. Wormit, J. Kussmann, A. W. Lange, A. Behn, J. Deng, X. Feng, D. Ghosh, M. Goldey P. R. Horn, L. D. Jacobson, I. Kaliman, R. Z. Khaliullin, T. Kús, A. Landau, J. Liu, E. I. Proynov, Y. M. Rhee, R. M. Richard, M. A. Rohrdanz, R. P. Steele, E. J. Sundstrom, H. L. Woodcock III, P. M. Zimmerman, D. Zuev, B. Albrecht, E. Alguire, B. Austin, G. J. O. Beran, Y. A. Bernard, E. Berquist, K. Brandhorst, K. B. Bravaya, S. T. Brown, D. Casanova, C.-M. Chang, Y. Chen, S. H. Chien, K. D. Closser, D. L. Crittenden, M. Diedenhofen, R. A. DiStasio Jr., H. Dop, A. D. Dutoi, R. G. Edgar, S. Fatehi, L. Fusti-Molnar, A. Ghysels, A. Golubeva-Zadorozhnaya, J. Gomes, M. W. D. Hanson-Heine, P. H. P. Harbach, A. W. Hauser, E. G. Hohenstein, Z. C. Holden, T.-C. Jagau, H. Ji, B. Kaduk, K. Khistyaev, J. Kim, J. Kim, R. A. King, P. Klunzinger, D. Kosenkov, T. Kowalczyk, C. M. Krauter, K. U. Lao, A. Laurent, K. V. Lawler, S. V. Levchenko, C. Y. Lin, F. Liu, E. Livshits, R. C. Lochan, A. Luenser, P. Manohar, S. F. Manzer, S.-P. Mao, N. Mardirossian, A. V. Marenich, S. A. Maurer, N. J. Mayhall, C. M. Oana, R. Olivares-Amaya, D. P. O'Neill, J. A. Parkhill, T. M. Perrine, R. Peverati, P. A. Pieniazek, A. Prociuk, D. R. Rehn, E. Rosta, N. J. Russ, N. Sergueev, S. M. Sharada, S. Sharmaa, D. W. Small, A. Sodt, T. Stein, D. Stück, Y.-C. Su, A. J. W. Thom, T. Tsuchimochi, L. Vogt, O. Vydrov, T. Wang, M. A. Watson, J. Wenzel, A. White, C. F. Williams, V. Vanovschi, S. Yeganeh, S. R. Yost, Z.-Q. You, I. Y. Zhang, X. Zhang, Y. Zhou, B. R. Brooks, G. K. L. Chan, D. M. Chipman, C. J. Cramer, W. A. Goddard III, M. S. Gordon, W. J. Hehre, A. Klamt, H. F. Schaefer III, M. W. Schmidt, C. D. Sherrill, D. G. Truhlar, A. Warshel, X. Xua, A. Aspuru-Guzik, R. Baer, A. T. Bell, N. A. Besley, J.-D. Chai, A. Dreuw, B. D. Dunietz, T. R. Furlani, S. R. Gwaltney, C.-P. Hsu, Y. Jung, J. Kong, D. S. Lambrecht, W. Liang, C. Ochsenfeld, V. A. Rassolov, L. V. Slipchenko, J. E. Subotnik, T. Van Voorhis, J. M. Herbert, A. I. Krylov, P. M. W. Gill and M. Head-Gordon. Advances in molecular quantum chemistry contained in the Q-Chem 4 program package. [*Mol. Phys.,* 2015, **113**, 184–215].

S9 T. Shiozaki, Wiley Interdisciplinary Reviews: Computational Molecular Science, 2018, 8, e1331.

S10 G. M. Sheldrick, *Acta Cryst. A,* 2008, **64**, 112–122.

S11 S. Stoll and A. Schweiger, *J. Magn. Reson*., 2006, **178**, 42–55.

S12 J. Tauc, R. Grigorovici and A. Vancu, *Phys. Status. Solidi.,* 1966, **15**, 627–637.

S13 I. Seguy, P. Jolinat, P. Destruel, R. Mamy, H. Allouchi, C. Courseille, M. Cotrait and H. Bock, *ChemPhysChem,* 2001, **2**, 448–452.

S14 C. M. Cardona, W. Li, A. E. Kaifer, D. Stockdale and G. C. Bazan, *Adv. Mater.,* 2011, **23**, 2367–2371.

S15 B. Bleaney and K. D. Bowers, *Proc. R. Soc. A,* 1952, **214**, 451–465.
